# Dietary fibre supplementation enhances radiotherapy tumour control and alleviates intestinal radiation toxicity

**DOI:** 10.1186/s40168-024-01804-1

**Published:** 2024-05-14

**Authors:** Chee Kin Then, Salome Paillas, Aliu Moomin, Mariya D. Misheva, Rachel A. Moir, Susan M. Hay, David Bremner, Kristine S. Roberts (nee Nellany), Ellen E. Smith, Zeynab Heidari, Daniel Sescu, Xuedan Wang, Alejandro Suárez-Bonnet, Nadine Hay, Sarah L. Murdoch, Ryoichi Saito, Elaina S. R. Collie-Duguid, Shirley Richardson, Simon L. Priestnall, Joan M. Wilson, Mahalakshmi Gurumurthy, Justine S. Royle, Leslie M. Samuel, George Ramsay, Katherine A. Vallis, Kevin R. Foster, James S. O. McCullagh, Anne E. Kiltie

**Affiliations:** 1 Department of Oncology, University of Oxford, Oxford, UK; 2 Present Address: Department of Radiation Oncology, Shunag Ho Hospital, Taipei Medical University, New Taipai City, Taiwan; 3 Present Address: The Rowett Institute, University of Aberdeen, Aberdeen, UK; 4 Aberdeen Cancer Centre, University of Aberdeen, Aberdeen, UK; 5 Chemistry Research Laboratory, Department of Chemistry, Mansfield Road, University of Oxford, Oxford, UK; 6 Oxford Centre for Microbiome Studies, Kennedy Institute of Rheumatology, University of Oxford, Oxford, UK; 7 Department of Oncology, Aberdeen Royal Infirmary, Aberdeen, UK; 8 NHS Grampian Biorepository, Aberdeen Royal Infirmary, Aberdeen, UK; 9 Centre for Genome Enabled Biology and Medicine, School of Medicine Medical Sciences and Nutrition, University of Aberdeen, Aberdeen, UK; 10 The School of Medicine, Medical Sciences and Nutrition, University of Aberdeen, Aberdeen, UK; 11 Department of Biology, University of Oxford, Oxford, UK; 12 Department of Biochemistry, University of Oxford, Oxford, UK; 13 Department of Pathobiology and Population Sciences, The Royal Veterinary College, London, UK; 14 Lineberger Comprehensive Cancer Centre, University of North Carolina at Chapel Hill, Chapel Hill, USA; 15 Present Address: The Department of Urology, Kyoto University, Kyoto, Japan; 16 Department of Gynaecological Oncology, Aberdeen Royal Infirmary, Aberdeen, UK; 17 Department of Urology, Aberdeen Royal Infirmary, Aberdeen, UK; 18 Health Services Research Unit, University of Aberdeen, Aberdeen, UK

**Keywords:** Radiotherapy, Dietary fibre, Gut microbiota, Short-chain fatty acids, Isoferulic acid, Cancer, Immunomodulation

## Abstract

**Background:**

Non-toxic approaches to enhance radiotherapy outcomes are beneficial, particularly in ageing populations. Based on preclinical findings showing that high-fibre diets sensitised bladder tumours to irradiation by modifying the gut microbiota, along with clinical evidence of prebiotics enhancing anti-cancer immunity, we hypothesised that dietary fibre and its gut microbiota modification can radiosensitise tumours via secretion of metabolites and/or immunomodulation. We investigated the efficacy of high-fibre diets combined with irradiation in immunoproficient C57BL/6 mice bearing bladder cancer flank allografts.

**Result:**

Psyllium plus inulin significantly decreased tumour size and delayed tumour growth following irradiation compared to 0.2% cellulose and raised intratumoural CD8^+^ cells. Post-irradiation, tumour control positively correlated with Lachnospiraceae family abundance. Psyllium plus resistant starch radiosensitised the tumours, positively correlating with *Bacteroides* genus abundance and increased caecal isoferulic acid levels, associated with a favourable response in terms of tumour control. Psyllium plus inulin mitigated the acute radiation injury caused by 14 Gy. Psyllium plus inulin increased caecal acetate, butyrate and propionate levels, and psyllium alone and psyllium plus resistant starch increased acetate levels. Human gut microbiota profiles at the phylum level were generally more like mouse 0.2% cellulose profiles than high fibre profiles.

**Conclusion:**

These supplements may be useful in combination with radiotherapy in patients with pelvic malignancy.

Video Abstract

**Supplementary Information:**

The online version contains supplementary material available at 10.1186/s40168-024-01804-1.

## Introduction

Radiation ± concurrent chemotherapy has an important role in the treatment of pelvic malignancies, such as bladder, prostate, gynecological and colorectal cancers, as it allows organ preservation [1, 2]. However, elderly people, who are more vulnerable to treatment-related toxicity, make up the majority of patients with pelvic cancers. Interventions that can enhance response, and organ preservation, without additional toxicity are needed urgently.

The gut microbiota has a significant, potentially beneficial, impact on human health and disease [3, 4]. Its composition was associated with response to immunotherapy in cancer patients and causality has been demonstrated preclinically [5, 6]. Gopalakrishnan et al. showed that melanoma patients responding to anti-programmed cell death 1 protein (PD-1) immunotherapy had significantly higher alpha diversity (a measure of taxonomic diversity within communities) of the gut microbiota and Ruminococcaceae family abundance [5]. Studies also found a similar association between higher alpha diversity and chemoradiation response in cervical cancer patients [7] and in colorectal patients [8]. Preclinical mouse models also support the hypothesis that the gut microbiota can modulate the efficacy of chemotherapy [9, 10] and radiotherapy [11, 12]. The enhancement of anti-cancer treatment can be achieved via immunomodulation and/or secretion of metabolites, including butyrate [13, 14], inosine [15] and trimethylamine-N-oxide (TMAO) [16]. Dietary fibre is fermented by the gut microbiota to produce short-chain fatty acids (SCFAs) and a broad range of other metabolites [17].

An effective way to modify the gut microbiota is by adding dietary fibre supplements to the diet that can alter human gut bacterial diversity and faecal SCFA production rapidly [18]. Mounting preclinical evidence shows that dietary fibre can slow tumour growth [19, 20] and enhance the efficacy of anti-cancer treatments [21]. Spencer et al. showed that melanoma patients with high dietary fibre consumption had a better response to immunotherapy [22], and this had a protective effect against gastrointestinal toxicity during pelvic radiotherapy in a randomised-controlled clinical trial [23]. Therefore, we hypothesised that dietary fibre could enhance the efficacy of radiotherapy through gut microbiota modulations, enhanced immune responses and/or metabolite production.

The main alternative strategy *in vivo* has used antibiotics in studying the role of the gut microbiota in relation to radiotherapy efficacy [11, 24]. These preclinical cancer models showed that antibiotics can diminish [24] or increase [11] the radiotherapy effect with depletion of specific commensal bacteria. To our knowledge, there are no human studies of dietary fibre supplements in the context of enhancing tumour response to pelvic radiotherapy. Consistent with a previous study [25], we found that dietary fibre enhanced radiosensitivity in an immunocompromised mouse bladder tumour model [26] and we were the first to show that this was associated with modification of the gut microbiota. Dietary fibre has been shown to alleviate radiation-related diarrhoea [27] while also increasing bacterial fermentation and metabolite production [28], and enhancing anti-tumour immune responses [22].

In this study, we used immunocompetent C57BL/6 mice to study the effects of dietary fibre supplementation, alone and in combination with ionising radiation (IR), on tumour response and radiation normal tissue toxicity. We showed that the systemic effects of the gut microbiota in terms of secreted metabolites and immune responses, due to dietary fibre modification, could be exploited in conjunction with radiotherapy to achieve tumour radiosensitisation and amelioration of normal tissue effects.

## Results

### Psyllium plus resistant starch (RS) or psyllium plus inulin significantly decreased tumour size

**Fig. 1:**
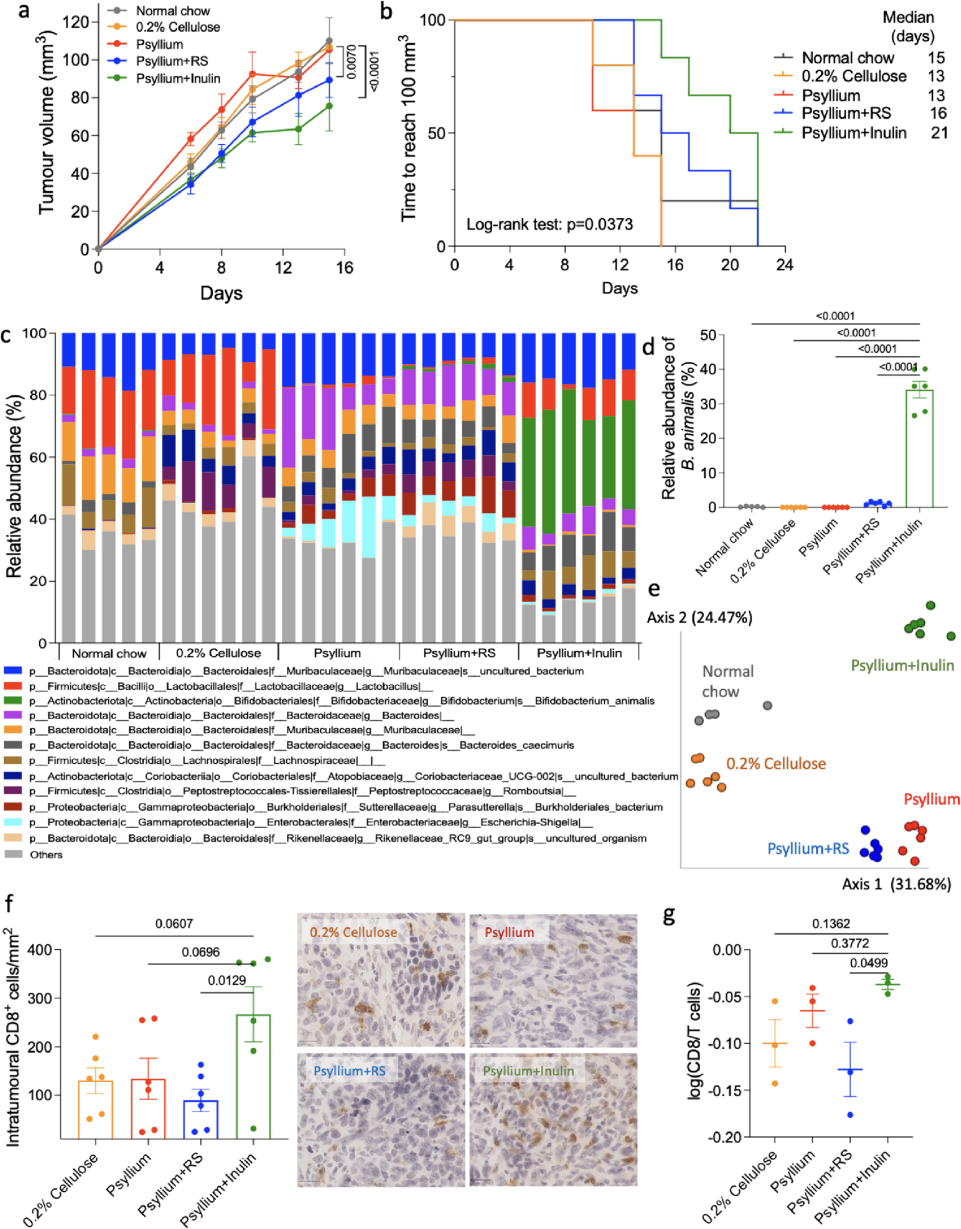
Psyllium plus RS and psyllium plus inulin cause increased tumour growth delay in bladder cancer cell allografts. **a** Treatment of UPPL1591 allografts with normal chow, low fibre (0.2% cellulose) and high fibre diets including psyllium, psyllium plus RS or inulin (*n* = 6 for each group). Slopes of tumour curves were calculated by linear regression to represent tumour growth rates. **b** Kaplan–Meier survival curve of mice with UPPL1591 allografts showing plots of time to tumour volume of 100 mm^3^. **c** Phylogenetic composition of the faecal microbiota when tumours reached 100 mm^3^. **d** Relative abundance of *Bifidobacterium animalis* of high fibre groups compared to psyllium plus inulin group. **e** Principal coordinate analysis of faecal microbiotas using Bray-Curtis dissimilarity. ADONIS test was used to confirm the existence of significant group differences in terms of gut microbiota composition. **f** IHC staining of CD8^+^ cells to assess the numbers of cytotoxic T cells in tumours (*n* = 6/group) and the representative images. One-way ANOVA with Bonferroni’s multiple comparison test was used to compare the means of different dietary groups. **g** NanoString analysis of CD8^+^ cells over T cells to assess the populations of cytotoxic T cells in tumours (*n* = 3/group). **a**, **d**, **f** and **g** One-way ANOVA with Bonferroni’s multiple comparison test was used to compare the means among different dietary groups. Data are presented as mean ± SEM

To study the tumour suppressive effects of dietary fibre, UPPL1591 mouse bladder tumour cells were inoculated subcutaneously on the same day as starting to feed the mice with normal chow or a modified diet, namely, 0.2% cellulose, psyllium (used to mitigate side effects after radiotherapy), psyllium plus resistant starch (RS; butyrate-producing fibre) or psyllium plus inulin (readily fermentable fibre and potential radiosensitiser [26]). Psyllium plus either RS (*p* = 0.0070) or inulin (*p* < 0.0001) significantly delayed tumour growth compared to the 0.2% cellulose group (Fig. 1a). The mean tumour sizes were 80 mm^3^ and 60 mm^3^ in psyllium plus RS and psyllium plus inulin, respectively, at the time that the 0.2% cellulose and psyllium groups reached 100 mm^3^. With time to reach 100 mm^3^ as the survival analysis endpoint, log-rank testing showed median times were significantly different (*p* = 0.0373) among dietary groups, at 15, 13, 13, 16 and 21 days for normal chow, 0.2% cellulose, psyllium, psyllium plus RS and psyllium plus inulin, respectively (Fig. 1b).

The gut microbiota profiles showed that the three taxa with the highest abundances were a *Muribaculaceae* uncultured species, *Lactobacillus* species and *Bifidobacterium animalis* (Fig. 1c). Psyllium plus inulin increased the *B. animalis* abundance up to 35% which was significantly higher than in all the other dietary groups (*p* < 0.0001; Fig. 1d). *Bacteroides* genus was significantly enriched in mice fed the psyllium diet (*p* < 0.001 for normal chow and 0.2% cellulose; Supplementary Figure S1a and S1b), which included *Bacteroides caecimuris* (*p* = 0.0008 for normal chow and < 0.0001 for 0.2% cellulose; Supplementary Figure S1b). Mice fed with psyllium plus RS had higher abundances of *Parasuterella* (the top genus enriched by this diet; *p* = 0.0029 for psyllium and < 0.0001 for psyllium plus inulin) and *Faecalibaculum* (butyrate-producing bacteria that possess anti-tumorigenic properties [29]; *p* = 0.0039 for psyllium and < 0.0001 for psyllium plus inulin) genus (Supplementary Figure S2a and S2b). The alpha diversity was significantly lower in the psyllium plus inulin group in terms of Shannon’s diversity index and Peilou’s evenness (*p* < 0.0001; Supplementary Figure S2c). A notable cluster effect of all dietary groups was found (*R*^2^ = 0.8052, Pr(>F) = 0.001; Fig. 1e).

It is acknowledged that the cytotoxic T cell is the most powerful effector cell in anti-tumour immunity [30]. The number of CD8^+^ T cells was significantly increased by psyllium plus inulin compared to psyllium plus RS (*p* = 0.0129; Fig. 1f). Immune cell profile by a NanoString platform also showed a consistent result with the ratio of CD8^+^ T cells to total T cells enriched in psyllium plus inulin group tumours (*p* = 0.0499; Fig. 1g), this being the only immune cell type increased (Supplementary Figure S3a). There was a trend for psyllium plus inulin-treated mice having higher levels of systemic helper T and cytotoxic T cells compared to psyllium plus RS-treated (*p* = 0.0882 for CD4^+^ T cells and *p* = 0.1085 for CD8^+^ T cells; Supplementary Figure S3b), consistent with the local tumour immune analysis. Metabolite profiles were also modified by the dietary fibre with differences between psyllium plus RS, psyllium plus inulin and psyllium alone (*R*^2^ = 0.6049, Pr(>F) = 0.001 for left panel included normal chow, and *R*^2^ = 0.4972, Pr(>F) = 0.001 for right panel; Supplementary Figure S4a) and inosine (*q* = 2.77E-09; the q-value represents an adjusted *p* value obtained through the false discovery rate (FDR) method for multiple testing) was significantly enriched in the caecal contents of mice fed with psyllium plus inulin along with N-acetyl ornithine (*q* = 4.65E−08), N6-acetyl-L-lysine (*q* = 1.27E−06) and homocitric acid (*q* = 1.51E−06; Supplementary Figure S4b). Sumiki’s acid (*q* = 7.63E−04), 4-(2-aminophenyl)-2,4-dioxobutanoic acid (*q* = 1.40E−03), allantoin (*q* = 3.52E−03) and cysteine-S-sulfate (*q* = 3.60E−03) were enriched in mice fed with psyllium plus RS.

### Psyllium plus RS or psyllium plus inulin combined with IR increased growth delay in bladder tumours

**Fig. 2:**
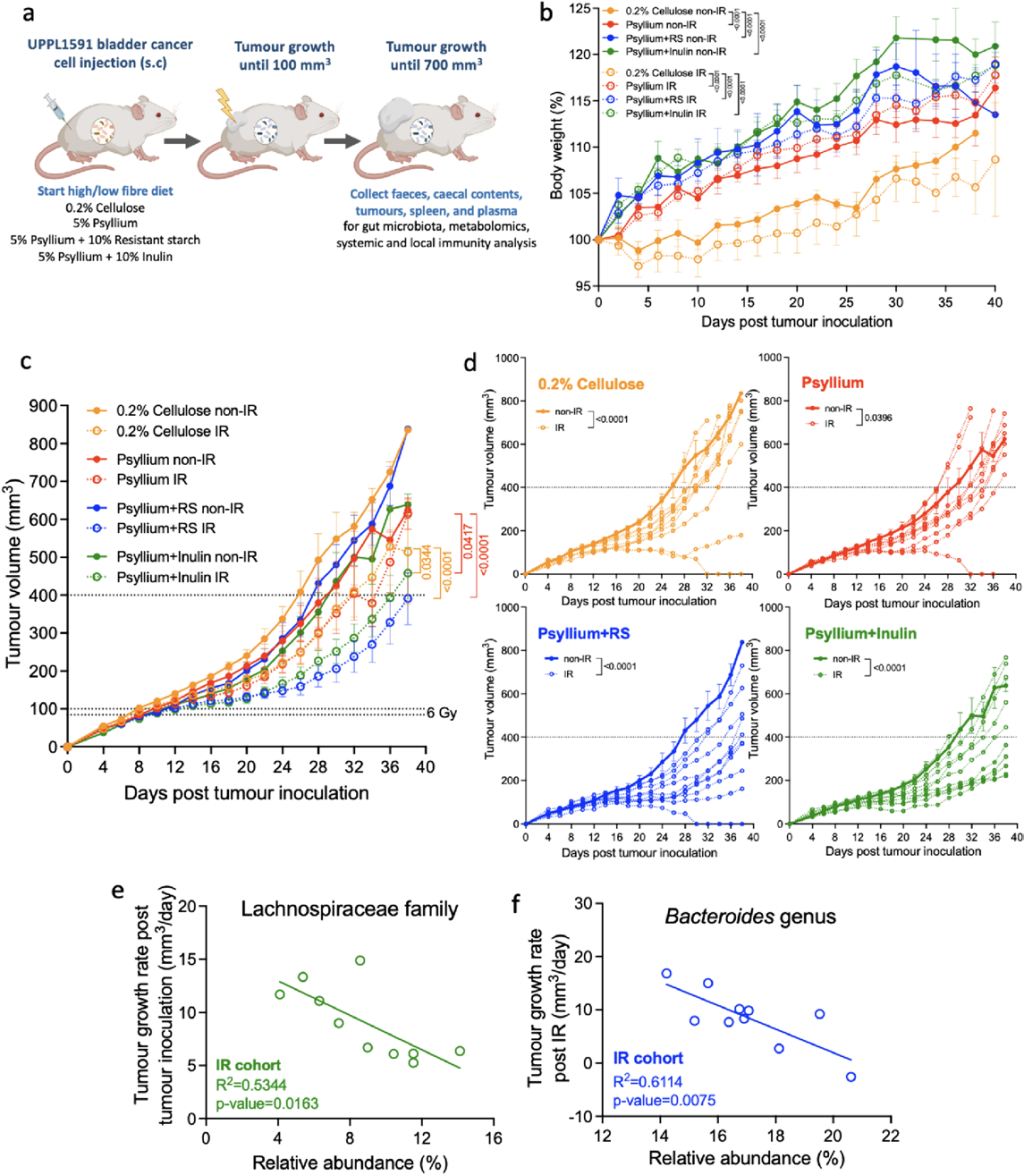
Psyllium plus RS or psyllium plus inulin enhanced tumour control combined with IR in bladder cancer cell allografts. **a** UPPL1591 bladder cancer cells were injected subcutaneously in the flank of C57BL/6 mice on the same day that they started on a low or high-fibre diet (*n* = 6/group). Tumours were irradiated with 6 Gy IR when they reached 80–100 mm^3^ and monitored until 700 mm^3^. Retrieved from https://app.biorender.com/biorender-templates. **b** Body weight changes of mice fed with modified diets across the whole experiment. The weight on the first day of tumour inoculation was set to 100%. **c** Growth curves of tumours that were irradiated with 6 Gy when they reached 80–100 mm^3^ and monitored until 700 mm^3^. Day 0 was the day of tumour inoculation and when the mice started taking modified diets. The overall growth curves of non-irradiated and irradiated mice were plotted in solid and dotted lines, respectively. Two-way ANOVA with Bonferroni’s multiple comparison test was used to compare the means of different dietary groups while taking irradiation into account. **d** Individual tumour growth curves stratified into non-IR and IR. Solid lines were the mean of tumour growth curves of non-IR mice and dotted lines were individual growth curves of IR mice. Slopes of tumour curves were calculated by linear regression to represent tumour growth rates. **e** Correlation between the Lachnospiraceae family relative abundance versus the tumour growth in non-IR and IR cohorts of psyllium plus inulin. Tumour curve slopes were calculated by linear regression to represent tumour growth rates. **f** Correlation between the *Bacteroides* species relative abundance versus the tumour growth in IR cohort of psyllium plus RS. **e**, **f** The associations were assessed using the Pearson correlation method

To study the radiosensitising effect of dietary fibre, we irradiated (IR) the tumours with 6 Gy IR when they reached 80–100 mm^3^, with all other parameters as per the previous diet alone UPPL1591 tumour experiment (Fig. 2a). This experiment consisted of 60 mice in four groups (*n* = 15 per group) fed modified diets either without (*n* = 5) or with (*n* = 10) irradiation. Across the whole experiment, all mice stably gained weight in the low and high-fibre diets groups (Fig. 2b). All psyllium diets maintained the rate of weight increase compared to 0.2% cellulose and the *p* value was < 0.0001 for psyllium, psyllium plus RS and psyllium plus inulin for both the non-IR and IR cohorts. After receiving IR when the tumours reached 80–100 mm^3^, IR cohorts of psyllium plus RS (*p* = 0.0341) or inulin (*p* = 0.0008) experienced slower rates of weight gain compared to their non-IR controls (Supplementary Figure S5a and S5b).

In all dietary groups, IR slowed tumour growth compared with their non-IR controls (*p* values were < 0.0001 for 0.2% cellulose, psyllium plus RS or psyllium plus inulin, and 0.0396 for psyllium; Fig. 2c, d). In the IR cohorts, psyllium plus RS or psyllium plus inulin significantly delayed tumour growth compared with either 0.2% cellulose (*p* < 0.0001 and = 0.0344) or psyllium (*p* < 0.001 and = 0.0417; Fig. 2c, d). To assess the specific radiosensitising effects of the high-fibre diets (i.e. removing the effect of diet alone in delaying tumour growth), we aligned day 0 to the day of tumours reaching 80–100 mm^3^ or receiving IR, and compared the tumour growth curves among different dietary groups (Supplementary Figure S6a). Psyllium plus RS significantly radiosensitised bladder tumours compared to 0.2% cellulose (*p* = 0.0047) and psyllium (*p* = 0.0005). This result showed that psyllium plus RS had a greater effect than psyllium plus inulin in mediating tumour control by ionising radiation and the benefit seen with psyllium plus inulin was a composite effect of high dietary fibre influencing tumour growth *per se* and subsequent IR (Figs. 1a and 2c). Patient responses to anti-cancer treatments can vary widely, and as a result, they are categorised based on their responses. This stratification helps identify potential biomarkers and sensitisers for personalised treatment approaches. To assign responders and non-responders to IR, we split the mice into two clear groups for each diet at a tumour volume of approximately 400 mm^3^ at day 26 post-IR. There were three responding mice who received 0.2% cellulose, five mice in the psyllium, eight mice in the psyllium plus RS and four mice in the psyllium plus inulin groups (Supplementary Figure S6b). We conducted further survival analysis by using the time to quadruple tumour volume as an endpoint (*p* < 0.0001; Supplementary Figure S7a). Irradiated mice in all dietary groups had significantly longer median times for tumours to quadruple in volume compared to non-IR controls and the p-values were 0.0211 for 0.2% cellulose, 0.0337 for psyllium, 0.0004 for psyllium plus RS and 0.0039 for psyllium plus inulin (Supplementary Figure S7b). These data reflect 6 Gy conferring some degree of survival advantage and demonstrate a greater impact of irradiation in the group fed psyllium plus RS compared to psyllium plus inulin.

The gut microbiota profile shows that a *Muribaculaceae* uncultured bacterium, *Bacteroides* species and *Bifidobacterium animalis* were the three bacteria with the largest abundance among all the other bacteria taxa (Supplementary Figure S8). These taxa were consistent with the result of the previous cohort in Fig. 1c. To explore how specific bacterial taxa affect tumour control in the psyllium plus inulin group, we investigated the correlation between Lachnospiraceae family abundance versus tumour growth rate because this was the top enriched taxon for responders (Supplementary Figure S9a and S9b). In this diet group, tumour growth post-tumour inoculation was negatively correlated with Lachnospiraceae family relative abundance in the IR cohort (*R*^2^ = 5344, *p* = 0.0163; Fig. 2e) but this was not seen in the non-IR cohort (*R*^2^ = 0.1557, *p* = 0.5109; Supplementary Figure S9c). Principal coordinate analysis of faecal microbiota in the responders and non-responders to IR in the psyllium plus RS (*R*^2^ = 0.1296, Pr(>F) = 0.11) and psyllium plus inulin (*R*^2^ = 0.1157, Pr(>F) = 0.339) groups is shown in Supplementary Figure S10. The *Bacteroides* genus comprised around 14 to 20% of the gut microbiota in mice in the psyllium plus RS group (Supplementary Figure S11a). Its abundance was higher in the IR cohort, and it was significantly associated with better tumour response to irradiation (*R*^2^ = 0.6114, *p* = 0.0075), in contrast to the non-IR cohort (*R*^2^ = 0.5460, *p* = 0.1037; Fig. 2f, Supplementary Figure S11a). *Parasuterella* (*R*^2^ = 0.8016, *p* = 0.0400; Supplementary Figure S11d) and *Faecalibaculum* (*R*^2^ = 0.8462, *p* = 0.0269; Supplementary Figure S11e) genera, enriched in the mice fed with psyllium plus RS (Supplementary Figure S2a and S2b), were associated with better tumour control in the non-IR cohort of this diet group but not the IR cohort. Additionally, our result confirmed that dietary fibre was a more statistically significant factor affecting sample distances compared to cages (see Supplementary Table S1 and Supplementary Figures S12, S13 and S14 for the corresponding *p* values).

### Enhanced immune response and metabolite production by psyllium plus inulin or psyllium plus RS combined with IR in bladder tumours

**Fig. 3:**
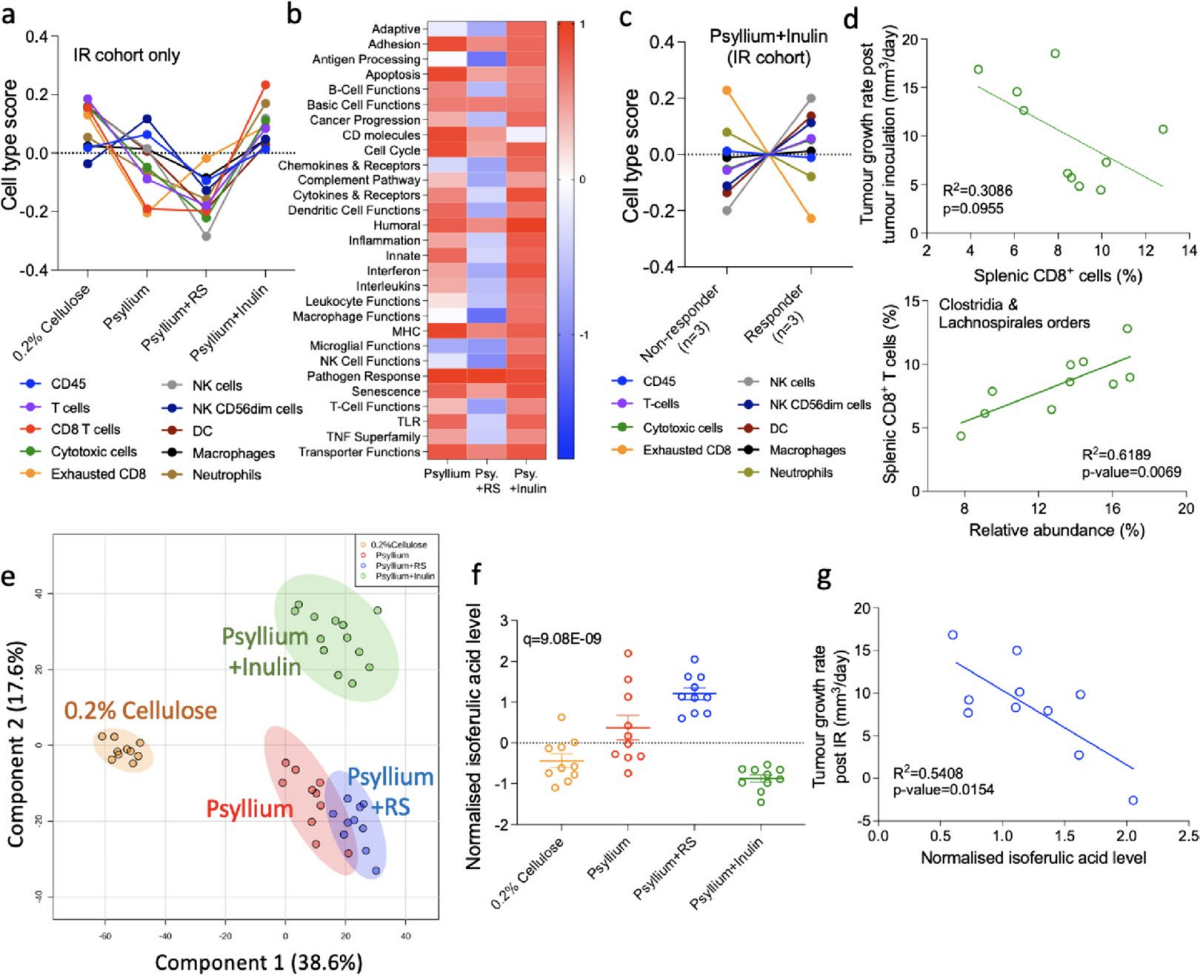
Modulation of local immune responses and caecal metabolites profile by psyllium plus either inulin or RS. **a** Immune cell profiling and **b** pathways of NanoString platform were used to study the local tumour immunity (*n* = 6/group). **c** Immune cell profiling in responders and non-responders to IR in psyllium plus inulin groups (*n* = 3/group). **d** Correlation of splenic cytotoxic T cells versus tumour growth rates in the IR cohort of psyllium plus inulin group. Correlations between the Clostridia and Lachnospirales orders versus the tumour growth rate and population of splenic cytotoxic T cells in the IR cohort of the psyllium plus inulin group. **c**, **d** The associations were assessed using Pearson’s correlation method. **e** Principal component analysis of caecal metabolites of different dietary groups. A notable clustering effect by diets was seen in the metabolites. ADONIS test was used to confirm the existence of significant differences among different dietary groups in terms of metabolite profiles. **f** Caecal isoferulic acid levels normalised by the median in all dietary groups. ANOVA test, followed by post-hoc analysis using Fisher’s LSD and ***p*** value adjustment using the Benjamin-Hochberg method, was used to assess the significance of differences among each dietary group. **g** Correlation between the caecal isoferulic acid level and tumour growth rate in the IR cohort of the psyllium plus RS was assessed using Pearson’s correlation method. Data are presented as mean ± SEM

NanoString analysis provided an overview of the immune cell profile and immune-related gene enrichment (a set of genes whose expression is over-represented) in tumours. Consistent with the intra-tumoural immune responses in Fig. 1f, a trend for increased CD8^+^ cells was found in the psyllium plus inulin group compared to the psyllium plus RS group after irradiation (*p* = 0.15; Fig. 3a, Supplementary Figure S15a and S15b). In addition, there was also a trend for elevated levels of the other immune cells, including neutrophils (*p* = 0.0648) and NK cells (*p* = 0.0905), in the psyllium plus inulin group compared to the RS group (Supplementary Figure S16a). Immune responses were enriched in tumours of the psyllium plus inulin group compared to psyllium plus RS, especially the pathways of humoral immunity, cytokines and their receptors, and interferon, along with *Bst1* and *Nfatc2* gene expression (Fig. 3b, Supplementary Figure S16b). The immune-related genes with higher expression levels in psyllium plus RS were *Tgfb3*, *Nrp1*, *Fn1* and *Ada* (Supplementary Figure S16b). Since local tumour immune response was enhanced by psyllium plus inulin, we investigated whether this local tumour and/or systemic immunity was associated with tumour response. In tumours, the number of exhausted CD8 cells significantly decreased in responders (*p* = 0.0433; Fig. 3c and Supplementary Figure S17a). In addition, the T cell functional pathways, and cytokines and receptors were enriched in responders to psyllium plus inulin (Supplementary Figure S17b). For T cell function, the significantly up-regulated genes which had an adjusted *p* value of < 0.01 were *Bcl10*, *Il2ra*, *Il18rap*, *Ca*rd*11*, *Cd5*, *Tnf*, *Dpp4*, *Il12b* and *Fasl*. For cytokines and receptors in responders, there were seven genes significantly up regulated, namely, *Traf3*, Il18r*ap*, *Card11*, *F2rl*, *Il1*2b, *Tnf* and *Fasl*, while the *Il1r2* gene was down regulated. This implies that the up-regulation of genes related to T cell function, and also cytokines and relevant receptors may be needed for the tumour response to IR and psyllium plus inulin. In terms of systemic immune responses, for the effector cells, there was a trend for tumour growth rate negatively correlating with splenic cytotoxic T cells (*R*^2^ = 0.3086, *p* = 0.0955, Fig. 3d top panel). GM-CSF and IL-2 were two cytokines belonging to the Th1 cytokine panel which have been used to improve the efficacy of radiotherapy by activations of immune cells and enhancement of antigen presentations [31]. We observed a negative correlation between GM-CSF and tumour growth (*R*^2^ = 0.2864, *p* = 0.0486) and a non-significant trend for IL-2 (*R*^2^ = 0.2288, *p* = 0.0713; Supplementary Figure S17c). To explore whether the gut microbiota modulates the systemic immune response, we assessed the correlation between Clostridia and Lachnospirales (bacterial taxa associated with tumour control) abundance versus systemic immune cells. There were significant correlations between the abundance of these orders and splenic cytotoxic T cells (*R*^2^ = 0.6139, *p* = 0.0069; Fig. 3d bottom panel) and macrophages (*R*^2^ = 0.4609, *p* = 0.0309; Supplementary Figure S18b). This implies that the dietary fibre-modified gut microbiota might be required to activate systemic immune responses.

Discovery metabolomics was performed to compare the caecal metabolome of mice fed with different dietary fibres. Principal components analysis (PCA) showed a notable clustering effect of metabolite profiles by diet (*R*^2^ = 0.5166, Pr(>F) = 0.001; Fig. 3e). Consistent with the gut microbiota profile in Fig. 1e, the psyllium and psyllium plus RS groups were more similar to each other compared to the other dietary groups. An ANOVA analysis comparing the metabolite levels showed that compared to the 0.2% cellulose dietary group, the psyllium plus RS group had a significantly higher level of isoferulic acid (*q* = 9.08E−09; Fig. 3f), a metabolite previously shown to inhibit human leukemic cell growth [32]. Isoferulic acid was also associated with better tumour control in the psyllium plus RS group (*R*^2^ = 0.5408, *p* = 0.0154; Fig. 3g). The unfavourable metabolites (positively correlated with tumour growth) were related to amino acid metabolism, namely, glycine, serine, threonine, cysteine, and methionine (Supplementary Figure S19a and S19b). In the IR cohort of psyllium plus inulin, threitol was associated with improved tumour control (*R*^2^ = 0.5595, *p* = 0.0125; Supplementary Figure S20a). The asparaginyl-hydroxyproline level was also associated with a slower tumour growth rate in the IR cohort and non-IR cohort in the psyllium plus inulin group (*R*^2^ = 0.4952, *p* = 0.0073; Supplementary Figure S20b).

### Dietary fibre manipulation spares IR-induced normal tissue toxicities

**Fig. 4:**
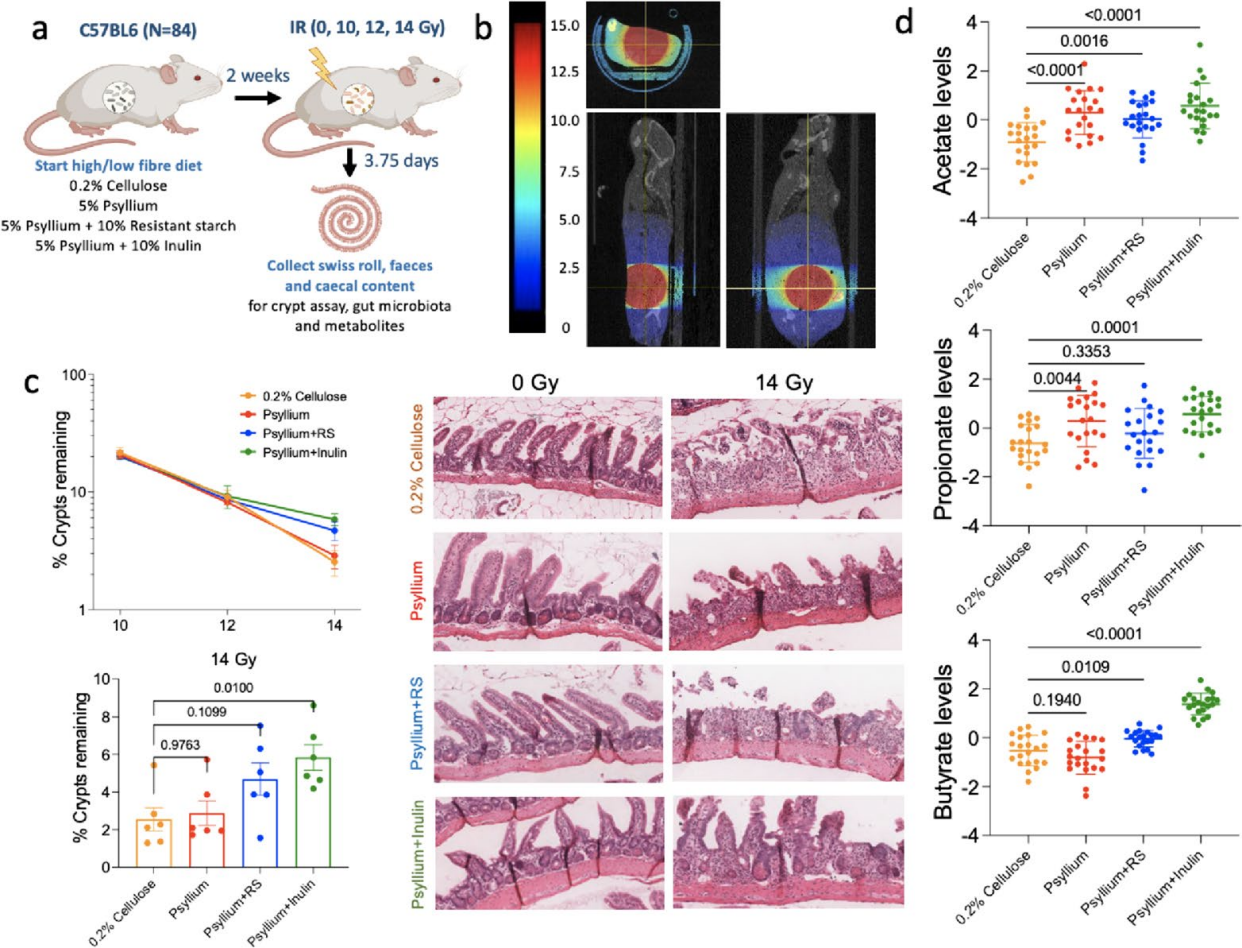
Psyllium plus inulin mitigated the radiation injury from 14 Gy in intestinal crypt assays. **a**, **b** Overview of acute normal tissue toxicity experiment. Two weeks after starting low or high-fibre diets, C57BL/6 mice were treated supine with 10–14 Gy SARRP IR to their lower abdomen (*n* = 21/group). Tissues were collected 3.75 days after IR to assess the acute normal tissue responses. Retrieved from https://app.biorender.com/biorender-templates. **c** Small intestinal crypt assay survival for modified diets and IR (*n* = 6 per group, except for 0 Gy IR: *n* = 3). Data were normalised to mean crypts per mm of three mock samples. Kruskal-Wallis test and Dunn’s multiple comparison tests were used to compare the number of remaining crypts among the dietary groups. **d** Caecal SCFAs in non-tumour-bearing mice after a 3-week modified diet. One-way ANOVA with Bonferroni’s multiple comparison test was used to compare the means of different groups. Data are presented as mean ± SEM

While aiming to improve tumour control is key, it is also advantageous in terms of the therapeutic ratio to reduce normal tissue toxicity if possible. As there is evidence of the benefits of SCFA and dietary fibre in relieving radiation-induced symptoms [33, 34], we investigated whether dietary fibre supplementation could spare acute intestinal toxicity caused by radiation (Fig. 4a). After feeding the mice with modified diets for 2 weeks, we irradiated their lower abdomen, centred on the urinary bladder, and covering part of the small and large intestines, with 10, 12 and 14 Gy on the Xstrahl small animal radiation research platform (SARRP) (Fig. 4b). We applied our modified crypt assay to investigate whether there was a protective effect conferred by the dietary fibre. There was no difference among the different dietary groups when the mice received 10 and 12 Gy IR, but psyllium plus inulin (*p* = 0.0100) increased the number of crypts remaining in mice receiving the higher 14 Gy dose of IR, while psyllium plus RS showed a non-significant trend (*p* = 0.1099; Fig. 4c). Gut microbiota profiles showed a similar composition within the same dietary groups before and after irradiation (Supplementary Figure S21) and, in terms of beta diversity (a measure of similarity between communities), PCoA showed a notable cluster effect among the gut microbiota for all psyllium diets groups (*R*^2^ = 0.3616, Pr(>F) = 0.001; Supplementary Figure 22a). In mice receiving different doses of SARRP IR within the same dietary group, we found that the higher the dose of IR, the larger the distance of the gut microbiota from the non-IR (0 Gy) controls (Supplementary Figure S22b). All radiation doses significantly changed the gut microbiota in 0.2% cellulose (*p* = 0.0183, 0.0037 and < 0.0001 for 10, 12 and 14 Gy), while for psyllium plus RS (*p* = 0.0124 for 12 Gy) or psyllium plus inulin (*p* = 0.0109 and 0.0005 for 12 and 14 Gy) only 12 Gy and 14 Gy caused significant changes. However, it is noted that the observed significance may be influenced by uneven sampling of controls and other groups, as well as interindividual variability (Supplementary Figure S22a and b). One of the major physiological functions of the gut microbiota is the secretion of metabolites. The PCA of the discovery metabolomics analysis showed a clustering effect within each dietary group (*R*^2^ = 0.6747, Pr(>F) = 0.001; Supplementary Figure S22c). Notably, psyllium and psyllium plus RS exhibited more similar metabolomic profiles compared to psyllium plus inulin (see Supplementary Figure 22c). In addition, we saw a significant clustering effect in metabolite profiles among non-IR and IR cohorts only in the 0.2% cellulose group (*R*^2^ = 0.2383, Pr(>F) = 0.031 for 0.2% cellulose), while the psyllium group showed a non-significant trend (*R*^2^ = 0.2259, Pr(>F) = 0.061 for psyllium; *R*^2^ = 0.1343, Pr(>F) = 0.570 for psyllium plus RS; *R*^2^ = 0.1832, Pr(>F) = 0.302 for psyllium plus inulin; Supplementary Figure S22d). All psyllium-containing diets significantly increased the caecal SCFA levels, but not isovaleric acid and isobutyrate, after 3 weeks of the modified diet (Fig. 4d, Supplementary Figure S23a). Psyllium significantly raised the acetate (*p* < 0.0001) and propionate (*p* = 0.0044) levels, and psyllium plus RS increased acetate (*p* = 0.0016) and butyrate (*p* = 0.0109) levels. Psyllium plus inulin resulted in the highest levels of acetate, propionate and butyrate among all dietary groups and the *p* values were < 0.001 for all three SCFAs compared to 0.2% cellulose.

Patients may develop long-term side effects from 3 months after receiving pelvic radiotherapy. To investigate whether dietary fibre and the gut microbiota can protect the intestine from late radiation-induced injury, we performed an experiment irradiating the mice with 5 × 5 Gy IR to the lower abdomen, including their urinary bladder and the lower part of the large intestine, avoiding the small intestine by treating the mice head down (Supplementary Figure S24a and b). The mice were then followed up for 24 weeks. Consistent with the acute toxicity experiment, mice fed psyllium plus RS and psyllium plus inulin had higher butyrate levels compared to 0.2% cellulose-fed mice after 9 weeks of the modified diet (Supplementary Figure S23b). However, we found no significant fibrosis in the colons at 20 weeks post-irradiation in any group (Supplementary Figure S25). Commensurate with this, mice generally maintained or gained weight on the diets apart from a brief weight loss in 0.2% cellulose-fed mice during radiotherapy (Supplementary Figure S26 and S27). Five weeks after changing back to normal chow from the modified diets, minor clustering effects for all diet groups were still found (*R*^2^ = 0.5018, Pr(>F) = 0.001 for unweighted UniFrac and *R*^2^ = 0.1591; Pr(>F) = 0.014 for weighted UniFrac; Supplementary Figure S28 and S29).

### Bacterial supernatants of co-cultures of B. acidifaciens and a butyrate-producing bacterium induced greater cytotoxic response, histone deacetylase inhibition, IR-induced DNA damage and radiosensitivity

**Fig. 5:**
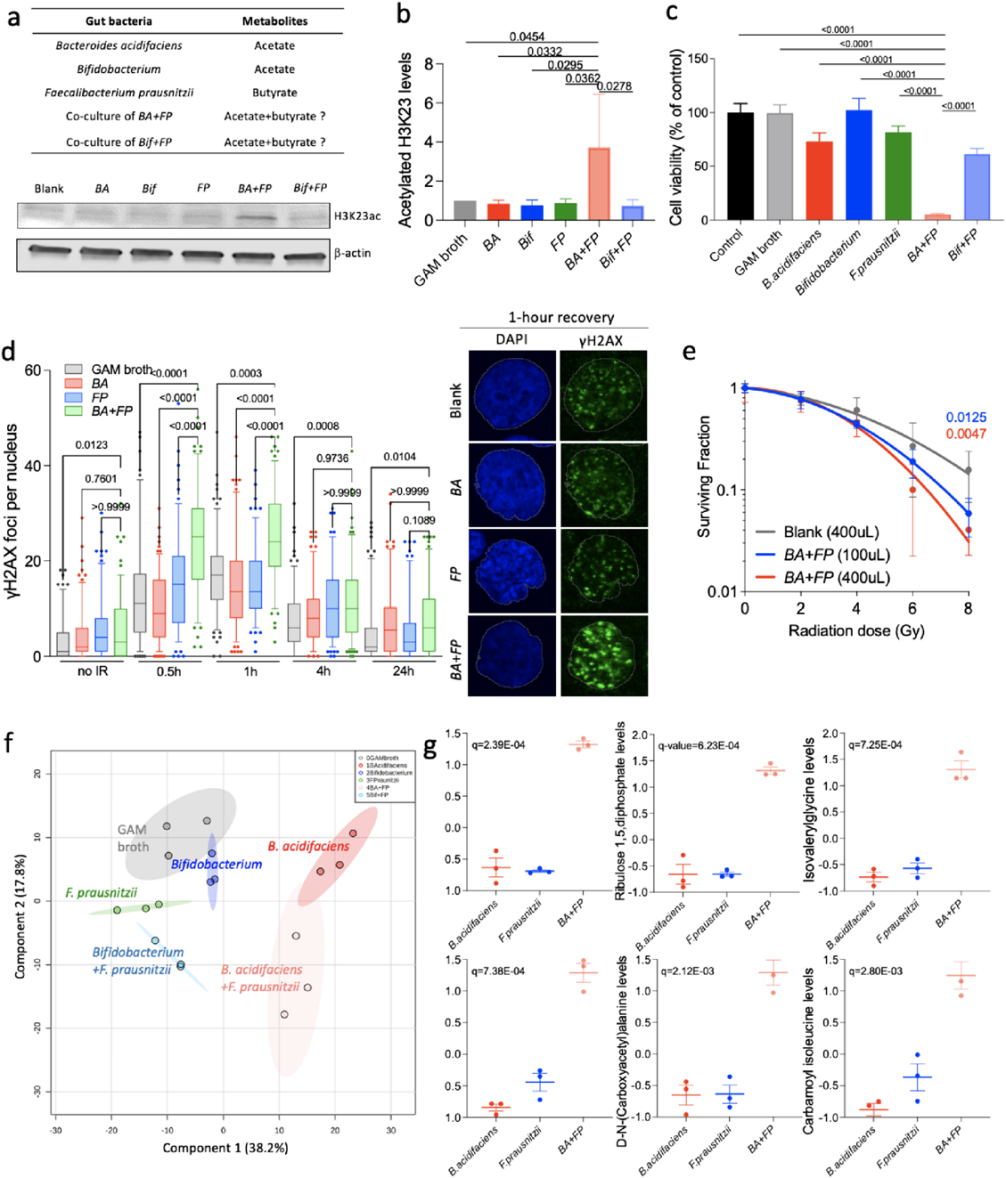
Bacterial supernatants from the cocultures of *B. acidifaciens* and *F. prausnitzii* conferred stronger anti-tumour phenotypes in bladder cancer cells. **a** The five bacterial supernatants used in this experiment. **b** Western blot analysis of histone acetylation (*N* = 3) of RT112 cells treated with different bacterial supernatants. Histone acetylation levels were determined after treating with 100 mL bacterial supernatants in 2 mL of medium for 24 h. **c** The cell survival of RT112 cells treated with 200 mL of GAM broth or bacterial supernatants in 500 mL of medium for 2 days (*N* = 3). **d** Immunofluorescence microscopy analysis of γ-H2AX levels (*N* = 3) in RT112 cells treated with 100 mL bacterial supernatants in 2 mL of medium for 24 h. DNA damage was evaluated after treating with 2 Gy IR. Kruskal-Wallis test and Dunn’s multiple comparison tests were conducted to compare the means of different groups. **e** Linear quadratic survival curves of RT112 cells treated with 100 or 400 μL bacterial supernatant from *BA+FP* for 24 h before receiving irradiation of 0–8 Gy (*N* = 3). **b**, **c** and **e** One-way ANOVA with Bonferroni’s multiple comparison test was used to compare the means of different dietary groups. **f** Principal component analysis of known metabolites of different bacterial supernatants. The clustering effect was assessed using the ADONIS test (*R*^2^ = 0.6495, Pr(>F) = 0.001). **g** Relative levels of metabolites enriched in the bacterial supernatant of *BA+FP*. ANOVA test, followed by post-hoc analysis using Fisher’s LSD and *p* value adjustment using the Benjamin-Hochberg method, was applied to identify the metabolites with significantly different levels across the groups. pHs of GAM broth and bacterial supernatants were all neutralised to 7.2. *BA+FP* denotes the co-culture of *B. acidifaciens* and *F. prausnitzii*, while *Bif+FP* denotes the co-culture of *Bifidobacterium* and *F. prausnitzii.* Data are presented as mean ± SD

We further performed a series of *in vitro* experiments to study the influence of the gut microbiota on cancer cells through the production of metabolites. In our previous study, *B. acidifaciens* was enriched in responders to irradiation, and we proposed this *Bacteroides* species might be a potential radiosensitiser [26]. In this current study, we also found that the *Bacteroides* genus was associated with tumour control by psyllium plus RS. So, we used bacterial supernatants to investigate the anti-tumoural properties of *Bacteroides acidifaciens* as a model organism for *Bacteroides* spp. (Fig. 5a). There was very limited knowledge about this bacterium although it produces acetate [35], required for butyrate production. Therefore, we compared the cellular effects of *B. acidifaciens* to the well-known acetate producer, *Bifidobacterium animalis* (*Bif*). Given that HDAC inhibition is a promising mechanism of radiosensitisation [36], the results showed that bacterial supernatants from the co-culture of *B. acidifaciens* (*BA*) plus *F. prausnitzii* (*FP*) significantly increased histone acetylation (Fig. 5b) compared to the control and the other supernatants. The p-values were as follows: 0.0454 for GAM broth, 0.0032 for *BA*, 0.0295 for *Bif*, 0.0362 for *FP* and 0.0278 for *Bif*+*FP*. The cytotoxic response of RT112 (Fig. 5c) and T24 (Supplementary Figure S30a) human bladder cancer cell lines significantly increased in *BA*+*FP* compared to the other supernatants including *B. acidifaciens* alone, *B. animalis* (acetate-producer), F*. prausnitzii* (butyrate-producer) and the co-culture of *Bif+FP*. In all cases, the p-values were < 0.0001 for both cell lines. Similarly, treating RT112 and T24 cells with *BA+FP* supernatant demonstrated a greater delay in repair of γH2AX nuclear foci by immunofluorescence microscopy than for each of the individual supernatants (please refer to Fig. 5d and Supplementary Figure S30b for the corresponding *p* values). Also, 4 h after 5 Gy IR, there were significantly higher levels of DNA damage in *BA+FP* compared to the other supernatants (*p* = 0.0301 for GAM broth, 0.0138 for *Bif*, 0.0384 for *FP*), except *BA* (*p* = 0.0512) and *Bif+FP* (*p* = 0.0845), as measured by γH2AX protein levels (Supplementary Figure S31). We further studied the radiosensitising properties of bacterial supernatants of *BA+FP* by irradiating the bladder cancer cells from 0 to 8 Gy (Fig. 5e). Clonogenic assay showed that the supernatant can radiosensitise RT112 bladder cancer cells in a dose-dependent manner (*p* = 0.0125 for 100 μL and *p* = 0.0047 for 400 μL). The metabolite profile by bacteria showed that ADP (*q* = 2.39E−04), ribulose 1,5-diphosphate (*q* = 6.23E−04), isovalerylglycine (*q* = 7.25E−04), butyrate (*q* = 7.38E−04), D-N-(carboxyacetyl) alanine (*q* = 2.12E−03) and carbamoyl isoleucine (*q* = 2.80E−03) were enriched in the supernatants of *BA+FP*, that had significantly higher cytotoxic responses, compared to *B. acidifaciens* or *F. prausnitzii* alone (Fig. 5f, g).

### Comparison of microbial profiles between human and mouse faecal samples

**Fig. 6:**
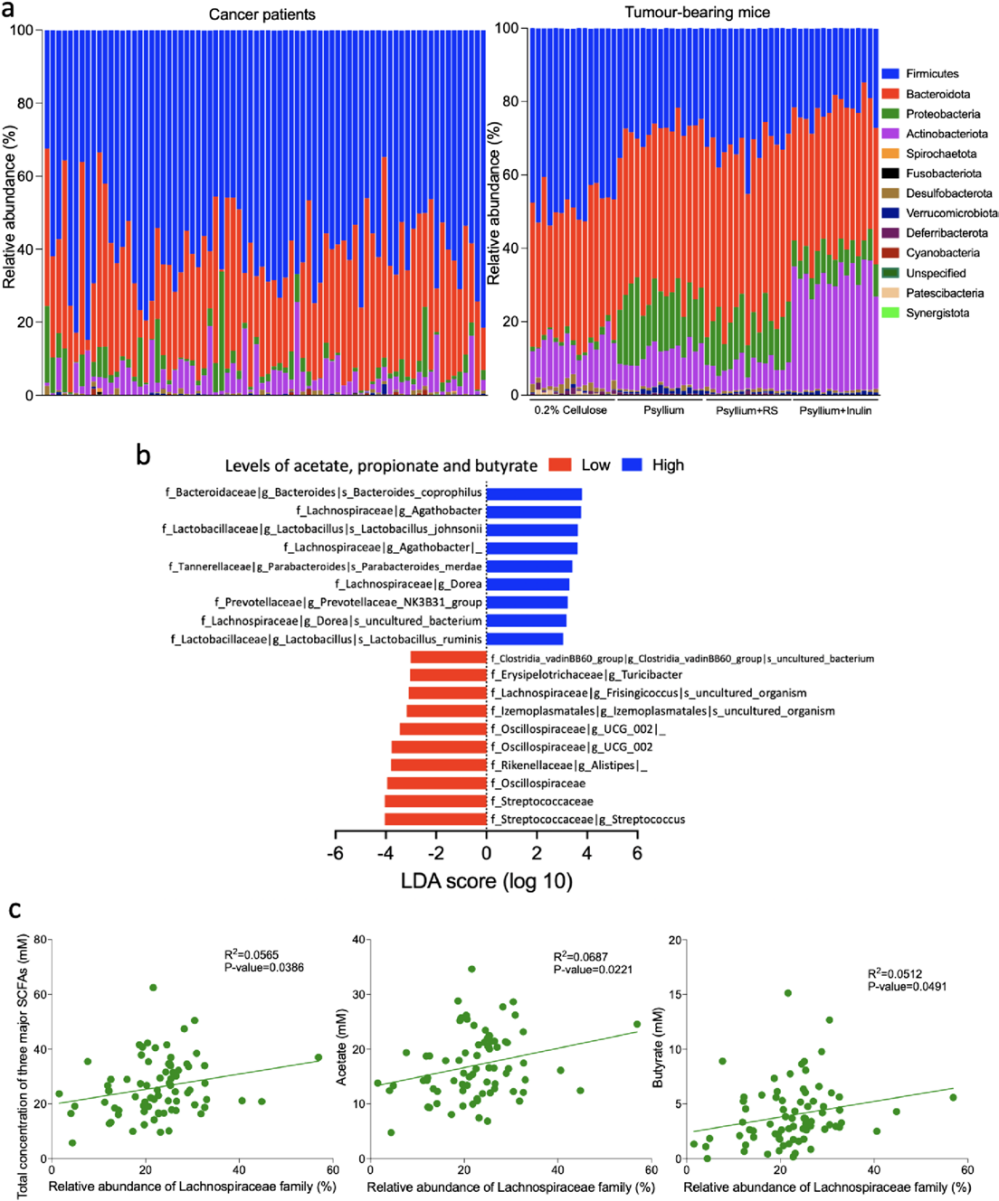
Comparison of gut microbiota from pelvic cancer patients and tumour-bearing mice treated with a low-fibre diet. **a** Relative abundances of bacteria at the phyla level between cancer patients and mice fed on different diets. **b** Linear discriminant analysis (LDA) scores were computed for differentially abundant taxa in the patients with either high or low faecal acetate, propionate and butyrate concentrations. Median of the three SCFAs combined was the cut-off between high and low levels. The alpha value was 0.05 for the Kruskal-Wallis test and the length of the bar indicates the effect size associated with a taxon. **c** Correlations between Lachnospiraceae family versus the total concentration of three major gut microbiota produced SCFAs, acetate and butyrate were assessed using Pearson’s correlation method

We aimed to investigate the gut microbiota composition in cancer patients and explore whether specific gut bacteria colonising the human gut were linked to short-chain fatty acid (SCFA) production. These bacteria may represent a promising group of gut microbiota candidates for enhancing beneficial fibre fermentation in future research studies. Analysis of phylum-level gut microbiota composition showed that the cancer patients’ profiles were more similar to those of mice fed a low-fibre diet (0.2% cellulose) than the other high-fibre diet groups, with higher Firmicutes and lower Proteobacteria abundance (Fig. 6a, Supplementary Table S2). SCFA analysis of cancer patient samples demonstrated a broad range of faecal SCFA levels among individuals, especially acetate, propionate, and butyrate (Supplementary Figure S32a). We found that the abundance of several bacterial taxa was significantly different in patients with either high or low faecal acetate, propionate and butyrate by using the median of the three SCFAs combined as cut-off value (Fig. 6b, Supplementary Table S3, Supplementary Figure S32b). We have confirmed that the effect of refrigerated storage time (up to 3 days) on stool samples was minimal. Samples from three individuals processed serially at 24-h intervals (0, 24, 48 and 72 h) had no significant intra-individual differences in microbial composition, diversity or SCFA concentrations (Supplementary Figure S33a–d, Supplementary Table S4). Despite a significant difference in between-individual gut microbiota profiles (*R*^2^ = 0.9039; Pr(>F) = 0.002), among all SCFAs, only butyrate was lower in sample 3 compared to samples 1 (*p* = 0.0022) and 2 (*p* = 0.0128; Supplementary Figure S33d and e).

There were nine bacteria taxa enriched in the high SCFA group, namely, *Bacteroides coprophilus*, *Agathobacter* genus, *Lactobacillus johnsonii*, *Agathobacter* species, *Parabacteroides merdae*, *Dorea* genus, *Prevotellaceae_NK3B31_group* genus, *Dorea* uncultured bacterium species and *Lactobacillus ruminis*. It is noted that four of these belong to Lachnospiraceae, a family that was associated with better tumour control by psyllium plus inulin in mice (Fig. 2e). We also showed that Lachnospiraceae family abundance in cancer patients was positively correlated with the total concentration of three major SCFAs from gut microbiota (I^2^ = 0.0565, *p* = 0.0386), acetate (*R*^2^ = 0.0687, *p* = 0.0221), butyrate (*R*^2^ = 0.0512, *p* = 0.0491) and valerate (*R*^2^ = 0.1024, *p* = 0.0048) concentrations, but not formate *R*^2^ = 0.444, *p* = 0.0675) and propionate (*R*^2^ = 0.127, *p* = 0.3322; Fig. 6c, Supplementary Figure S34). However, it is noted that the low *R*^2^ values might imply that the significant correlation observed could be influenced by outliers. Regarding another favourable gut bacterium that was associated with tumour radiosensitisation by psyllium plus resistant starch (Fig. 2f), there was also a trend of association between *Bacteroides* genus versus faecal formate level (*R*^2^ = 0.0450, *p* = 0.0656, Supplementary Figure S35e).

## Discussion

In our previous study, we used dietary fibre to enhance radiosensitivity in an immunocompromised bladder tumour model and to our knowledge, we have been the first to show that this was associated with the modulation of gut microbiota [26]. Here, in an immunoproficient mouse model, we have shown that psyllium plus inulin slowed tumour growth and significantly reduced intestinal normal tissue toxicity, assessed by intestinal crypt assay, while psyllium plus RS acted as a tumour radiosensitiser with some non-significant improvement in normal tissue toxicity (*p* = 0.1099). In cancer patients receiving radiotherapy, supplementation of several different dietary fibres can mitigate the side effects of radiotherapy. For example, psyllium has been shown to be effective in reducing the incidence and severity of radiation-induced diarrhoea [27]. RS is well-known as a source of butyrate production via bacterial fermentation in the colon [28], and butyrate is the main energy source of colonocytes and is an anti-inflammatory agent. In a large clinical study of patients receiving radiotherapy to their prostate and pelvic lymph nodes, radiotherapy changed the gut microbiota, and this was associated with early and late enteropathy in patients [37]. In a recent systematic review including twenty-three randomised, controlled trials, we also showed that biotic supplements, especially probiotics and synbiotics, reduce acute symptoms of diarrhoea in patients undergoing pelvic radiotherapy [38].

In terms of tumour control, psyllium plus inulin had the largest effect compared to 0.2% cellulose, psyllium, and psyllium plus RS. This result is supported by previous studies where inulin delayed tumour growth of mouse breast cancer [19] and enhanced tumour control combined with irradiation in mouse bladder tumours [25, 26], thereby demonstrating anti-tumour effects of inulin in cancers outside the intestines, and its ability to enhance the efficacy of anti-cancer treatments. We found that psyllium plus inulin increased tumour growth delay, when combined with irradiation, compared to 0.2% cellulose and psyllium. Tumour growth rate is significantly negatively correlated with a relative abundance of the Lachnospiraceae family, belonging to Clostridiales order, which emphasises the importance of gut microbiota modification in enhancing tumour control. A previous study, in mice receiving melanoma immunotherapy, also supports our finding that Clostridiales abundance is associated with the enhancement of systemic CD8^+^ cells [5]. Furthermore, our discovery metabolomics analysis showed that psyllium plus inulin increased the level of caecal inosine as has previously been shown to enhance immunotherapy efficacy via activation of cytotoxic T cells [15, 39].

There are very few studies regarding resistant starch and tumour growth outside colorectal cancer. Some resistant starches (whole wheat bread, legumes and boiled potato) are associated with reduced breast cancer risk [40] while Hi-Maize 260 RS decreased tumour size in a mouse pancreatic cancer model [41]. Mathers et al. also found that 30 g/day RS can reduce the development of extracolonic cancers in Lynch Syndrome patients [42]. In this study, we showed that, although psyllium plus RS had a smaller effect on tumour control compared to psyllium plus inulin, it conferred a higher radiosensitising effect. The IR response in psyllium plus RS negatively correlated with *Bacteroides* genus, suggesting that the IR response was associated with the gut microbiota. From the discovery metabolomic analysis, isoferulic acid was significantly enriched in mice fed with psyllium plus RS and was associated with better tumour control in this diet group. Ferulic acid, an isomer of isoferulic acid, has been shown to suppress homologous recombination-dependent repair in breast cancer cells [43] and even confer a radiosensitising effect in lung [44], liver [44] and cervical [45] cancer cells. It is reported that, compared to ferulic acid, isoferulic acid had a higher inhibitory potency on a murine macrophage cell lin e[46]. The suppression of the immune response with a higher expression of the immunosuppressive cytokine gene *Tgfb3* in psyllium plus RS again suggests that its radiosensitising effect might be achieved via non-immune mechanisms.

A limitation of this study is that we had initially expected psyllium alone to have an effect on tumour growth delay and crypt regeneration, which would have allowed us to observe any additional effects of inulin or resistant starch. As this was not the case, we cannot draw any conclusions on whether the dietary fibre combinations were required or whether inulin and/or resistant starch alone would suffice, although this is worthy of further study. The subcutaneous xenograft model was used as it allows the effects of dietary fibre on the systemic components of the immune system and circulating metabolites to be studied. While an orthotopic bladder tumour model was considered, its potential to more accurately reflect the tumour microenvironment was outweighed by the heterogeneity of tumour growth, technical difficulty of accurately measuring intravesical tumours and the more complex radiotherapy delivery procedures that would be required, all of which would introduce undesirable experimental variability.

Consistent with the association between the *Bacteroides* genus and tumour control post-IR by psyllium plus RS, *Bacteroides acidifaciens* was identified as a potential radiosensitiser in our previous study, where its abundance was positively correlated with tumour response to irradiation [26]. Studies suggest that a broad range of gut microbiota-derived metabolites can enhance anti-tumoural effects or tumour response to anti-cancer treatments [47]. Beyond SCFAs, future work should include the identification of radiosensitisers with similar profiles from the global metabolomic analysis of *B. acidifaciens*. In addition, greater cytotoxic phenotypes (Fig. 5b–d) generated by co-culture of *BA* and butyrate-producing bacteria also suggests the need to build a collection of combinations of bacteria, including *BA* and other butyrate producers, to pursue a better response than achieved with a single bacterium alone. This was shown in a previous study of a community of 11 strains, primarily rare, low-abundance components of the human microbiota, which enhanced the therapeutic efficacy of immunotherapy in a mouse colorectal cancer model [48].

Spencer et al. also showed that high dietary fibre increased the efficacy of melanoma immunotherapy in both mouse and human [22]. This phenomenon was associated with the enhancement of T cell function, also seen in this study [22]. However, this effect was diminished when combining the dietary fibre with probiotics – *Lactobacillus* or *Bifidobacterium*. This implies that dominance of these probiotic species may have overridden the gut microbiota that could have enhanced the efficacy of anti-cancer treatments. This evidence supports dietary fibre as a better approach to enhance tumour control rather than dosing with a small number of specific bacteria, because it can shape the whole gut microbiota to a more favourable profile, with more diverse metabolites produced. In addition, Hu *et al.* showed that beta-glucan (Maitake) mitigated chemoradiation-related adverse effects in head and neck cancer patients in a randomised clinical trial [49].

In this study, mice were inoculated with cancer cells on the same day their normal chow was replaced with the modified diets, allowing time for gut microbial colonisation prior to ionising radiation. In clinical practice, patient radiotherapy preparations, including computed tomography simulation, delineation, and dose calculation, usually require up to 1 month. Therefore, commencing a modified diet is not only practical from a clinical perspective but also allows ample time for the modification of gut microbiota and the alteration of metabolite profiles.

We did not see any impact of dietary modifications on intestinal fibrosis as late normal tissue toxicity, but this is likely due to the radiation dose of 25 Gy in 5 fractions [36] being too low (as evidenced by no fibrosis on the 0.2% cellulose diet). With hindsight, it would have been more informative to deliver 35 Gy in 5 fractions, near the top of the late toxicity dose-response curve [50], which would have allowed us to ascertain whether the dietary interventions ameliorated the fibrosis.

There is no available guideline to translate or convert dietary fibre intake from mouse study to human trial due to the complexity of considering fibre intake per body weight, metabolic differences and digestibility of fibre. It was estimated that mouse experiments typically involve the supplementation of dietary fibre at levels ranging from 5 to 20% weight/weight, equivalent to a minimum of 20 to 80 grams per day for humans [51]. Five percent psyllium [52], 10% resistant starch [53], or 10% inulin [54] alone are common quantities used to investigate the effects of dietary fibre. Notably, psyllium has been examined in a concentration of up to 15% in studies exploring its protective role in mouse colitis experiment s[55]. Psyllium is commercially available in products containing 3.5 g per sachet (Fybogel; Ispaghula Husk), reinforcing their suitability for human consumption. Clinical trials have used up to 10.5 g/day of psyllium [56] and 16 g/day of inulin [57], but there is a lack of literature exploring their maximum dosages. Previous human studies have also employed doses of up to 40 g per day of butyrylated high-amylose maize starch [58] or 40 g per day of non-starch polysaccharides [59]. It is noted that the Scientific Advisory Committee on Nutrition (SACN) recommends that humans should be aiming for over 30 g/day of dietary fibre intake [60]. However, elevated consumption of dietary fibre could lead to unpleasant gastrointestinal side effects including abdominal discomfort, bloating, and diarrhoea [61]. Recently, Gunn et al. showed that 20 g of psyllium can reduce gas production caused by 20 g of inulin in a human MRI study whilst maintaining fermentation, found by *in vitro* testing [62]. This suggests that adding psyllium together with inulin and RS should be well tolerated in human patients, although further studies are needed.

A limitation of the study is that it lacks detailed elucidation of the mechanistic roles of the diet and gut microbiota. We therefore propose several approaches to investigate the molecular mechanisms through which dietary constituents impact the gut microbiota and the subsequent response of tumours and the immune system to ionising radiation. For example, combining high dietary fibre intake with faecal microbiota transplantation from responders and non-responders will illuminate how diet supports a beneficial microbiota. Another strategy is to administer, via oral gavage, the putative radiosensitising gut bacteria to germ-free mice or to antibiotic-treated specific pathogen-free mice. It is crucial to consider the specific bacteria within the context of bacterial consortia as it is likely that a beneficial microbiota community is required [48]. Anti-CD8 depletion antibodies could be used to further analyse the anti-tumour immune response associated with different diets in combination with radiation. Moreover, studies have shown that an enhanced anti-tumour immune response is associated with a rise in gut antigen-presenting cells [63] and increased expression of MHC class I in colonic dendritic cells [48]. Therefore, employing a dendritic cell depletion mouse model (such as a refined XCR1-DTR-Venus transgenic mouse model) could illuminate the importance of this cell type in defining the outcome of radiotherapy plus dietary fibre [64]. Regarding discovery metabolomics, examining specific metabolites in samples other than faeces, including serum and tumours, should also provide further mechanistic insights.

In the pelvic cancer patients, we found that many had a similar microbiota profile at the phylum level to tumour-bearing mice fed the low-fibre diet (0.2% cellulose). Based on our findings in mice, this suggests that there may be scope to target the human gut microbiota with dietary fibre supplementation in many cancer patients. The variability observed in the baseline microbiota profiles in cancer patients suggests that we may be able to determine responders and non-responders to fibre supplementation in future clinical trials.

In conclusion, we demonstrated that dietary fibre supplements impacted the radiation response of bladder tumours and surrounding normal tissues, with associated gut microbiota modification and enhancement of immune responses and/or metabolite levels. We propose that dietary fibre supplements may be useful adjuncts to radiotherapy in patients with pelvic malignancy. This approach has the potential to improve patient outcomes at a low cost and minimal distress to patients.

## Methods

### Mice

All animal experiments complied with UK Home Office Guidelines, following the ARRIVE (Animal Research: Reporting of In Vivo Experiments) guidelines. We obtained approval from the University of Oxford Animal Welfare and Ethical Review Body (AWERB), under University of Oxford project licences (PPLs) P8484EDAE and PP8415318. We used a G-Power program [65] to choose group sizes for detecting large effect sizes. C57BL/6J mice were all purchased from Charles Rivers (UK). All mice were housed in a temperature-controlled environment with a 12-h reversed-phase light/dark cycle (lights on 07:00 h) and provided with food and water *ad libitum* at the Department of Biomedical Services, Radiobiology Research Institute, University of Oxford, Oxford. The normal chow global diet 2918 contained 12.0% moisture, 18.5% crude protein, 5.5% crude oils and fats, 4.5% crude fibres and 6% crude ash (Mucedola, Italy). All diets were given to mice in the form of pellets. We randomised the mice by using the RAND function in Excel. Mice receiving the same dietary fibre were housed in the same cages but were randomised to either undergo irradiation or not. Randomisation for irradiation was performed after tumour inoculation for tumour growth experiments or when they were started on a modified diet for normal tissue toxicity experiments.

### Allograft model, modified diets and irradiation method

At five to 6 weeks of age, C57BL/6 female mice were injected subcutaneously under anaesthesia with 1.5 × 10^6^ UPPL1591 mouse bladder cancer cells with Hanks’ Balanced Salt Solution (HBSS; Lonza) and phenol red-free Matrigel (BD Biosciences) mixture with a ratio of 1:1 at a total volume of 200 μL in the right flank. Meanwhile, they started receiving either normal chow, low-fibre diet (0.2% cellulose) or high-fibre diets including 5% psyllium, 5% psyllium plus 10% RS or 5% psyllium plus 10% inulin for a maximum time of 9 weeks or until they were euthanised when the tumour reached 100 mm^3^ or 700 mm^3^. All diets are isocaloric, approximately 4 kcal/g, and details of the diet formulae are listed in Supplementary Table S5 (Research Diets Inc., USA). Psyllium, RS, and inulin formulations were psyllium husk powder (AEP Colloids), Hi-Maize 260 RS (Ingredion) and Orafti HP inulin (Beneo), respectively. The murine bladder cancer cell line UPPL1591 was created by Dr. Ryoichi Saito and was maintained in DMEM, high glucose, GlutaMAX supplement, pyruvate (Gibco) medium supplemented with 10% fetal bovine serum (Gibco). Tumour growth was measured three times a week using callipers and calculated using the following formula for the volume of an ellipsoid [66]: Length × width × height × π/6, given that π (pi) is the mathematical constant that is approximately equal to 3.14159. These tumour measurements were not conducted in a blinded manner but the individual performing the measurements remained unaware of the irradiation status. When the tumours reached 100 mm^3^, flank allografts were treated prone with 6 Gy of X-rays using a Gulmay-320 cabinet irradiator (300 kV, Xstrahl Inc., UK).

### Non-tumour-bearing mice, modified diets and irradiation method

At six to 7 weeks of age, C57BL/6 female mice started receiving either a low-fibre (0.2% cellulose) or high-fibre diets including 5% psyllium, 5% psyllium plus 10% RS or 5% psyllium plus 10% inulin for a maximum time of 9 weeks followed by normal chow for another 12 weeks. All diets are isocaloric, approximately 4 kcal/g, and details of the diet formulae are listed in Supplementary Table S5 (Research Diets Inc, USA). For acute toxicity experiment, two and a half weeks after commencing the modified diets, mice were treated supine with 10, 12 or 14 Gy of X-rays (220 kVp, 13.0 mA copper filtered beam with a measured half-value layer (HVL) of 0.84 mmCu) to the lower abdomen, including the lower small intestine using a SARRP irradiator (Xstrahl Ltd, Camberley, UK). For late toxicity experiments, 2 weeks after commencing the modified diets, mice were treated supine using a SARRP with 5 Gy for 5 consecutive days, using a 356° arc treatment and 8.5-mm collimator, with the isocentre positioned at the posterior caudal bladder wall covering the lower large intestine, to avoid the small intestine. In both experiments, small and large intestines were collected using the ‘Swiss roll technique’ described in (Moolenbeek and Ruitenberg, 1981) [67].

### Microbiome sample collection and DNA extraction from mice

Mouse faeces were snap-frozen in dry ice once they were collected from mouse intestines under aseptic conditions. All samples were kept at − 80 °C before DNA extraction. Bacterial genomic DNA was extracted using a DNeasy PowerSoil Pro DNA Isolation Kit (QIAGEN Ltd., Manchester, UK), as described previously [26] and the Human Microbiota Project [68]. All DNA samples were kept at – 80 °C before being sent for library preparation and sequencing at the Oxford Genomics Centre (Wellcome Centre for Human Genetics, University of Oxford, UK).

### Bacterial 16S rRNA gene sequencing in mice

16S rRNA gene sequencing methods were adapted from the methods developed for the NIH-Human Microbiota Project [68]. The amplification and sequencing of 16S rRNA gene V3V4 regions were done on a MiSeq platform (Illumina, Inc., San Diego, CA, USA) using their 2 × 300 bp paired-end protocol, yielding paired-end reads with near-complete overlap. The primers (S-D-Bact-0341-b-S-17/S-D-Bact-0785-a-A-21) [69] containing adapters for Miseq sequencing were used for amplification and single-end barcodes, allowing pooling and direct sequencing of PCR products [70].

Raw sequence data was analysed using the QIIME2 platform, LEfSe and R packages as described previously [26]. All 16S rRNA gene-based metagenomic analyses were conducted using a QIIME2 platform [71]. Sequencing errors were de-noised by using the “Deblur” plugin [72]. After paired-end reads were merged to form consensus sequences, sequences were trimmed to a length of 300. In the taxonomic analysis, we classified the microbiota at the phylum, class, order, family, genus, and species levels by referring to the SILVA 138 database [73]. In some cases, ‘|_’ means that the classifier was unable to assign taxonomy at this level. The LEfSe method of analysis was performed to compare the abundances of all bacterial clades between groups [74]. The effect size was obtained by LDA (linear discriminant analysis) using the Kruskal-Wallis test at the α setting of 0.05. A phylogenetic tree was generated by using the “phylogeny” plugin from QIIME2 and the diversity commands of “alpha-group-significance” and “beta-group-significance” were used to obtain Shannon’s index. A principal coordinate (PCoA) plot was obtained by using the Emperor Tool based on the results of the Bray-Curtis dissimilarities, unweighted or weighted UniFrac distances [75]. ADONIS test was used to assess whether there are statistically significant differences in microbial community composition between different dietary groups.

### Discovery metabolomics analysis

Caecal or faecal contents were added to four-fold Millipore Synergy purified water at a ratio of 1:4 (caecal content:water) for homogenisation and were sent to the Department of Chemistry (University of Oxford, UK) on dry ice for discovery metabolomics analysis using ion chromatography-mass spectrometry (IC-MS). Supernatant was filtered using an Amicon ultra-0.5 centrifugal filter Unit (Merck, Cat. No. UFC500396) at 14,000×*g* for 25 min at 4 °C and collected in total recovery vials (Waters Corporation). For the ‘allograft model, modified diets and irradiation’ and the bacterial supernatant experiments, samples were analysed as described previousl y[76], but the scan range was changed to 50–750 m/z. Analytes were separated with an aqueous hydroxide ion gradient at a flow rate of 0.25 mL/min with the following steps: 0 min, 0 mM; 1 min, 0 mM; 15 min, 60 mM; 25 min, 100 mM; 30 min, 100 mM; 30.1 min, 0 mM; 37 min, 0 mM. For the ‘non-tumour-bearing mice, modified diets and irradiation’ experiments, analytes were separated with an aqueous hydroxide ion gradient at a flow rate of 0.25 mL/min with the following steps: 0 min, 0 mM; 1 min, 0 mM; 17 min, 40 mM; 20 min, 100 mM; 22.1 min, 0 mM; 25 min 0 mM. The presence of butyric acid was confirmed by retention time and accurate mass comparison with an authentic standard (Merck, Cat. No. CRM46975). Data processing was performed using Progenesis QI for small molecules (Waters Corp, Elstree, UK) and Metaboanalyst5. 0[77]. In brief, the peak intensities table was uploaded in .csv format. Data normalisation was performed by median in addition to log_10_ transformation and auto-scaling of the data prior to multivariate statistical analysis being performed. In the tables, metabolites tagged with ‘Accepted ID’ are based on comparison with an in-house database and therefore higher confidence because includes fragmentation pattern as a parameter. Metabolites tagged with ‘Putative ID’ are identified based on the accurate mass in Human Metabolome Database (HMDB).

#### Immunohistochemistry

Sections were deparaffinised and hydrated followed by antigen retrieval in pH 9.0 Tris/EDTA buffer using a microwave. The sections were incubated with 3% H_2_O_2_, avidin/biotin blocking kit (SP-2001), and 2.5% normal horse serum blocking solution (MP-7401; Vector laboratories). Subsequently, sections were incubated with CD8 (CST 98941; 1:400 dilution) primary antibody overnight at 4°C. ImmPRESS (Peroxidase) Polymer Anti-Rabbit IgG Reagent (MP-7401; Vector laboratories) was used as secondary antibody. The sections were visualised by DAB staining and counterstained with haematoxylin. The slides were mounted using DPX mounting medium after dehydration. Slides were digitally scanned using the Aperio ScanScope (Leica Biosystems). Cell number quantification of three parts of the tumour core for each tissue was performed on QuPath. The density of CD8^+^ cells within each region of interest could thus be calculated by dividing the positive-stained cell numbers by the analysed area.

#### NanoString

Total RNA was extracted from three 5 μm sections of formalin-fixed paraffin-embedded (FFPE) tissue samples using a RNeasy FFPE kit (Qiagen, 160012457). After RNA extraction, RNA was quantified using a NanoDrop spectrophotometer (Thermo Scientific, San Jose, CA, USA). Nucleic acid fragmentation was measured by an Agilent RNA 6000 Nano Kit on an Agilent 2100 Bioanalyzer System. After the hybridisation of targets, capture probes and reporter probes was completed, the cartridge was analysed at the Nuffield Department of Surgical Sciences, Oxford. Data were analysed using the nCounter mouse PanCancer Immune Profiling Panel, and data acquired with the nCounter SPRINT profiler (NanoString). Data were imported into nSolverTM analysis software v2.5 for quality control and normalisation of gene transcripts using NanoString standard analysis workflow with housekeeping genes.

The nCounter mouse PanCancer Immune Profiling Panel measures the expression of 770 genes categorised based on their function. This includes the identification of immune cells, assessment of immunological functions, and the normalisation of samples using housekeeping genes. NanoString has selected 109 genes to define 24 different immune cell types and populations. These selections were made based on a thorough literature review of studies that investigated the expression of genes in purified population s[78]. The expression levels from these identified cell type-associated gene signatures produce numerical cell scores that reflect proportionally to the abundance of that cell type. In addition, all genes associated with immune responses were used to annotate biological processes, such as B-cell and T-cell functions, cytokines, and cytotoxicity. A comprehensive list of genes included in this panel and their annotated functions can be found on the following website: https://nanostring.com/products/ncounter-assays-panels/oncology/pancancer-immune-profiling/.

#### Flow cytometry

Mouse spleens were kept in PBS on ice and processed to obtain single cell suspensions within two hours of harvest for further flow cytometry analysis, as described previousl y[79]. On the day of flow cytometry analysis, the cells were stained with two panels of antibody mixtures. The myeloid panel included CD45, CD11b, CD11c, Ly6G, F4/8b and Gr-1. The lymphoid panel included CD45, CD3, CD8, CD49b, CD19 and CD4. Cell surface markers of immune cells are listed in Supplementary Table S6, and details of antibodies used are listed in Supplementary Table S7. The samples were run on a LSR II Flow Cytometer (Becton Dickinson) at the Jenner Institute, University of Oxford and analysed on Flow-Jo (Becton Dickinson).

#### Plasma CD4^+^ T-helper cell cytokine assay

Blood was withdrawn from mice under terminal anaesthesia by cardiac puncture using a needle rinsed with heparin and transported on ice for centrifugation. Plasma samples were then stored at -80°C before downstream analysis. Cytokine and chemokine concentrations in the supernatant (pg/mL) were measured using MILLIPLEX mouse 16-plex (IL-1β, IL-2, IL-4, IL-5, IL-6, IL-10, IL-12p70, IL-13, IL-17A, IL-21, IL-22, IL-23, IL-27, GM-CSF, IFNγ, TNFα), IL-18 single plex and TGFβ1 single plex magnetic bead kits (Milli-pore, MA). The plate was prepared as per the protocol described i n[80] and was read on a Luminex 200 instrument (Thermo Fisher) at the Centre for Clinical Vaccinology and Tropical Medicine (CCVTM), Oxford.

#### Crypt assay

Swiss rolls were made from small (three consecutive sections) and large (one section) intestines, and a modified crypt assay as described previously [36] was applied to quantify the acute crypt damage after ionising radiation in a blinded manner. Briefly, regenerating crypts (presence of >10 cells arranged in a distinct shape with no sign of apoptosis) were blind counted. The control number of crypts per length of small intestine was determined from the mean of three mock-treated mice. The percentage of surviving crypts in each group was calculated by dividing number of regenerating crypts per mm by number of control crypts per mm.

#### Histopathological examination

Five μm-thick, formalin-fixed, paraffin-embedded (FFPE) sections from large intestine, arranged as ‘Swiss rolls’ were stained with haematoxylin & eosin (HE) and examined by two board-certified Veterinary Pathologists (ASB and SLP). Sections were histopathologically assessed and graded for the presence of inflammatory changes using a previously described scoring syste m[81] and the International Harmonization of Nomenclature and Diagnostic Criteria for Lesions in Rats and Mice (INHAND) guide for non-proliferative and proliferative lesions of the gastrointestinal tract of the mous e[82]. Histopathological assessment was performed blind to experimental grouping using a conventional light microscope (Olympus BX43). Tissue sections were examined individually by ASB & SLP and in case of discordance in diagnosis a consensus was reached using a double-head microscope. In naive mice, histologically normal intestinal layers contain scant amounts of mature loose connective tissue supporting the lamina propria with minimal fibrous connective tissue in the tunica muscularis and seros a[82]. The presence of fibrosis was assessed qualitatively and grouped in ordinal categories (0, absent; 1, mild; 2, moderate; 3, severe) by identifying expansion of any of the intestinal layers by bands of variably cellular, collagenous-rich connective tissue using H&E and Masson’s Trichome stained tissue sections, as per the criteria of the INHAN D[82, 83].

#### Cell lines and irradiation method

The RT112 human bladder carcinoma cell line was obtained from DSMZ (Germany) and cultured in RPMI-1640 medium (Sigma) supplemented with 10% fetal bovine serum (Invitrogen). The T24 human bladder cancer cell line (ATCC, USA) was cultured in McCoy's 5A medium (Sigma) supplemented with 10% fetal bovine serum (Invitrogen). All cell lines were cultured in a humidified atmosphere of 5% CO_2_ at 37°C and sub-cultured by washing the cells with phosphate buffered saline pH 7.4 (PBS; Gibco) followed by incubation with 0.25% Trypsin-EDTA solution (Gibco) to make new passages at around 90% confluency. Once passaged 10 times, a new batch of cells was thawed and cultured. A stock of cells was kept in -80°C freezer in fetal bovine serum supplemented with 10% dimethyl sulfoxide. All cells used in experiments tested mycoplasma negative. For ionising radiation, cells were irradiated in complete medium at a dose rate of 1.5 Gy/min using a Gamma-Service Medical GmbH GSR D1 irradiator.

#### Bacterial strains and their supernatants

The bacterial supernatants were prepared as described previousl y[26]. All bacterial strains were obtained from DSMZ-German collection of microorganisms. Three strains of bacteria, namely *B. acidifaciens* (*BA*; DSM 15896), *Bifidobacterium animalis* (*Bif*; DSM10140), and *F. prausnitzii* (*FP*; DSM17677), and two cross-feeding combinations (*BA*+*FP* and *Bif*+*FP*) were cultured in Gifu Anaerobic Broth, Modified (GAM; Nissui Pharmaceutical, Japan). The supernatants were neutralised to the same pH as GAM broth (pH 7.2) by adding a minimal volume of 3M NaOH or 3M HCl.

#### Colony formation assay

RT112 cells were seeded at appropriate densities in triplicate, treated with bacterial supernatants for 24 hours, and irradiated with 0, 2, 4, 6, or 8 Gy. After culturing for 10 days, colonies were stained and quantified as described previousl y[26].

#### Cell survival analysis

The MTT 3-(4,5-dimethylthiazol-2-yl)-2,5-diphenyltetrazolium bromide assay was used to assess RT112 cell viability. After the cells were seeded and cultured overnight, they were treated with bacterial supernatants for one to three days. At the end of the experiment, they were incubated in 0.45 mg/mL MTT (Life Technologies) at 37°C for 30 minutes. The absorbance at 595 nm of MTT-formazan was detected spectrophotometrically using an POLARstar Omega Microplate Readers (BMG Labtech). The percentage of cell viability was determined by normalising the absorbance value in each condition to the mock control.

#### Western blotting

Western blot samples were prepared as described previousl y[84]. Protein was visualised using the following antibodies: H3K23Ac (Cell Signaling Technology, #14932), phospho-histone H2A.X (Cell Signaling Technologies, #2577), β-actin (MERCK, #A1978), anti-mouse secondary antibody (LICOR, #925-32210) and anti-rabbit secondary antibody (LICOR, # 925-68021), and imaged using a LI-COR imaging system (Odyssey).

#### Immunofluorescence microscopy and irradiation

RT112 cells were cultured on 10 mm No. 1 cover glasses (VWR) that had been sterilised with 70% ethanol and rinsed with PBS prior to ionising radiation. After incubation with bacterial supernatants and irradiation, cells were allowed to recover for indicated times prior to permeabilisation with 0.3% Triton X-100. Cells were fixed with ice cold 4% paraformaldehyde and blocked by incubation in 5% BSA, as described previousl y[84]. After incubation with primary γH2AX (mouse anti-phospho-Histone H2A.X (Ser139) IgG; clone JBW301, Millipore) and secondary (goat anti-mouse IgG, Alexa Fluor 488, ThermoFisher) antibodies, coverslips were mounted on microscopy slides using mounting reagent, Flouromount G, with DAPI (Invitrogen). Fluorescent foci were imaged using a Zeiss 710 confocal microscopy using either a 40X or 63X objective. All microscopy images were analysed with FIJI (ImageJ) software.

#### Sample collection, DNA extraction and 16S rRNA gene sequencing from cancer patients

Stool samples were collected from 76 pelvic cancer patients (aged 46 – 88 years). The sample collection took place from 01 November 2021 to 25 April 2022. The inclusion criteria for the sample collection included patients undergoing rectal radiotherapy/chemoradiation, right-sided colorectal hemicolectomy, men with prostate cancer undergoing prostatectomy or prostatic biopsy/transurethral resection and endometrial cancer hysterectomy patients in the surgical or oncology departments of Aberdeen Royal Infirmary who consented to take part in the study. The exclusion criteria included approached patients who did not give consent to take part in the study. Antibiotic usage was unknown as we did not have access to patients' notes through the biorepository.

The samples were self-collected by the patients using faecal collection paper and universal faecal collection containers, shortly before an inpatient/outpatient appointment and brought into the hospital or, in the case of 5 samples, obtained at surgery from the right colon. The samples were stored at 4 °C and prepared with no additives as described previousl y[85]. Briefly, the faecal sample (5 g) was weighed and mixed with 10 mL of PBS solution (supplemented with 30% glycerol). An aliquot of 450 μL was stored at -70 °C and used for DNA extraction while a 3 mL aliquot was stored at -25 °C for SCFA analysis. Three of the samples were also processed similarly at 24-h intervals for 3 days to examine the effect of storage on the stability and composition of faecal microbiota and SCFAs.

DNA was extracted from the human faecal samples using the FastDNA Spin kit for soil (MP Biomedicals, UK) according to the manufacturer’s instructions as described previousl y[85]. DNA concentration was quantified by using Nanodrop (Nanodrop One C, Thermo Fisher Scientific, USA). All DNA extracted were stored at -70 °C and 16S rRNA sequencing was carried out at the Centre for Genome-Enabled Biology and Medicine (University of Aberdeen, UK).

Bacterial community profiling of the human faecal samples was performed by sequencing of the 16S rRNA gene V1-V2 region on a MiSeq platform (Illumina, Inc., San Diego, CA) with v3 chemistry and 300bp paired-end reads. Region-specific primer s[69] including partial Illumina adapters were used for amplification of the V1-V2 region, followed by short cycle PCR addition of full-length Illumina adapters and dual barcodes. Resulting libraries were equimolar pooled and sequenced on the MiSeq platform [70]. The raw sequence data were analysed using the DADA 2[86] R package (v 3.6.0) for ASV generation, with subsequent analyses using phyloseq R packag e[87], LEfSe and R packages as described previousl y[26]. In the taxonomic analysis, we classified the microbiota at the phylum, class, order, family, genus, and species levels by referring to the SILVA 138 databas e[73].

#### Short chain fatty acid analysis from human samples

SCFAs from the prepared samples were measured by gas chromatography as described previousl y[88]. Derivatisation of the samples was carried out with N-tert-butyldimethylsilyl-N-methyltrifuoroacetamide (MTBSTFA), and the samples were analysed using a Hewlett Packard (Palo Alto, CA, USA) gas chromatograph. Helium was used as the carrier gas and the gas chromatograph was fitted with a fused silica capillary column. SCFA concentrations were calculated relative to the internal standard two-ethylbutyrate and external standard (a standard mixture of six SCFAs in distilled water).

## Statistical analysis

All statistical analyses were performed on GraphPad Prism version 9.0 (La Jolla, CA) or in R using the VEGAN packag e[89]. All results in *in vivo* studies are mean ± standard error and P < 0.05 is considered statistically significant. A two-tailed Student’s t-test was applied to compare two groups, and one-way ANOVA with Bonferroni’s multiple comparison test was used to compare more than two groups for parametric data. Kruskal-Wallis test and Dunn’s multiple comparisons test were used to compare more than two groups for non-parametric data. Tumour growth curves were analysed for each group, and their slopes were compared using two-way ANOVA. Kaplan-Meier survival analysis was performed to compare the medial survival times using Logrank test. Pearson’s correlation method was used to study association between two parameters or outcomes. In the gut microbiota analysis, alpha diversity was compared using the One-way ANOVA with Bonferroni’s multiple comparison test. ADONIS test was used to confirm the existence of significant group differences in terms of gut microbiota composition. The LEfSe method of analysis was applied to determine the difference in faecal taxa, using the Kruskal-Wallis test. Significantly different taxa presented from the previous comparison were used as input for LDA, which produced an LDA score. PERMANOVA tests with the pseudo-F statistic, as implemented by Vegan, were used to test for significant differences in Bray-Curtis distances between sample groups. In the gut microbiota analysis of human studies, date of processing was found to have a significant effect on Bray-Curtis distance so this was added to the model for all other tests (Supplementary Table S8). No other technical factor (date of DNA extraction, processing time, days before DNA extraction, extraction kit, the person processing, hours from when sample produced) was found to have any effect on sample composition. All analyses were conducted in QIIME2 or R. In the metabolomics analysis and differential immune-related gene expression analysis, ANOVA test, followed by post-hoc analysis using Fisher's LSD and p-value adjustment using the Benjamin-Hochberg (FDR) method, was used to assess the significance of differences among each dietary group. The q-value represents the adjusted p-value derived from the FDR method. In the metabolomics analysis and differential immune-related gene expression analysis, ANOVA test, followed by post-hoc analysis using Fisher's LSD and p-value adjustment using the Benjamin-Hochberg (FDR) method, was used to assess the significance of differences among each dietary group. The q-value represents the adjusted p-value derived from the FDR method. All data in *in vitro* studies are representative of 3 independent biological replicates unless otherwise stated, with results shown as mean and standard deviations. One-way ANOVA with Bonferroni’s multiple comparison test was performed to analyse the data of western blots and MTT assays. Kruskal-Wallis test and Dunn’s multiple comparisons test were used to compare more than two groups for the immunofluorescence experiment. Two-way ANOVA with Bonferroni’s multiple comparison test was used to analyse the linear quadratic survival curves in the colony formation assay.

### Supplementary Information

Additionalfile1:** Supplementary Figure S1**. Differences in bacterial components in the normal chow, 0.2% cellulose and psyllium groups. **Supplementary Figure S2**. Differences in bacterial components in the psyllium, psyllium plus RS and psyllium plus inulin groups. **Supplementary Figure S3**. Local tumour and systemic immune responses in all dietary groups. **Supplementary Figure S4**. Discovery metabolomics analysis of caecal contents in all dietary groups. **Supplementary Figure S5**. Body weight changes of non-IR and IR cohorts of each dietary groups for the mice that did not receive IR or following IR. **Supplementary Figure S6**. Psyllium plus RS radiosensitised UPPL1591 bladder cancer cell allografts. **Supplementary Figure S7**. Survival analysis of tumour-bearing mice without and with IR in different dietary groups. **Supplementary Figure S8**. Phylogenetic composition of faecal microbiota when tumours reached 700 mm3. **Supplementary Figure S9**. Differences in bacterial components in responders and non-responders in the psyllium plus inulin group. **Supplementary Figure S10**. Principal coordinate analysis using Jaccard distance of faecal microbiota in the IR cohorts of (a) psyllium plus inulin or (b) psyllium plus RS. **Supplementary Figure S11**. Correlation between the gut microbiota versus the tumour growth in non-IR and IR cohorts of psyllium plus RS. **Supplementary Figure S12**. Beta diversity of faecal microbiota in the radiosensitisation experiment. **Supplementary Figure S13**. Unweighted UniFrac distance of faecal microbiota between cages in the radiosensitisation experiment. **Supplementary Figure S14**. Weighted UniFrac distance of faecal microbiota between cages in the radiosensitisation experiment. **Supplementary Figure S15**. Local tumour cytotoxic T cells in the IR cohorts of all dietary groups. **Supplementary Figure S16**. Local tumour immune responses in the IR cohorts of all dietary groups. **Supplementary Figure S17**. Local tumour and systemic immunity in psyllium plus inulin stratified by tumour response and IR. **Supplementary Figure S18**. Correlations between the Clostridia and Lachnospirales orders and the populations of splenic (a) leukocytes, (b) macrophages, and (c) natural killer cells in the IR cohorts of psyllium plus inulin group. **Supplementary Figure S19**. Metabolites and KEGG pathway that were associated with tumour growth in mice fed with psyllium plus RS. **Supplementary Figure S20**. Correlations between the caecal (a) threitol, (b) asparaginyl-hydroxyproline and (c) butyrate levels versus the tumour growth rate in IR cohort with or without non-IR cohort in the psyllium plus inulin group. **Supplementary Figure S21**. Phylogenetic composition of faecal microbiota before and after irradiation in the acute toxicity experiment. **Supplementary Figure S22**. Beta diversity of the gut microbiota and the metabolites profile in non-tumour-bearing mice after 3-week modified diet and 3.75 days after SARRP IR. **Supplementary Figure S23**. Caecal SCFAs in non-tumour-bearing mice after 3-week modified diet in acute toxicity and late toxicity experiments. **Supplementary Figure S24**. Overview of late normal tissue toxicity experiment. **Supplementary Figure S25**. Representative images of mouse large intestine sections in non-tumour-bearing mice after 22-week modified diet with or without SARRP IR. **Supplementary Figure S26**. Relative body weight of non-IR and IR cohorts of each dietary groups for the mice that did not receive IR or following IR. **Supplementary Figure S27**. Actual body weight of non-IR and IR cohorts of each dietary groups for the mice that did not receive IR or following IR. **Supplementary Figure S28**. Phylogenetic composition of faecal microbiota before and after irradiation in the late toxicity experiment. **Supplementary Figure S29**. Beta diversity of faecal microbiota in the late toxicity experiment. **Supplementary Figure S30**. BA+FP increased cytotoxic responses and DNA damage in T24 bladder cancer cells. **Supplementary Figure S31**. BA+FP increased histone acetylation levels and DNA damage in bladder cancer cells. **Supplementary Figure S32**. Production of SCFAs in pelvic cancer patients. **Supplementary Figure S33**. Comparison of bacterial and SCFA relative abundances between human samples processed serially showed similar profiles between the different processing times: 0, 24, 48 and 72 hours. **Supplementary Figure S34**. Correlations between Lachnospiraceae family and faecal (a) formate, (b) propionate, (c) valerate levels in cancer patients. **Supplementary Figure S35**. Correlations between Bacteroides genus and faecal (a) total amount of three major SCFAs, (b) acetate, (c) propionate, (d) butyrate, (e) formate, (f) valerate levels in cancer patients. **Supplementary Table S1**. R2 and Pr(>F) values from ADONIS test assessing unweighted and weighted UniFrac distances. **Supplementary Table S2**. Four most abundant phyla for human and mouse samples. **Supplementary Table S3**. Bacteria taxa enriched in cancer patients with low and high faecal SCFAs. **Supplementary Table S4**. Four most abundant phyla of three serial human faecal samples prepared at 24-hour intervals for 72-hours. **Supplementary Table S5**. Rodent diets without corn starch used in the study with varying levels of cellulose, psyllium, psyllium plus resistant starch, or inulin per 4000 kcal. **Supplementary Table S6**. Definitions of immune cell populations based on expression of cell surface markers. **Supplementary Table S7**. Antibody titrations and catalogue numbers. **Supplementary Table S8**. Effect of technical factors on sample composition (Bray-Curtis).

## Data Availability

The datasets generated from mouse samples supporting the conclusions of this article are available in the Figshare repository, https://figshare.com/projects/Dietary_fibre_radiotherapy_and_bladder_tumour/153165. Raw data from the human 16S sequencing are deposited in the SRA, accession: PRJNA935280.
