# Supplementary material for: Dietary fibre supplementation enhances radiotherapy tumour control and alleviates intestinal radiation toxicity
Source: Microbiome. 2024 May 14;12:89. doi: 10.1186/s40168-024-01804-1 (PMC11092108; doi:10.1186/s40168-024-01804-1)
Supplement: Supplementary file 2 — Additionalfile1: Supplementary Figure S1. Differences in bacterial components in the normal chow, 0.2% cellulose and psyllium groups. Supplementary Figure S2. Differences in bacterial components in the psyllium, psyllium plus RS and psyllium plus inulin groups. Supplementary Figure S3. Local tumour and systemic immune responses in all dietary groups. Supplementary Figure S4. Discovery metabolomics analysis of caecal contents in all dietary groups. Supplementary Figure S5. Body weight changes of non-IR and IR cohorts of each dietary groups for the mice that did not receive IR or following IR. Supplementary Figure S6. Psyllium plus RS radiosensitised UPPL1591 bladder cancer cell allografts. Supplementary Figure S7. Survival analysis of tumour-bearing mice without and with IR in different dietary groups. Supplementary Figure S8. Phylogenetic composition of faecal microbiota when tumours reached 700 mm3. Supplementary Figure S9. Differences in bacterial components in responders and non-responders in the psyllium plus inulin group. Supplementary Figure S10. Principal coordinate analysis using Jaccard distance of faecal microbiota in the IR cohorts of (a) psyllium plus inulin or (b) psyllium plus RS. Supplementary Figure S11. Correlation between the gut microbiota versus the tumour growth in non-IR and IR cohorts of psyllium plus RS. Supplementary Figure S12. Beta diversity of faecal microbiota in the radiosensitisation experiment. Supplementary Figure S13. Unweighted UniFrac distance of faecal microbiota between cages in the radiosensitisation experiment. Supplementary Figure S14. Weighted UniFrac distance of faecal microbiota between cages in the radiosensitisation experiment. Supplementary Figure S15. Local tumour cytotoxic T cells in the IR cohorts of all dietary groups. Supplementary Figure S16. Local tumour immune responses in the IR cohorts of all dietary groups. Supplementary Figure S17. Local tumour and systemic immunity in psyllium plus inulin stratified by tumo [file 40168_2024_1804_MOESM1_ESM.pdf]

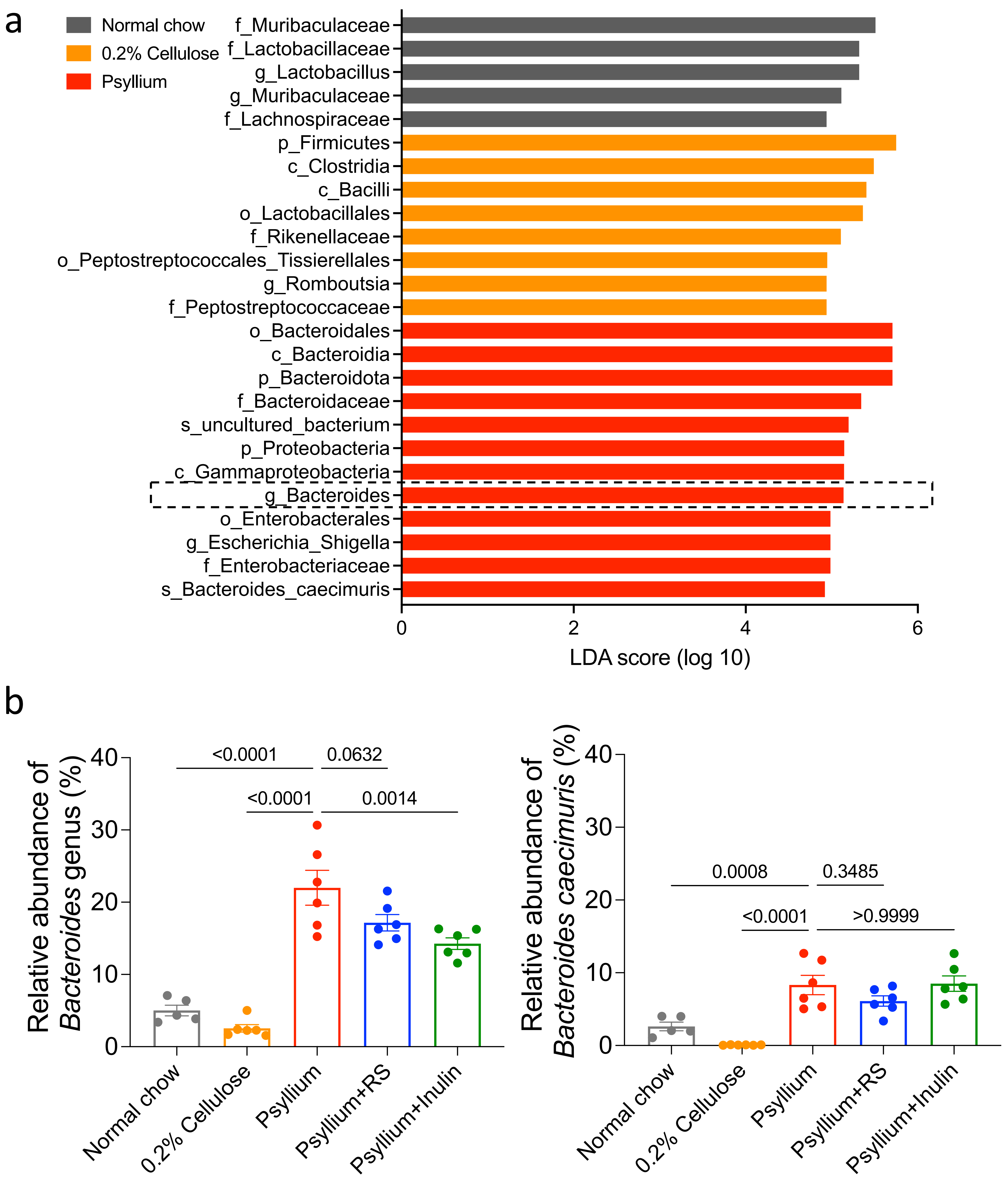

Supplementary Fig. 1 Differences in bacterial components in the normal chow, 0.2% cellulose and psyllium groups. (a) Linear discriminant analysis (LDA) scores computed for differentially abundant taxa in the microbiomes of normal chow (grey), 0.2% cellulose (orange) and psyllium (red). Length indicates the effect size associated with a taxon. (b) Relative abundances of *Bacteroides* genus and *Bacteroides caecimuris* of different dietary groups compared with psyllium by ANOVA with Bonferroni's multiple comparison test.

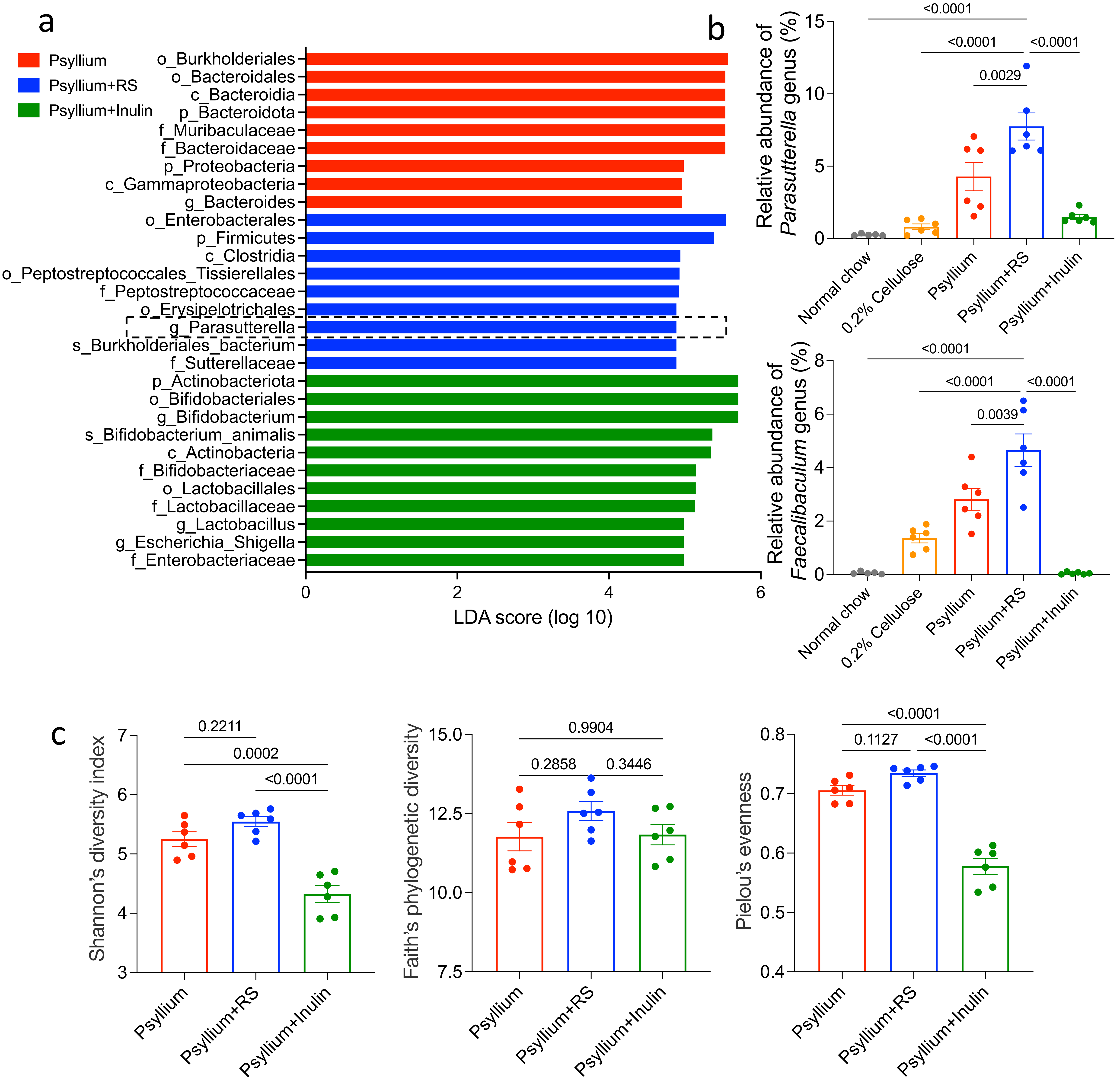

Supplementary Fig. 2 Differences in bacterial components in the psyllium, psyllium plus RS and psyllium plus inulin groups. (a) Linear discriminant analysis (LDA) scores computed for differentially abundant taxa in the microbiomes of psyllium (red), psyllium plus RS (blue) and psyllium plus inulin (green). Length indicates the effect size associated with a taxon. (b) Relative abundances of *Parasutterella* genus and *Faecalibaculum* genus of different dietary groups compared with psyllium plus RS by ANOVA with Bonferroni's multiple comparison test. (c) Alpha diversity using Shannon's diversity index (a measure of community richness), Faith's phylogenetic diversity (a measure of community richness that incorporates phylogenetic difference between species) and Pielou's evenness (a measure of community evenness) of faecal microbiotas. One-way ANOVA with Bonferroni's multiple comparison test was used to compare the means of different dietary groups.

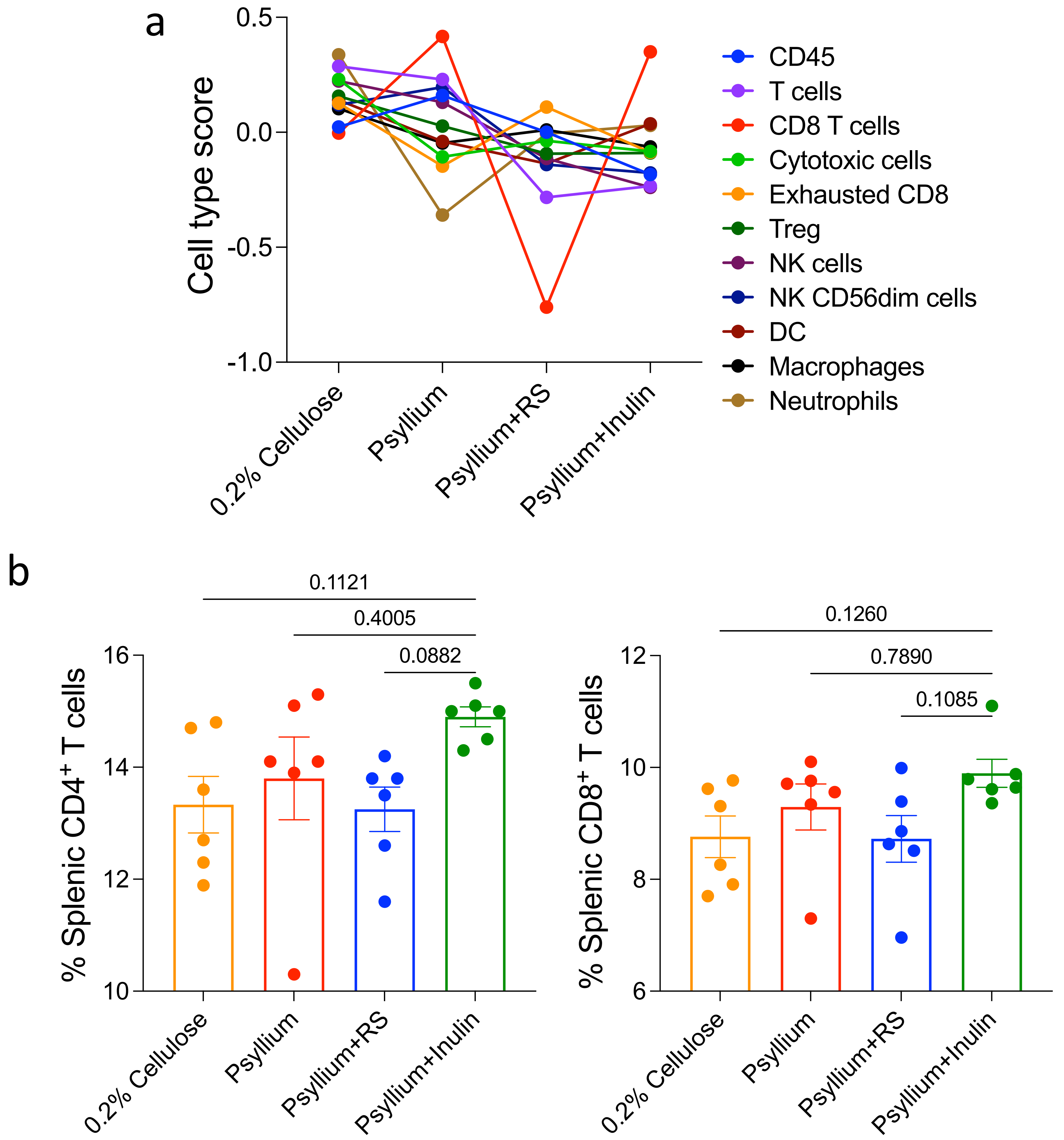

Supplementary Fig. 3 Local tumour and systemic immune responses in all dietary groups. (a) Overall immune cell profiling evaluated on a NanoString platform. (b) Percentages of helper T and cytotoxic T cells measured by flow cytometry analysis. One-way ANOVA with Bonferroni's multiple comparison test was used to compare the means of different dietary groups. Data are presented as mean ± SEM.

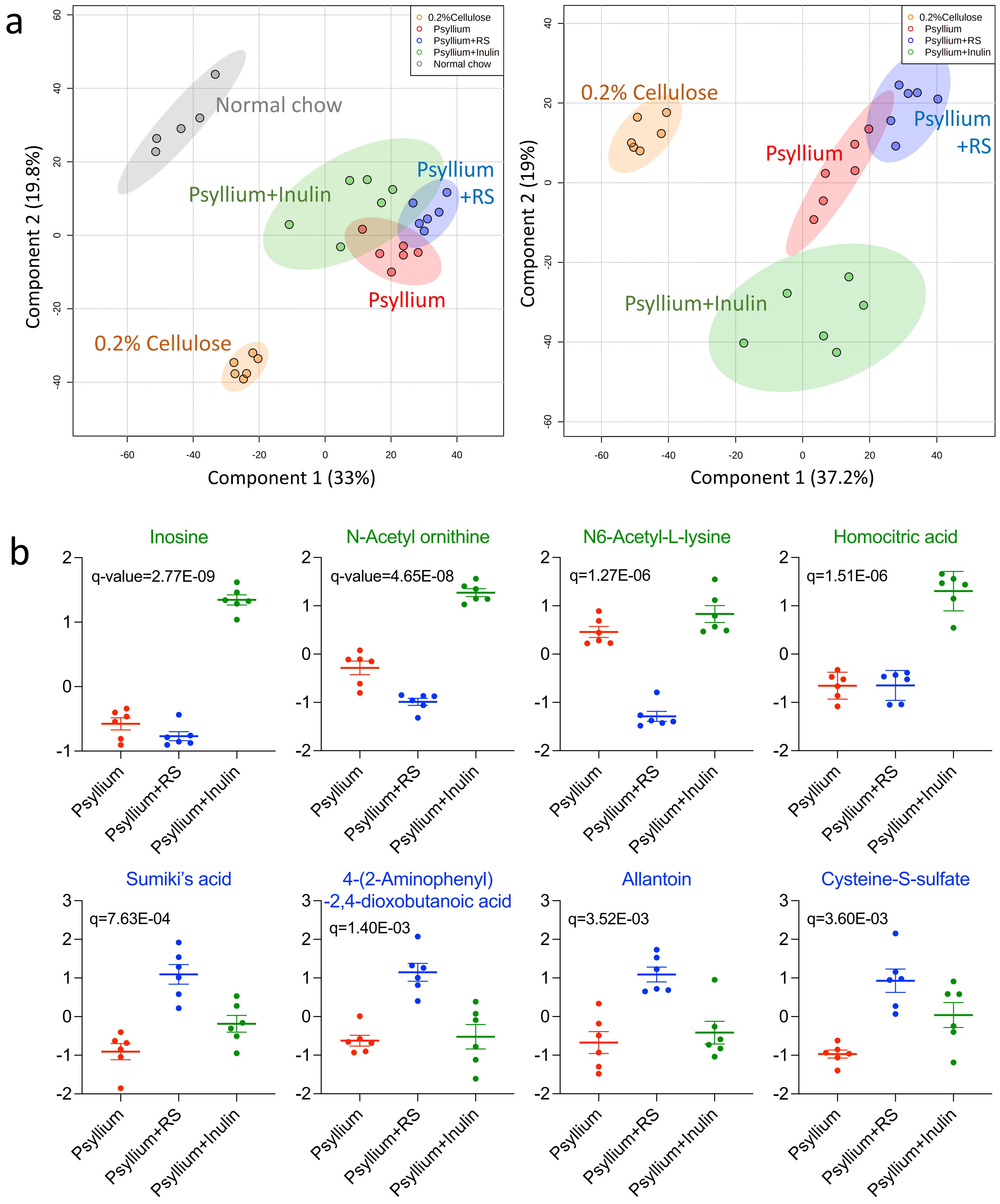

Supplementary Fig. 4 Discovery metabolomics analysis of caecal contents in all dietary groups. (a) Principal component analysis for caecal metabolites of different dietary groups. The left panel included the normal chow group, while the right panel removed it to obtain a better resolution for 0.2% cellulose and all psyllium-containing diets groups. (b) The top four metabolites which had the lowest q-values in ANOVA test, followed by post-hoc analysis using Fisher's LSD and p-value adjustment using the Benjamin-Hochberg method, in each dietary group. Data is mean ± SEM.

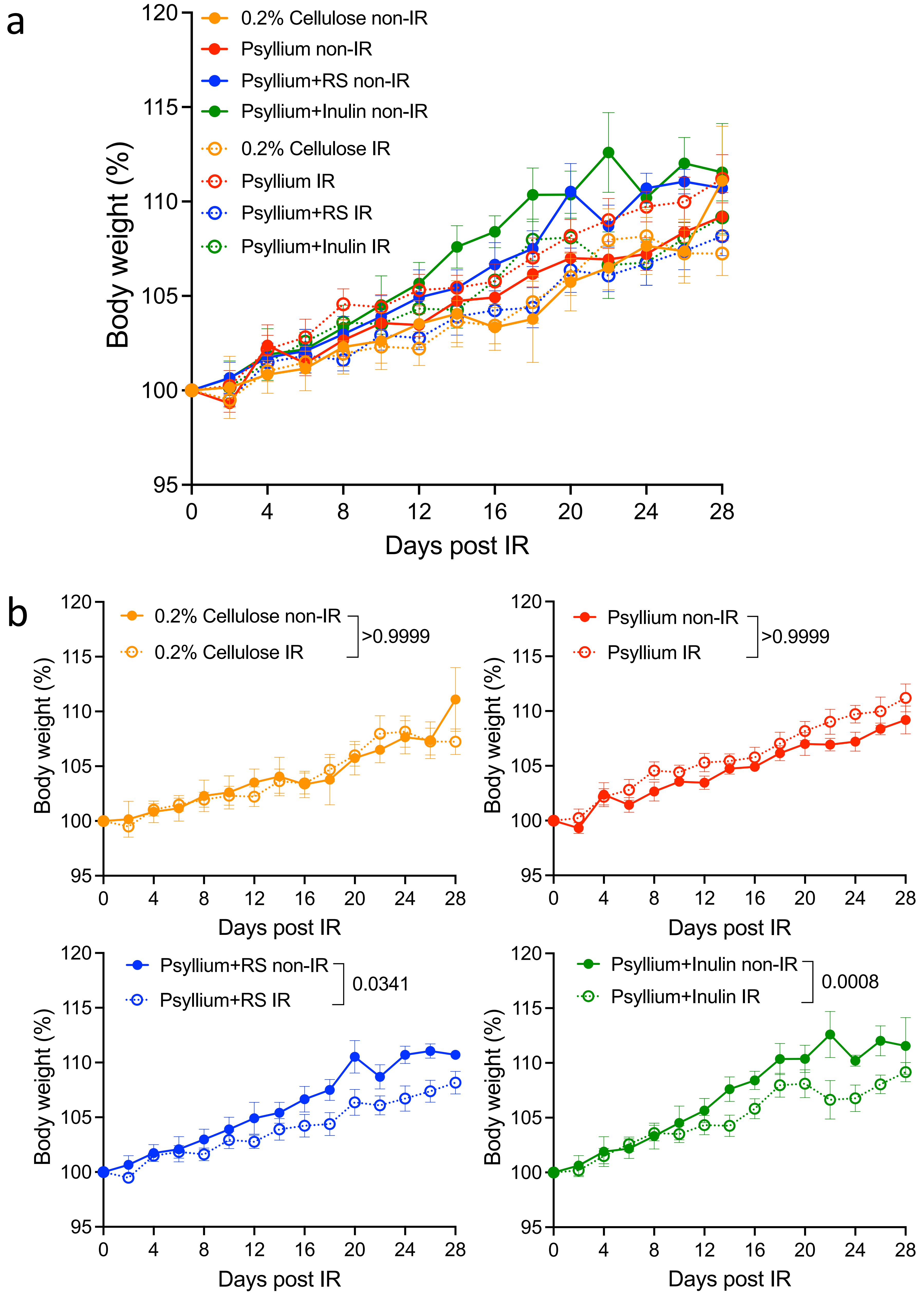

Supplementary Fig. 5 Body weight changes of non-IR and IR cohorts of each dietary groups for the mice that did not receive IR or following IR. Body weight curves of (a) all and (b) single dietary groups were shown. Slopes of body weight curves were calculated by linear regression and compared by ANOVA test and Bonferroni's multiple comparison test. Data are presented as mean ± SEM.

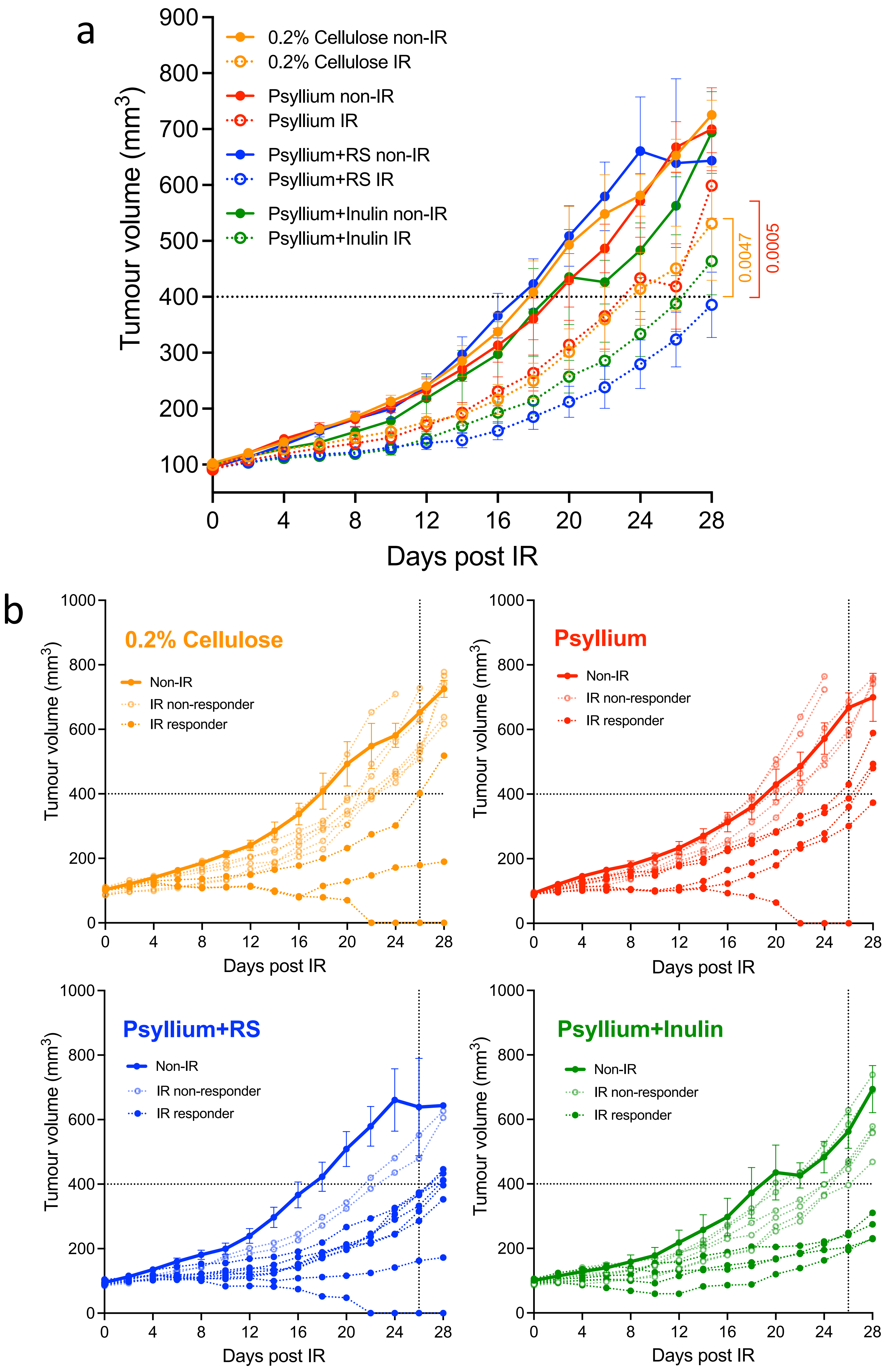

Supplementary Fig. 6 Psyllium plus RS radiosensitised UPPL1591 bladder cancer cell allografts. Treatment of irradiated (6 Gy) UPPL1591 allograft with 0.2% cellulose, psyllium, psyllium plus RS or inulin (n=5 for non-IR and n=10 for IR in each group). (a) The overall growth curves of non-irradiated and irradiated mice were plotted in solid and dotted lines, respectively. In panel (b), solid lines were mean of tumour growth curves of non-IR mice and dotted lines were individual growth curves of IR mice. Day 0 represents the day when tumours reached 80-100 mm^3 and received IR. A criterion for categorising responders and non-responders to IR was based on whether their tumour volumes reached approximately 400mm^3 at day 26 post IR. Data shown as mean and standard error. Slopes of tumour curves were calculated by linear regression to represent tumour growth rates and compared by two-way ANOVA test and Bonferroni's multiple comparison test. Data are presented as mean ± SEM.

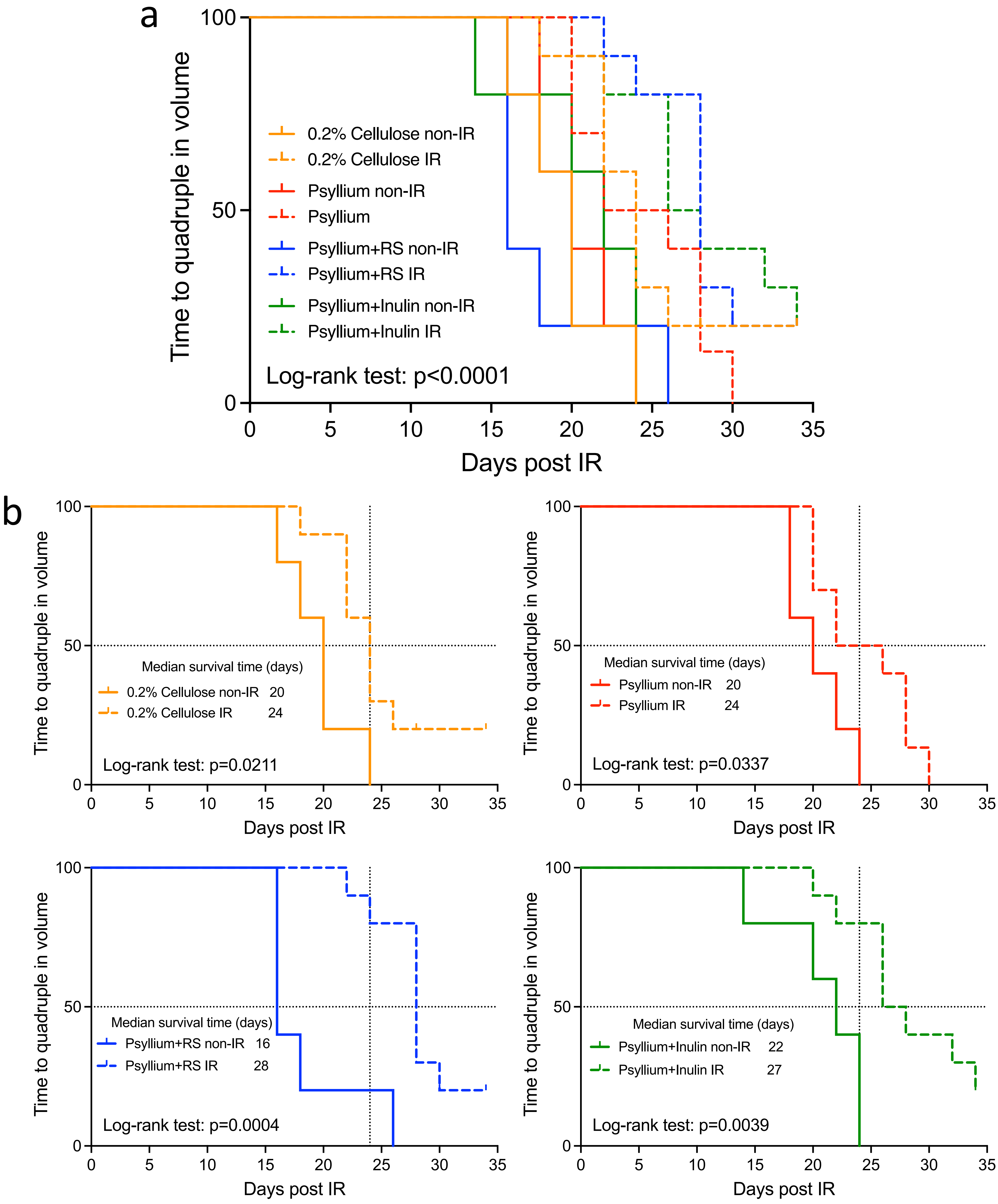

Supplementary Fig. 7 Survival analysis of tumour-bearing mice without and with IR in different dietary groups. (a) Overall and (b) individual Kaplan–Meier survival curve of mice with UPPL1591 allografts showing plots of time to quadruple in tumour volume.

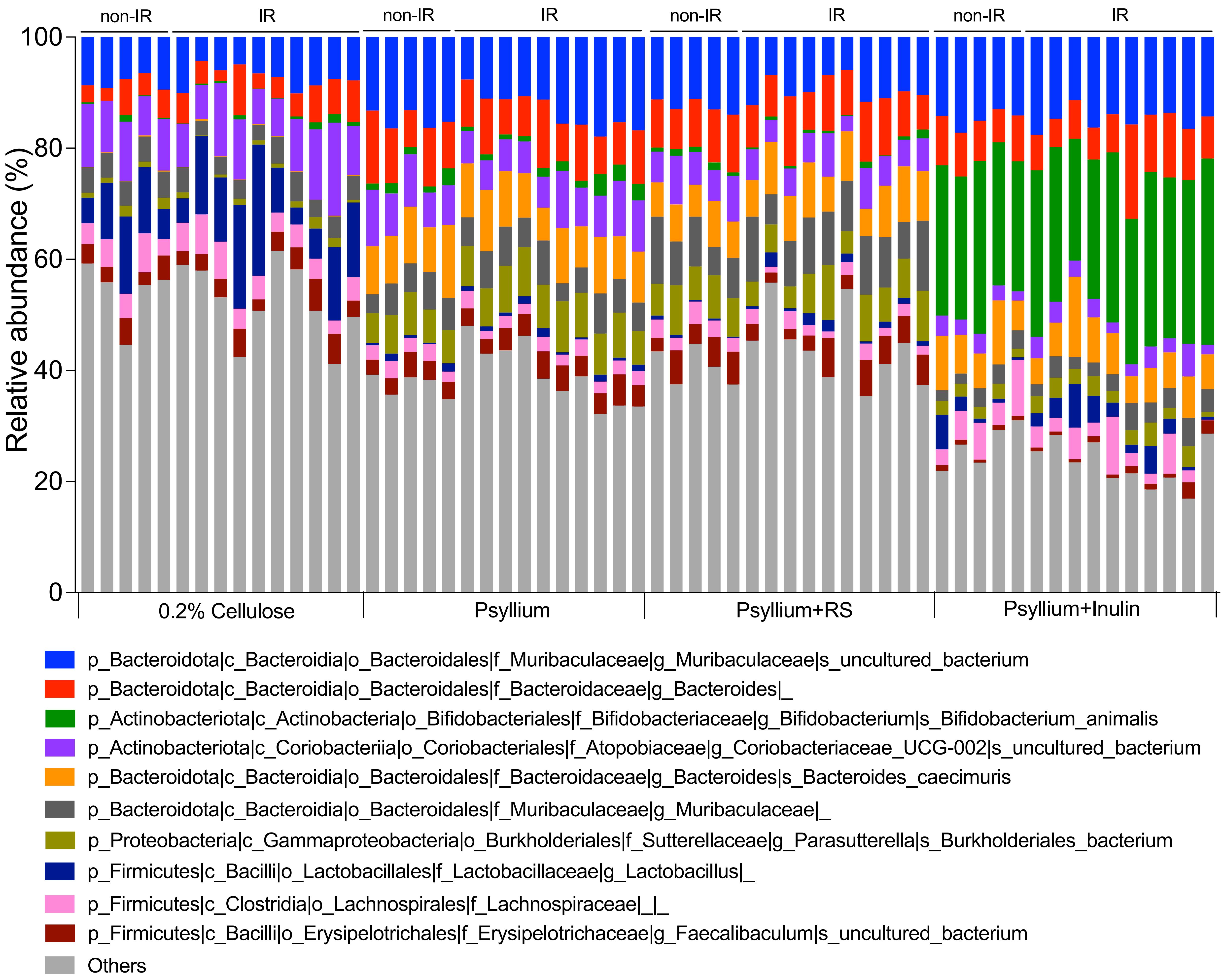

Supplementary Fig. 8 Phylogenetic composition of faecal microbiota when tumours reached 700 mm³. The sample sizes were n=5 in non-IR cohort and n=10 in IR cohort of each dietary group.

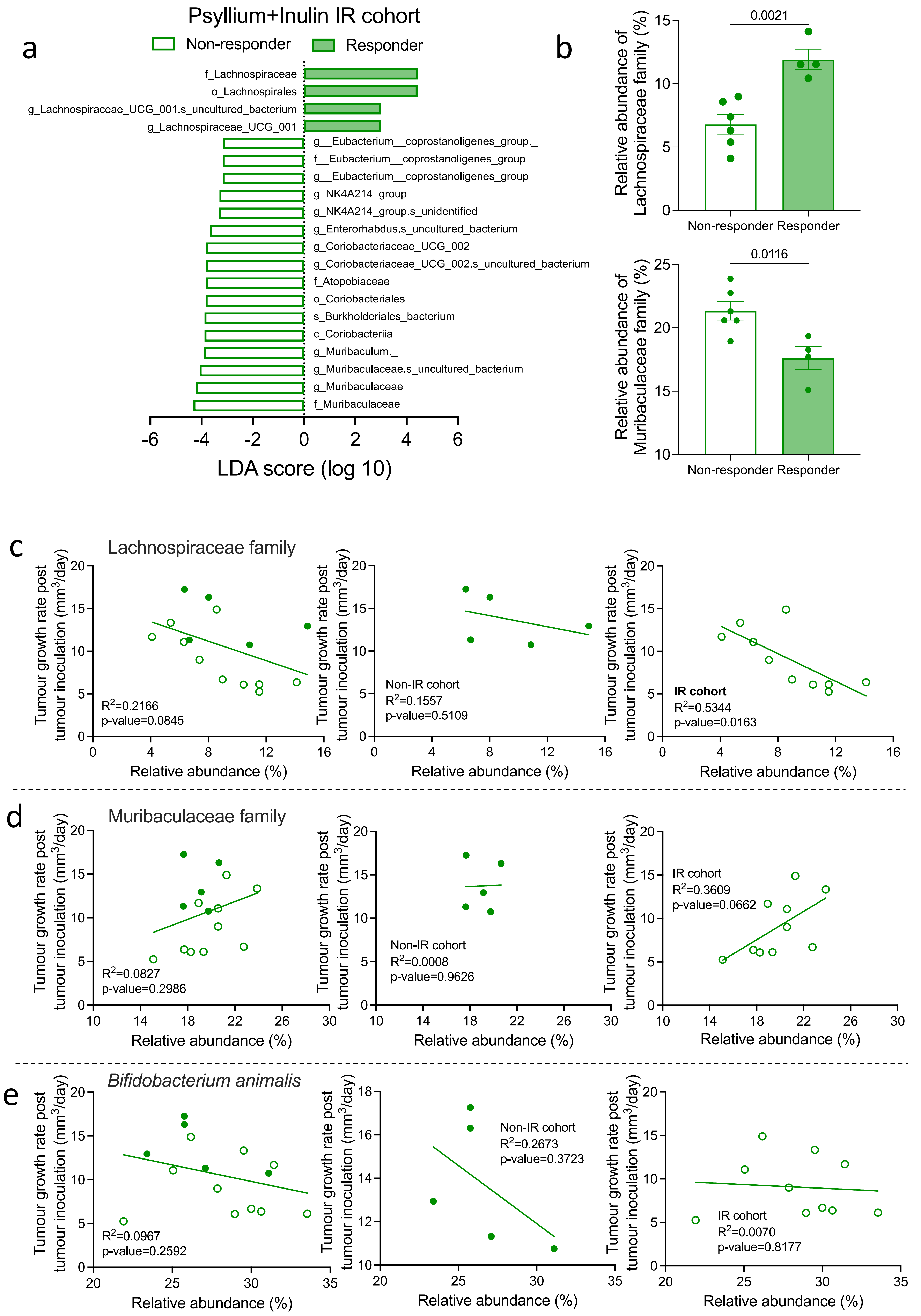
a
Psyllium+Inulin IR cohort
Non-responder
Responder
f_Lachnospiraceae
o_Lachnospirales
g_Lachnospiraceae_UCG_001.s_uncultured_bacterium
g_Lachnospiraceae_UCG_001
g__Eubacterium__coprostanoligenes_group._
f__Eubacterium__coprostanoligenes_group
g__Eubacterium__coprostanoligenes_group
g_NK4A214_group
g_NK4A214_group.s_unidentified
g_Enterorhabdus.s_uncultured_bacterium
g_Coriobacteriaceae_UCG_002
g_Coriobacteriaceae_UCG_002.s_uncultured_bacterium
f_Atopobiaceae
o_Coriobacteriales
s_Burkholderiales_bacterium
c_Coriobacteriia
g_Muribaculum._
g_Muribaculaceae.s_uncultured_bacterium
g_Muribaculaceae
f_Muribaculaceae
LDA score (log 10)
b
Relative abundance of Lachnospiraceae family (%)
0.0021
Non-responder Responder
Relative abundance of Muribaculaceae family (%)
0.0116
Non-responder Responder
c
Lachnospiraceae family
Tumour growth rate post tumour inoculation (mm³/day)
Relative abundance (%)
R²=0.2166
p-value=0.0845
Non-IR cohort
R²=0.1557
p-value=0.5109
IR cohort
R²=0.5344
p-value=0.0163
d
Muribaculaceae family
R²=0.0827
p-value=0.2986
Non-IR cohort
R²=0.0008
p-value=0.9626
IR cohort
R²=0.3609
p-value=0.0662
e
Bifidobacterium animalis
R²=0.0967
p-value=0.2592
Non-IR cohort
R²=0.2673
p-value=0.3723
IR cohort
R²=0.0070
p-value=0.8177

Supplementary Fig. 9 Differences in bacterial components in responders and non-responders in the psyllium plus inulin group. (a) Linear discriminant analysis (LDA) scores computed for differentially abundant taxa in the microbiomes of responders and non-responders. The alpha value was 0.05 for Kruskal-Wallis test and length of bar indicates the effect size associated with a taxon. (b) The relative abundances of Lachnospiraceae family and Muribaculaceae family between responders and non-responders were compared using two-tailed t-test. (c, d and e) Correlation between the Lachnospiraceae family, Muribaculaceae family and *Bifidobacterium animalis* versus the tumour growth in non-IR and IR cohorts of psyllium plus inulin. Tumour curve slopes were calculated by linear regression to represent tumour growth rates. The associations were assessed using the Pearson's correlation method.

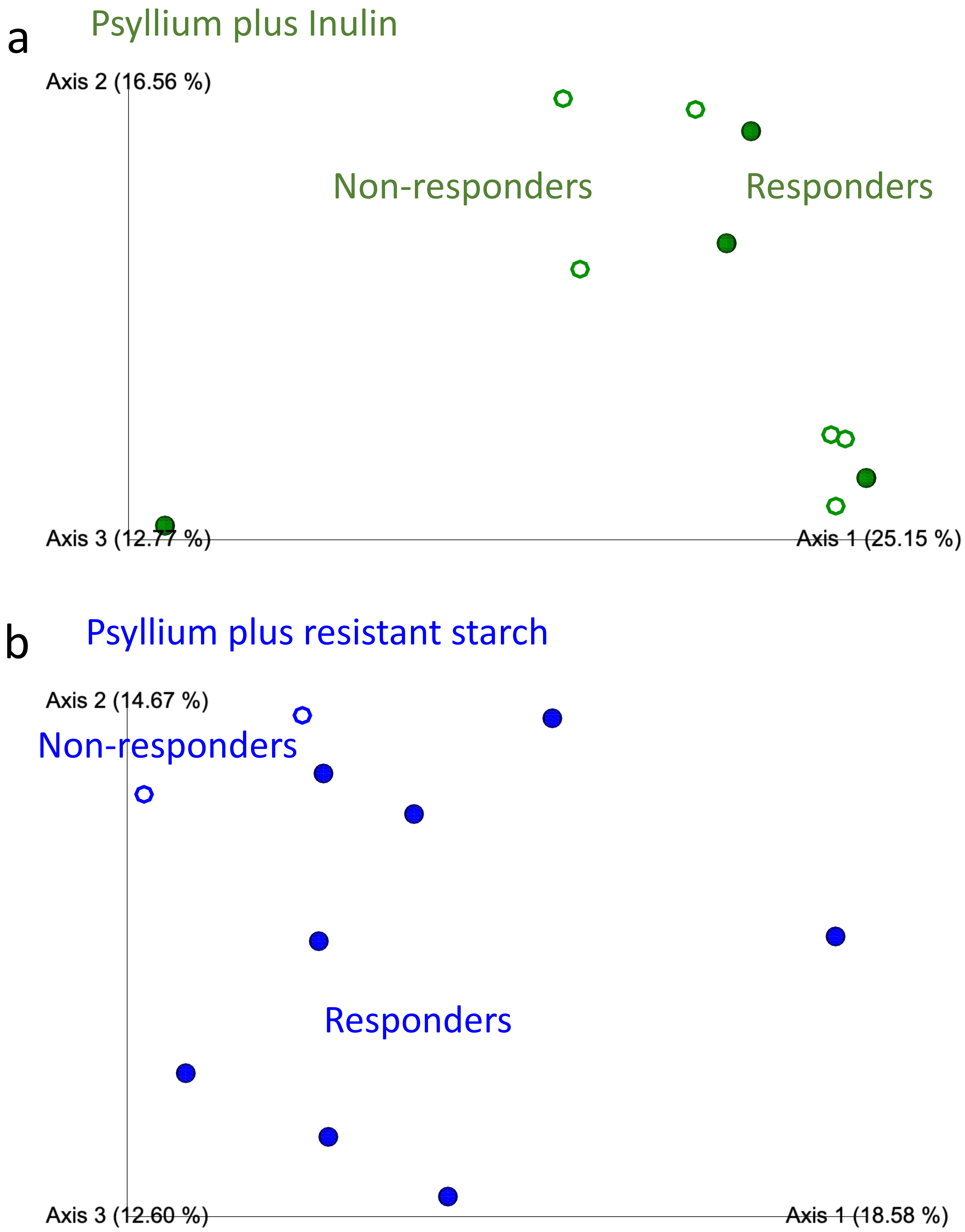

Supplementary Fig. 10 Principal coordinate analysis using Jaccard distance of faecal microbiota in the IR cohorts of (a) psyllium plus inulin or (b) psyllium plus RS. Faecal samples were collected when tumours reached 700 mm^3 (n=10/IR cohort in each dietary group). ADONIS test was used to assess the statistical significance of differences between the gut microbiota composition of responders and non-responders to irradiation within each dietary group.

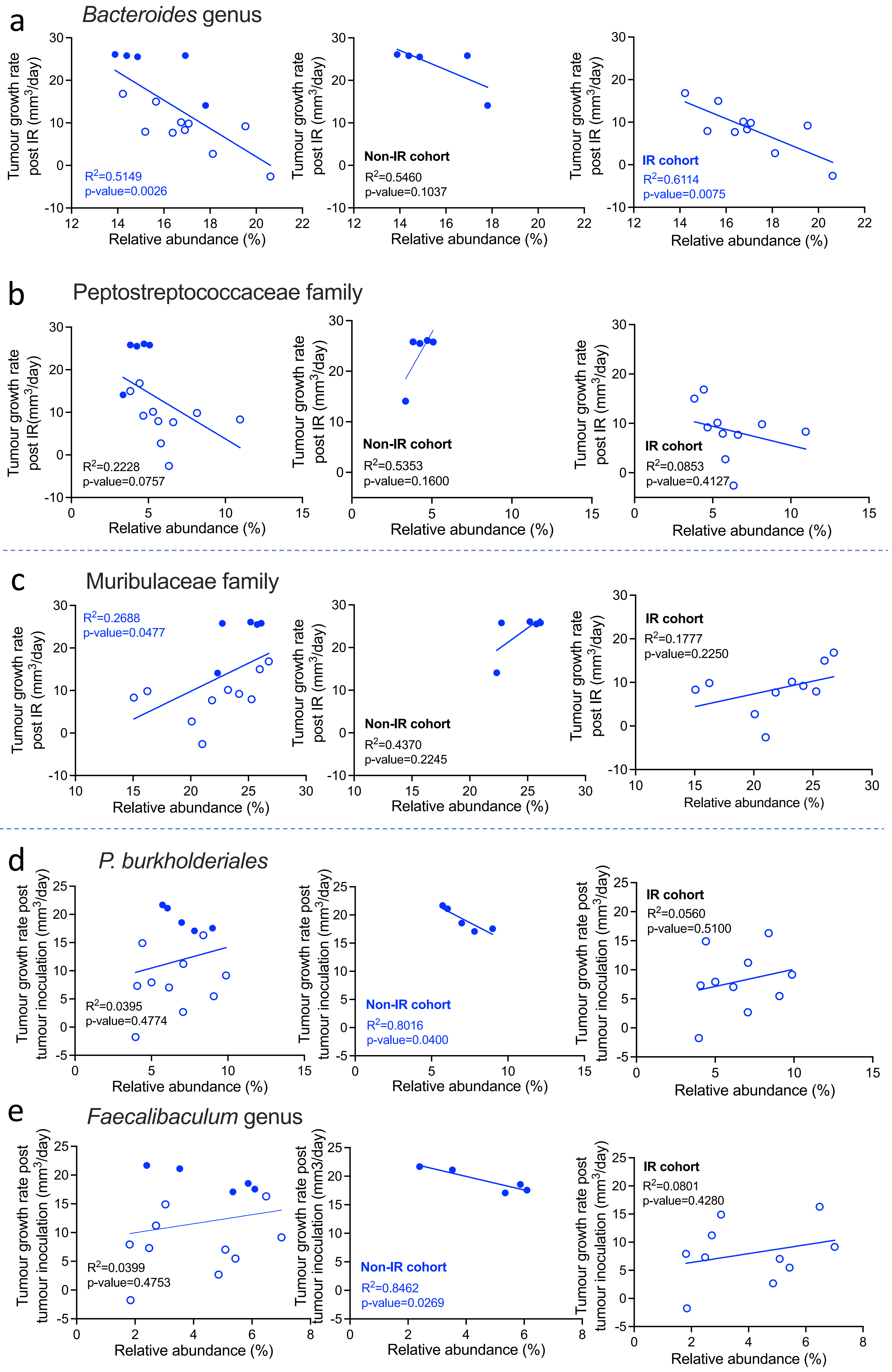

a
Bacteroides genus
Tumour growth rate post IR (mm3/day)
Relative abundance (%)
R2=0.5149
p-value=0.0026
Non-IR cohort
R2=0.5460
p-value=0.1037
IR cohort
R2=0.6114
p-value=0.0075
b
Peptostreptococcaceae family
R2=0.2228
p-value=0.0757
Non-IR cohort
R2=0.5353
p-value=0.1600
IR cohort
R2=0.0853
p-value=0.4127
c
Muribulaceae family
R2=0.2688
p-value=0.0477
Non-IR cohort
R2=0.4370
p-value=0.2245
IR cohort
R2=0.1777
p-value=0.2250
d
P. burkholderiales
Tumour growth rate post tumour inoculation (mm3/day)
R2=0.0395
p-value=0.4774
Non-IR cohort
R2=0.8016
p-value=0.0400
IR cohort
R2=0.0560
p-value=0.5100
e
Faecalibaculum genus
R2=0.0399
p-value=0.4753
Non-IR cohort
R2=0.8462
p-value=0.0269
IR cohort
R2=0.0801
p-value=0.4280

Supplementary Fig. 11 Correlation between the gut microbiota versus the tumour growth in non-IR and IR cohorts of psyllium plus RS. Tumour curve slopes were calculated by linear regression to represent tumour growth rates. Associations between the tumour growth rates and the following variables: (a) *Bacteroides* genus, (B) Peptostreptococcaceae family, (c) Muribulaceae family, (d) *P. burkholderiales* or (e) *Faecalibaculum* genus were determined using the Pearson's correlation method.

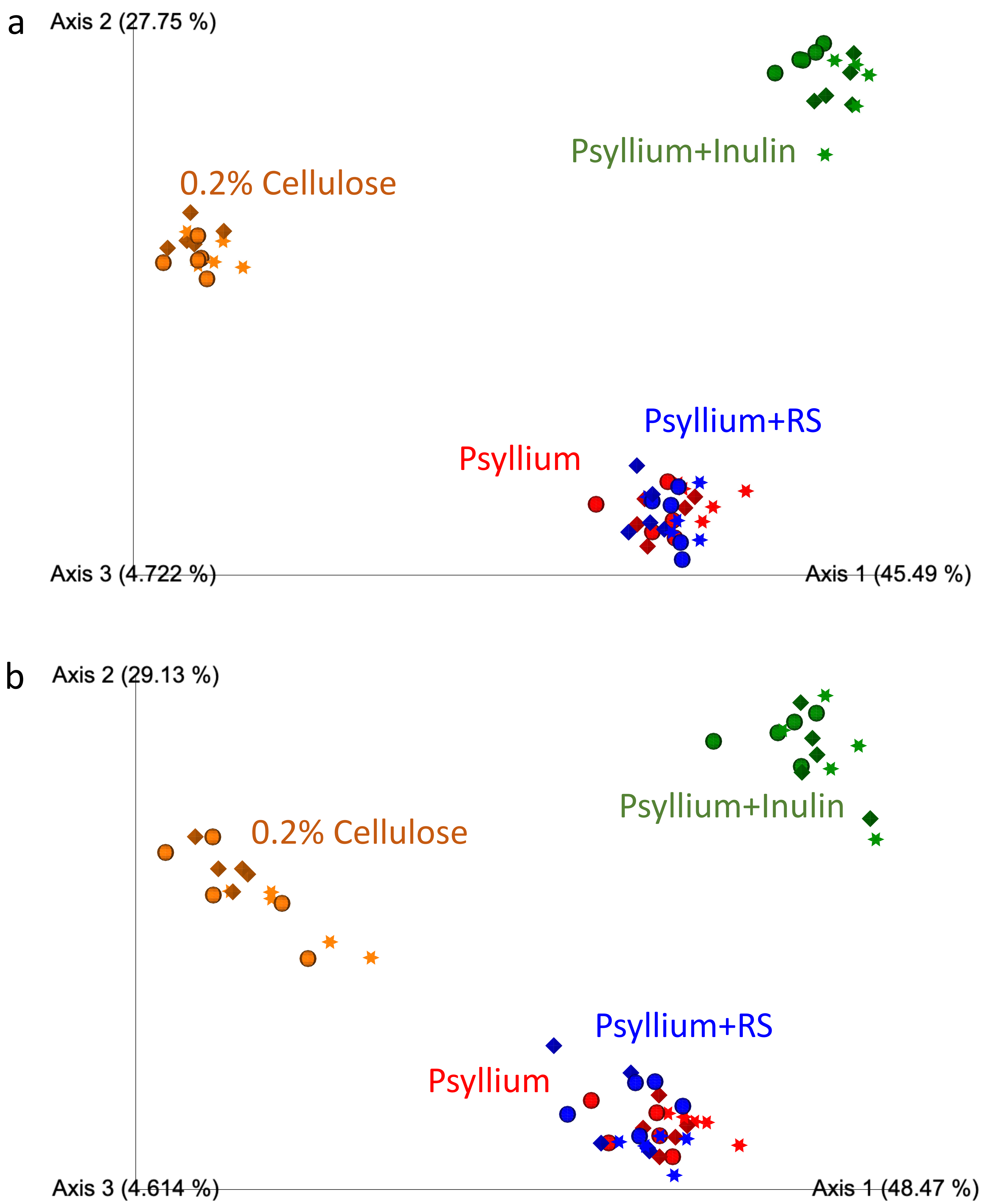

Supplementary Fig. 12 Beta diversity of faecal microbiota in the radiosensitisation experiment. Principal coordinate analysis using (a) unweighted (R^2=0.5498, Pr(>F)=0.11) and (b) weighted UniFrac (R^2=0.7975, Pr(>F)=0.11) of faecal microbiotas (n=5/non-IR cohort, n=5/IR cohort in each dietary group) that were collected when tumours reached 700 mm^3 based on cages (sphere, diamond and star denote three different cages) and dietary groups (orange-0.2% cellulose, red-psyllium, blue-psyllium+RS and green-psyllium+Inulin). ADONIS test was used to confirm the existence of significant differences among different dietary groups in terms of gut microbiota composition.

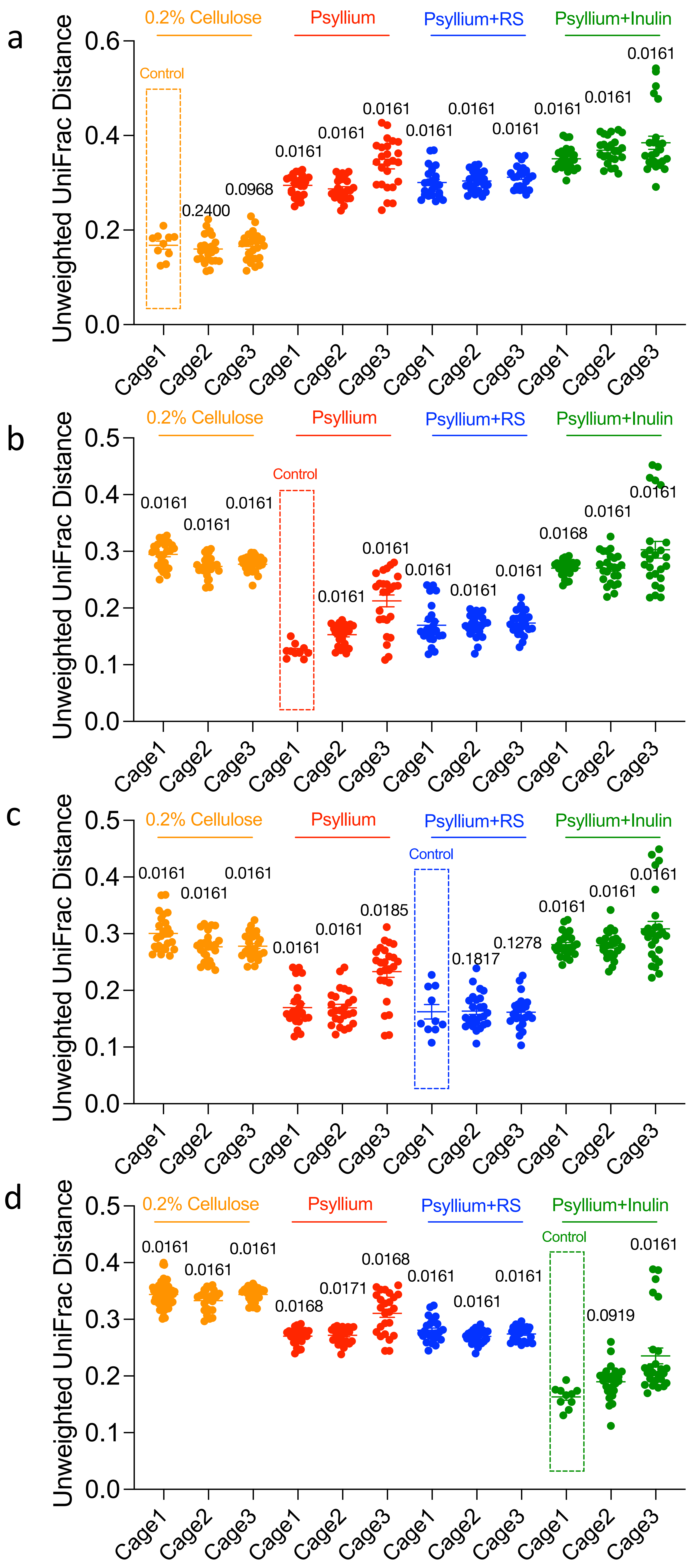
a
b
c
d
Unweighted UniFrac Distance
0.2% Cellulose
Psyllium
Psyllium+RS
Psyllium+Inulin
Control
Cage1
Cage2
Cage3
0.2400
0.0968
0.0161
0.0168
0.0185
0.1817
0.1278
0.0171
0.0919

Supplementary Fig. 13 Unweighted UniFrac distance of faecal microbiota between cages in the radiosensitisation experiment. PERMANOVA test using the pseudo-F method was used to test for statistically significant inter-cage differences of the unweighted UniFrac distances of faecal microbiotas (n=5/cage) that were collected when tumours reached 700 mm^3 based on cages. For each comparisons, the first cage (dashed box) of each dietary groups, including (a) 0.2% cellulose, (b) psyllium, (c) psyllium plus resistant starch, or (d) psyllium plus inulin, was used as the control to measure its distance to the gut microbiota of the other eleven cages. For the control, only ten values were generated from the comparison of five mice with each other within the same cage. Twenty-five values were generated from the comparison of five mice from any two cages. Data are presented as mean±SEM.

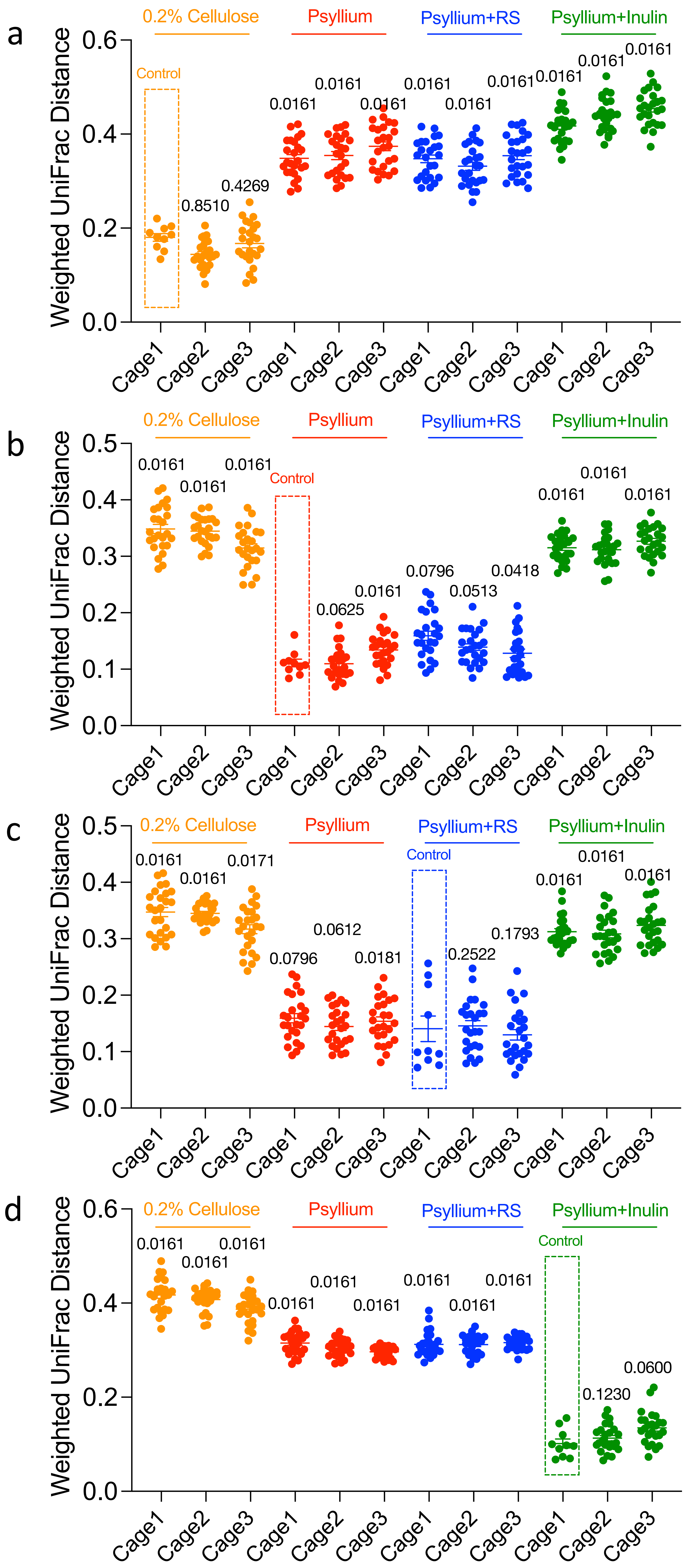

a
b
c
d
Weighted UniFrac Distance
0.2% Cellulose
Psyllium
Psyllium+RS
Psyllium+Inulin
Control
Cage1
Cage2
Cage3
0.8510
0.4269
0.0161
0.0625
0.0796
0.0513
0.0418
0.0171
0.0612
0.0181
0.2522
0.1793
0.1230
0.0600

Supplementary Fig. 14 Weighted UniFrac distance of faecal microbiota between cages in the radiosensitisation experiment. PERMANOVA test using the pseudo-F method was used to test for statistically significant inter-cage differences of the weighted UniFrac distances of faecal microbiotas (n=5/cage) that were collected when tumours reached 700 mm^3 based on cages. For each comparisons, the first cage (dashed box) of each dietary groups, including (a) 0.2% cellulose, (b) psyllium, (c) psyllium plus resistant starch, or (d) psyllium plus inulin, was used as the control to measure its distance to the gut microbiota of the other eleven cages. For the control, only ten values were generated from the comparison of five mice with each other within the same cage. Twenty-five values were generated from the comparison of five mice from any two cages. Data are presented as mean±SEM.

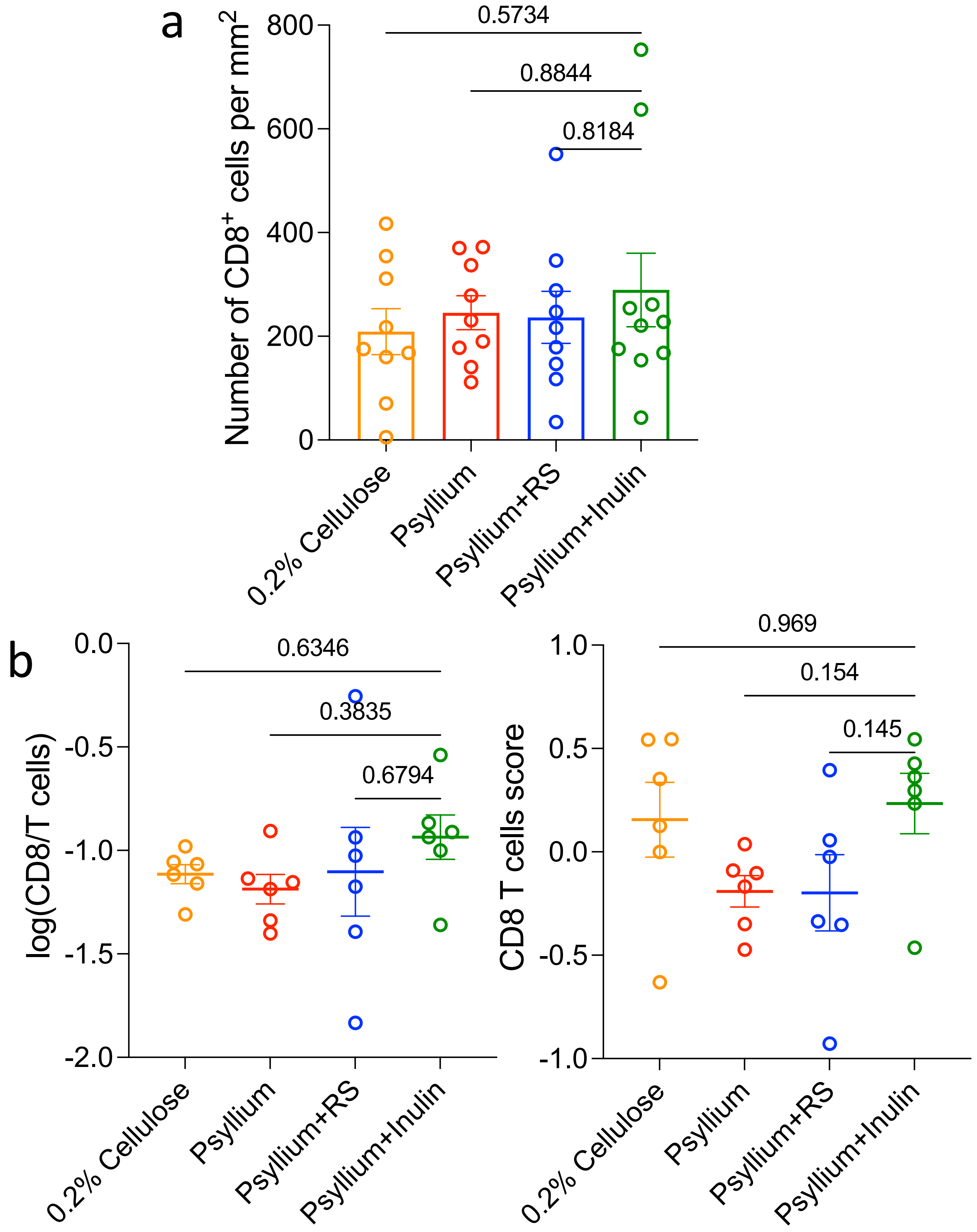

Supplementary Fig. 15 Local tumour cytotoxic T cells in the IR cohorts of all dietary groups. (a) IHC staining to assess the numbers of cytotoxic T cells in the irradiated tumours (n=10/group). (b) NanoString analysis of $CD8^+$ cells and their ratio over T cells to assess the populations of cytotoxic T cells in the irradiated tumours (n=6/group). The expression of each immune cell's marker genes was normalised by a reference gene set and cell scores were calculated as the log2(average of normalised gene expression). One-way ANOVA with Bonferroni's multiple comparison test was used to compare the means among different dietary groups. Data are presented as mean ± SEM.

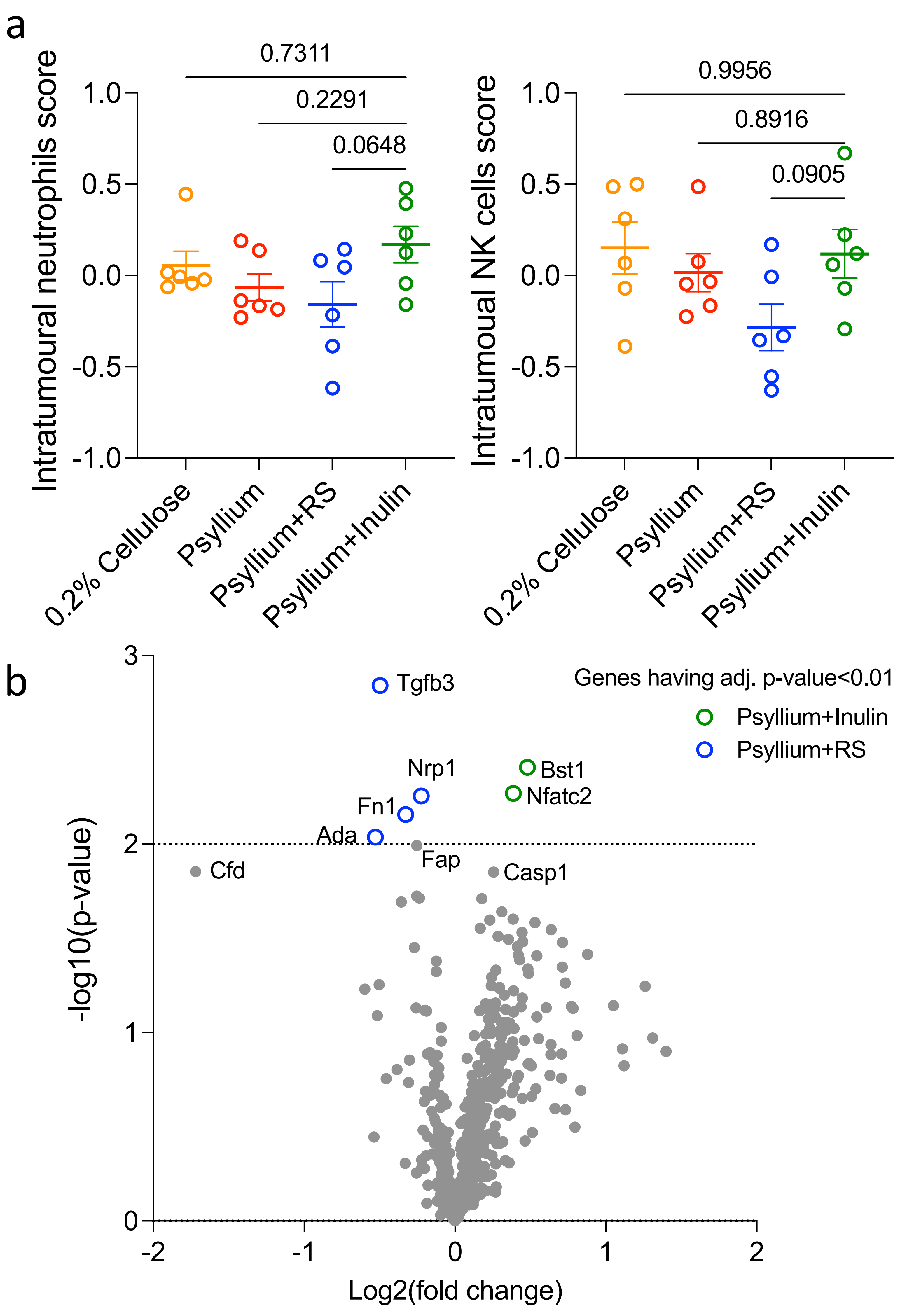

Supplementary Fig. 16 Local tumour immune responses in the IR cohorts of all dietary groups and. (a) NanoString analysis of neutrophils and NK cells in the irradiated tumours (n=10/group). One-way ANOVA with Bonferroni's multiple comparison test was used to compare the means among different dietary groups. (b) Differential immune-related gene expression between psyllium plus RS and psyllium plus inulin groups (n=6/group). The result was partitioned by psyllium plus RS. The Benjamin-Hochberg method to correct p-values for controlling the false discovery rate (FDR). A negative log2(fold change) indicates the gene overexpressed in psyllium plus RS group and a positive value indicates the gene overexpressed in psyllium plus inulin group.

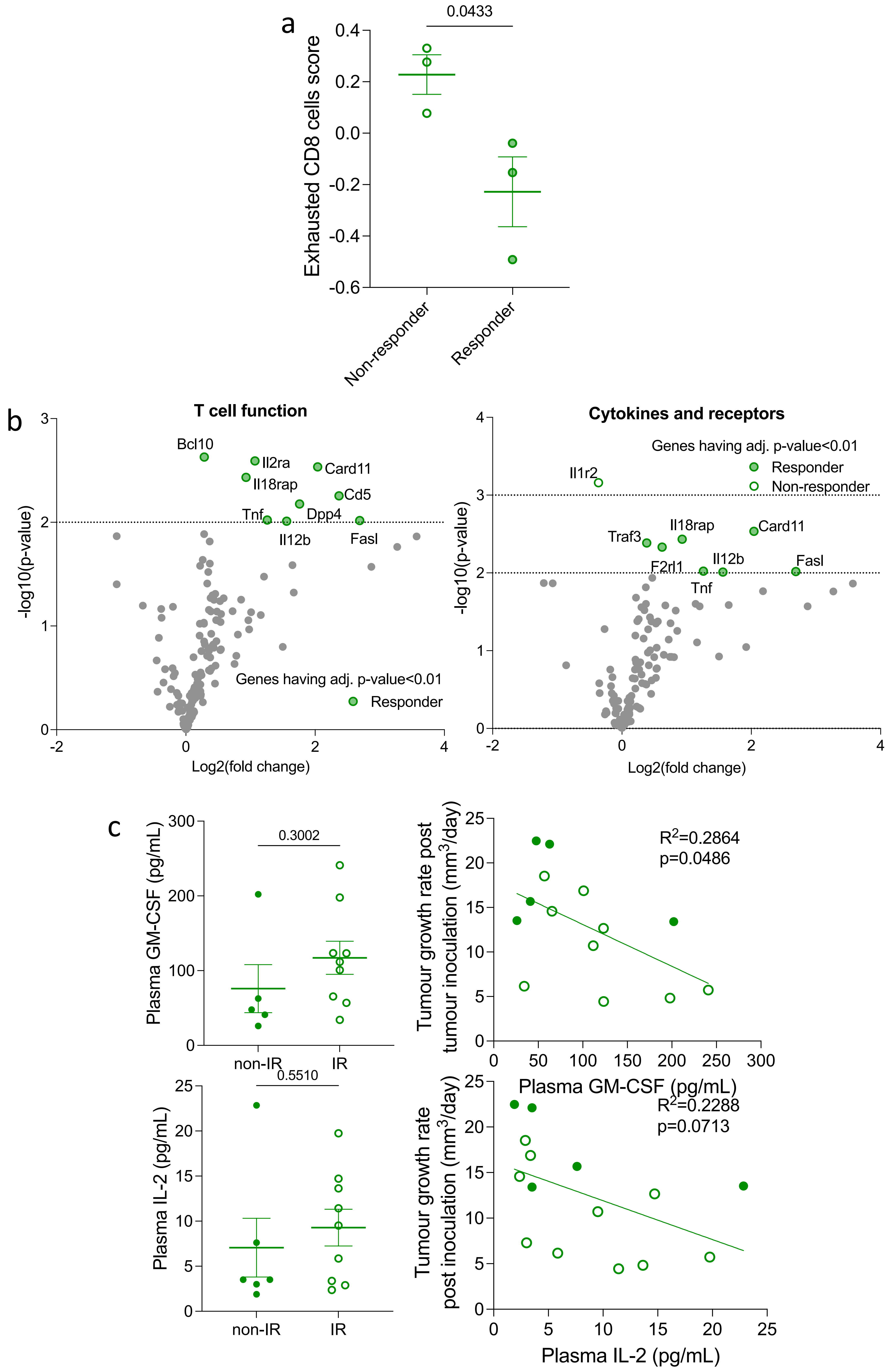
a
0.0433
Exhausted CD8 cells score
0.4
0.2
0.0
-0.2
-0.4
-0.6
Non-responder
Responder
b
T cell function
Bcl10
Il2ra
Il18rap
Card11
Cd5
Tnf
Dpp4
Il12b
Fasl
Genes having adj. p-value<0.01
Responder
-log10(p-value)
Log2(fold change)
Cytokines and receptors
Genes having adj. p-value<0.01
Responder
Non-responder
Il1r2
Traf3
Il18rap
Card11
F2rl1
Il12b
Fasl
Tnf
c
Plasma GM-CSF (pg/mL)
0.3002
non-IR
IR
Tumour growth rate post tumour inoculation (mm³/day)
R²=0.2864
p=0.0486
Plasma GM-CSF (pg/mL)
Plasma IL-2 (pg/mL)
0.5510
non-IR
IR
Tumour growth rate post tumour inoculation (mm³/day)
R²=0.2288
p=0.0713
Plasma IL-2 (pg/mL)

Supplementary Fig. 17 Local tumour and systemic immunity in psyllium plus inulin stratified by tumour response and IR. (a) NanoString analysis of exhausted CD8 cells and (b) gene sets of pathways of cytokines and receptors and T cell function on tumours in responders and non-responders in psyllium plus inulin groups (n=3/group). The cell scores between responders and non-responders were compared by two-tailed t-test. The differential immune-related gene expression analysis was followed the Benjamin-Hochberg method to correct p-values for controlling the false discovery rate (FDR). (c) Levels of plasma Th1 cytokines, GM-CSF and IL-2, stratified by IR. Correlations of these cytokines versus tumour growth rates in psyllium plus inulin group were assessed using the Pearson's correlation method. Data are presented as mean ± SEM.

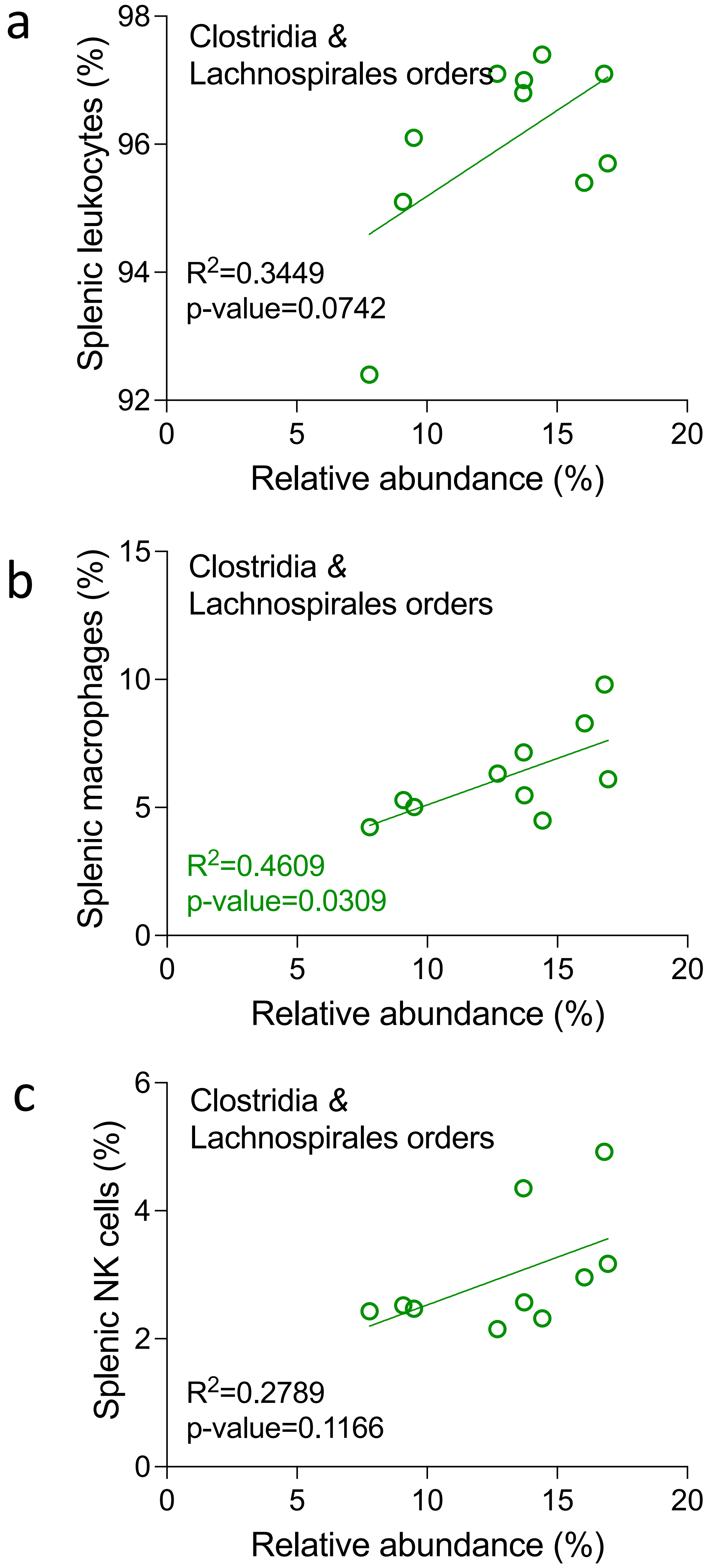

Supplementary Fig. 18 Correlations between the Clostridia and Lachnospirales orders and the populations of splenic (a) leukocytes, (b) macrophages, and (c) natural killer cells in the IR cohorts of psyllium plus inulin group. The associations were assessed using the Pearson's correlation method.

a

	Slope	R^2	p-value
Cysteine	0.1000	0.7186	0.0020
Deoxyribose	0.0575	0.656	0.004
Malic acid	0.1056	0.6124	0.007
Caffeic acid	0.0871	0.5448	0.0148
Isoferulic acid	-0.0618	0.5408	0.0154
Pyruvic acid	0.0534	0.4350	0.0380
2-Ketobutyric acid	0.0773	0.3994	0.0500

b

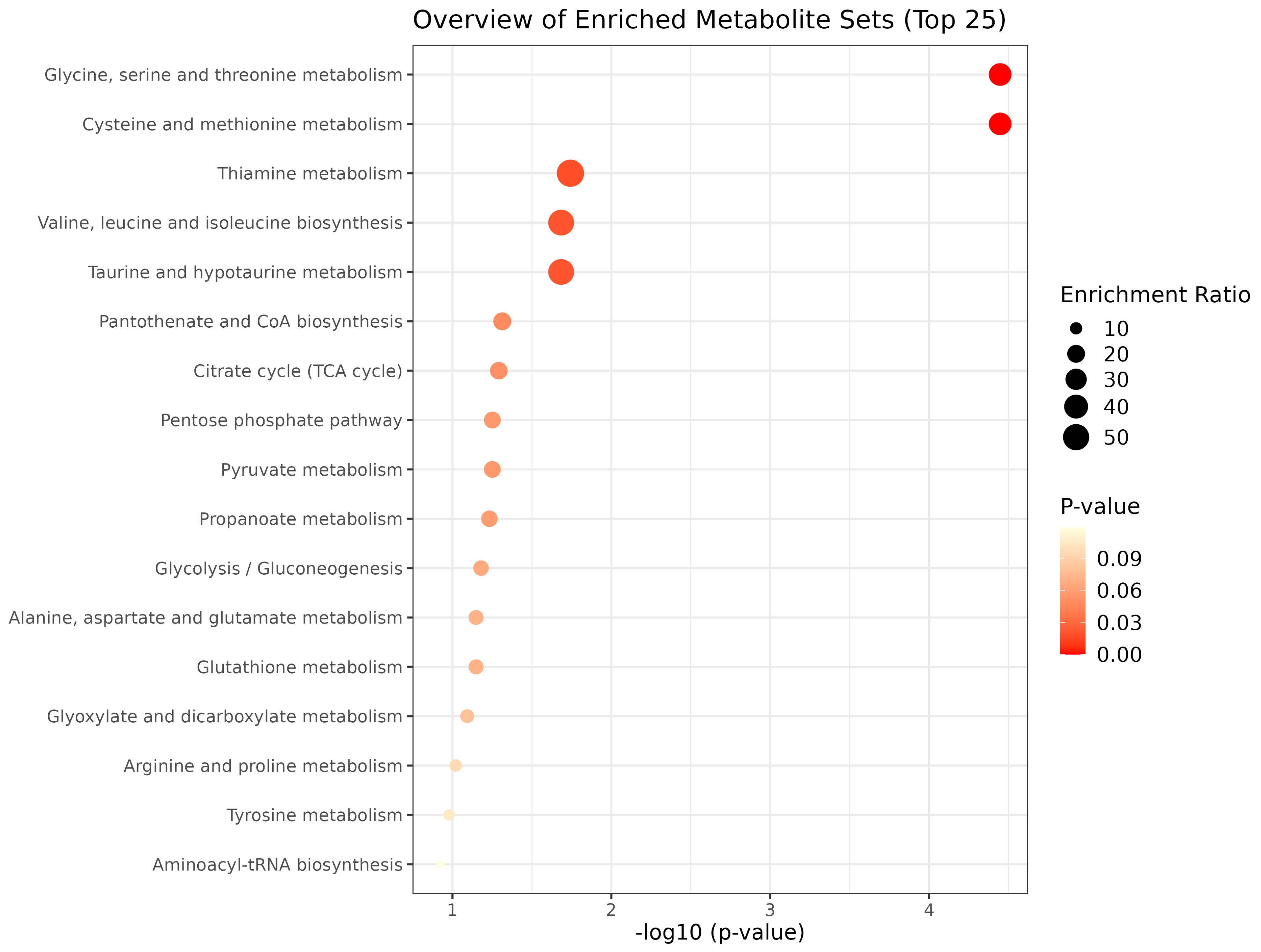

Supplementary Fig. 19 Metabolites and KEGG pathway that were associated with tumour growth in mice fed with psyllium plus RS. (a) The top table listed the metabolites that positively (no colour) or negatively (blue) correlated to tumour growth in psyllium plus RS group. The associations were assessed using the Pearson's correlation method. (b) The figure below showed KEGG pathways enrichment analysis of metabolites that positively correlated to the tumour growth in psyllium plus RS. The intensity of colour denotes the p-value and the dot size denotes the enrichment ratio.

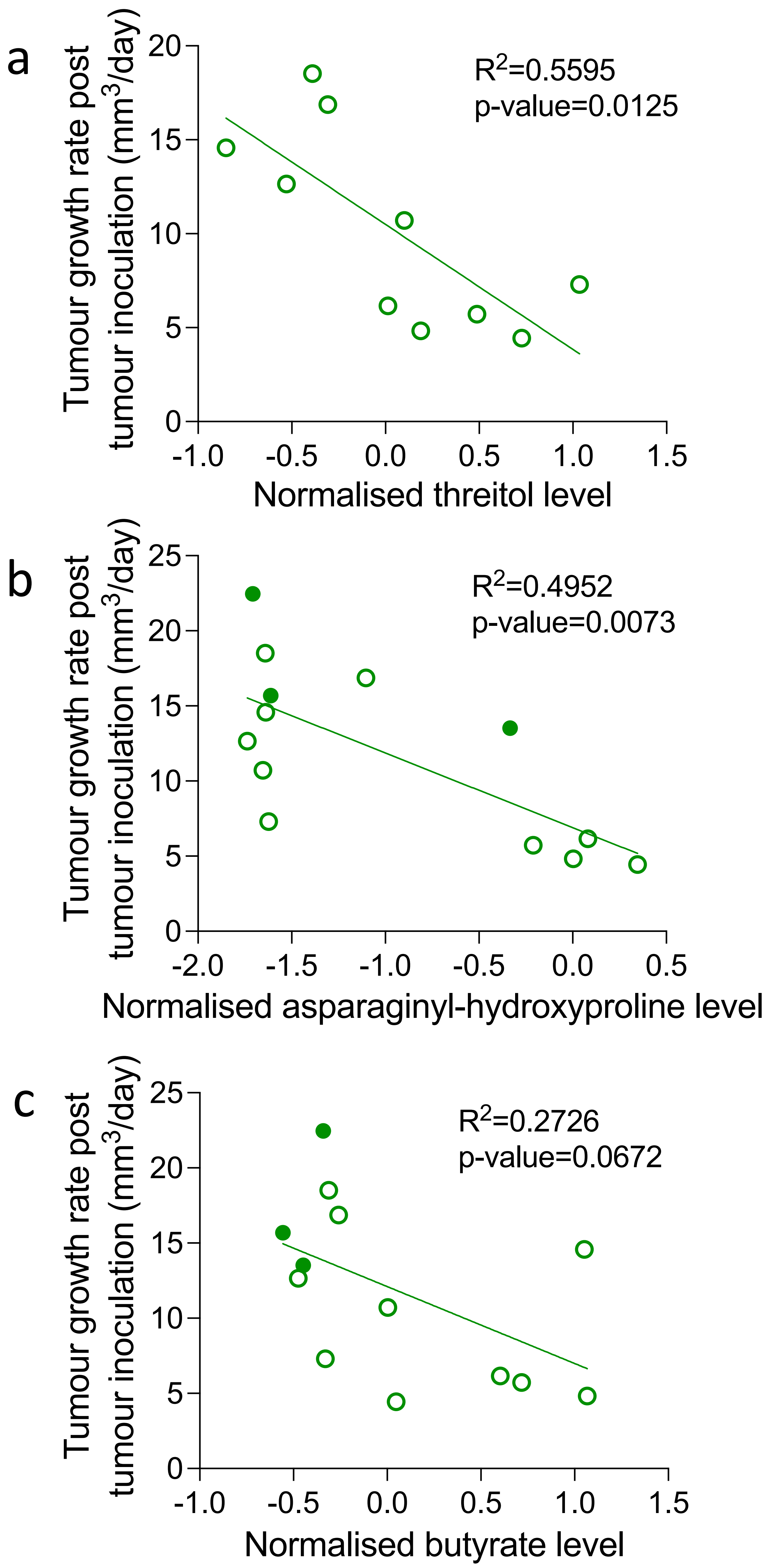

Supplementary Fig. 20 Correlations between the caecal (a) threitol, (b) asparaginyl-hydroxyproline and (c) butyrate levels versus the tumour growth rate in IR cohort with or without non-IR cohort in the psyllium plus inulin group. The associations were assessed using the Pearson's correlation method.

a

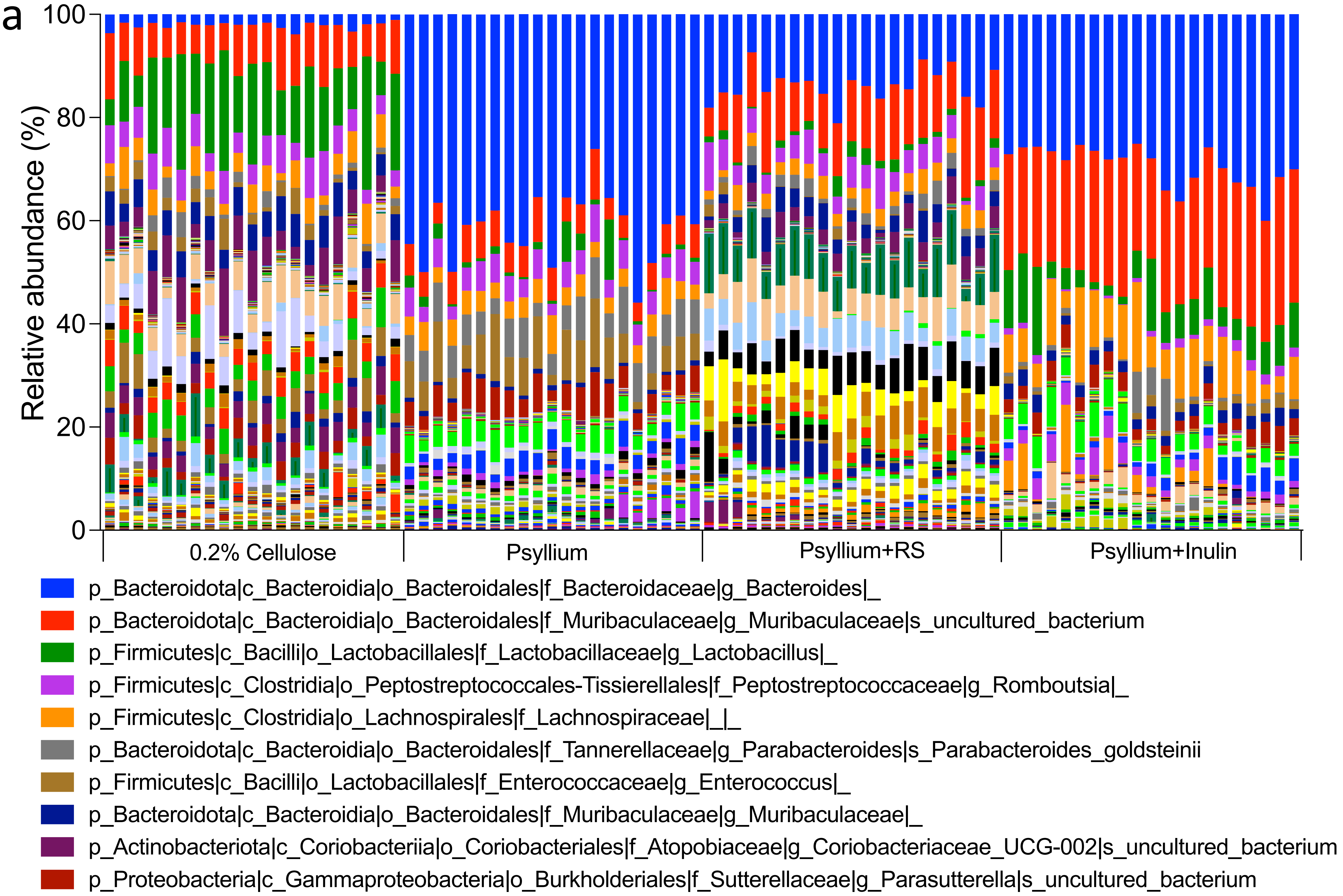
Relative abundance (%)
100
80
60
40
20
0
0.2% Cellulose
Psyllium
Psyllium+RS
Psyllium+Inulin
p_Bacteroidota|c_Bacteroidia|o_Bacteroidales|f_Bacteroidaceae|g_Bacteroides|_
p_Bacteroidota|c_Bacteroidia|o_Bacteroidales|f_Muribaculaceae|g_Muribaculaceae|s_uncultured_bacterium
p_Firmicutes|c_Bacilli|o_Lactobacillales|f_Lactobacillaceae|g_Lactobacillus|_
p_Firmicutes|c_Clostridia|o_Peptostreptococcales-Tissierellales|f_Peptostreptococcaceae|g_Romboutsia|_
p_Firmicutes|c_Clostridia|o_Lachnospirales|f_Lachnospiraceae|_|_
p_Bacteroidota|c_Bacteroidia|o_Bacteroidales|f_Tannerellaceae|g_Parabacteroides|s_Parabacteroides_goldsteinii
p_Firmicutes|c_Bacilli|o_Lactobacillales|f_Enterococcaceae|g_Enterococcus|_
p_Bacteroidota|c_Bacteroidia|o_Bacteroidales|f_Muribaculaceae|g_Muribaculaceae|_
p_Actinobacteriota|c_Coriobacteriia|o_Coriobacteriales|f_Atopobiaceae|g_Coriobacteriaceae_UCG-002|s_uncultured_bacterium
p_Proteobacteria|c_Gammaproteobacteria|o_Burkholderiales|f_Sutterellaceae|g_Parasutterella|s_uncultured_bacterium

b

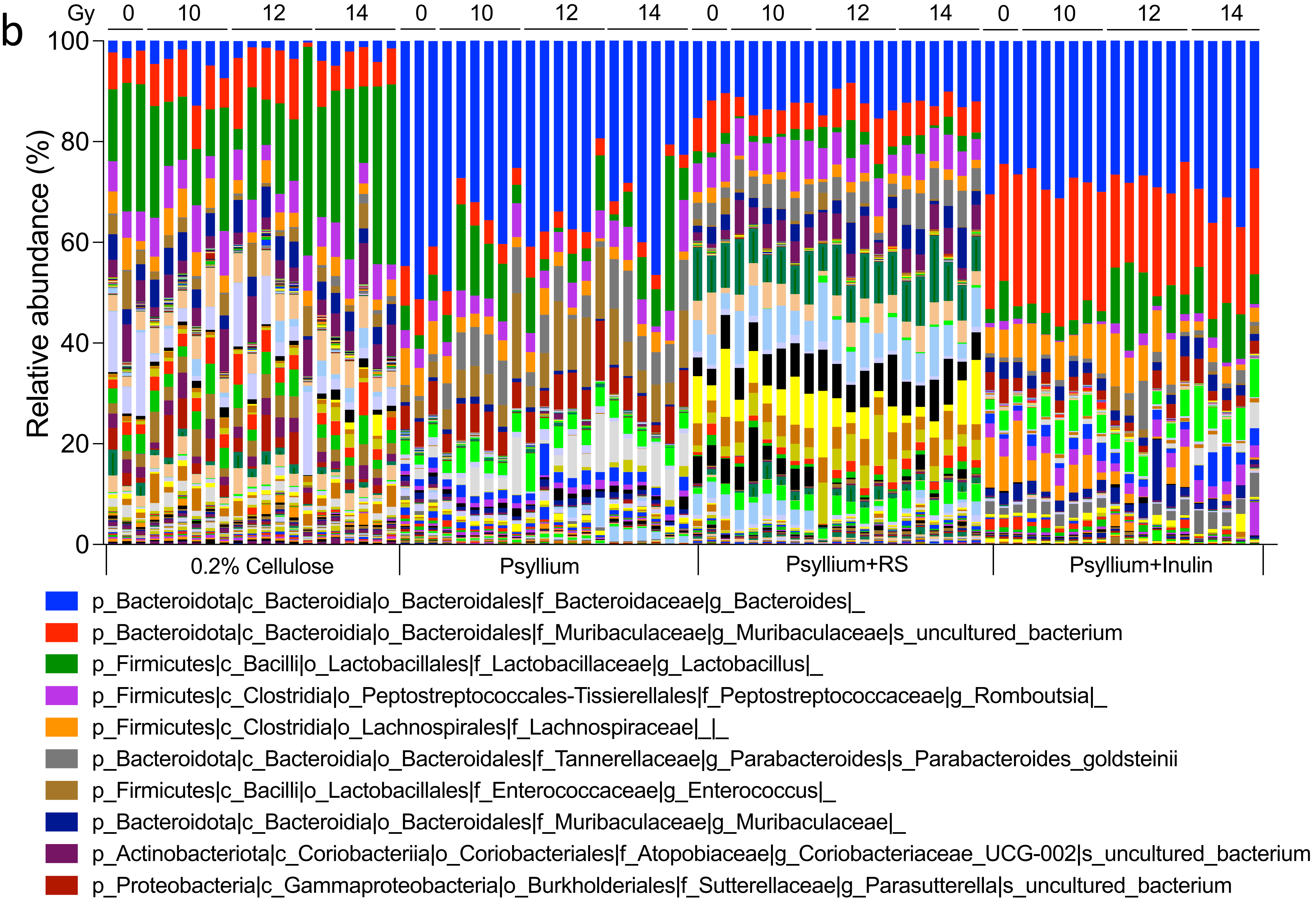
Gy
0
10
12
14
0
10
12
14
0
10
12
14
0
10
12
14
Relative abundance (%)
100
80
60
40
20
0
0.2% Cellulose
Psyllium
Psyllium+RS
Psyllium+Inulin
p_Bacteroidota|c_Bacteroidia|o_Bacteroidales|f_Bacteroidaceae|g_Bacteroides|_
p_Bacteroidota|c_Bacteroidia|o_Bacteroidales|f_Muribaculaceae|g_Muribaculaceae|s_uncultured_bacterium
p_Firmicutes|c_Bacilli|o_Lactobacillales|f_Lactobacillaceae|g_Lactobacillus|_
p_Firmicutes|c_Clostridia|o_Peptostreptococcales-Tissierellales|f_Peptostreptococcaceae|g_Romboutsia|_
p_Firmicutes|c_Clostridia|o_Lachnospirales|f_Lachnospiraceae|_|_
p_Bacteroidota|c_Bacteroidia|o_Bacteroidales|f_Tannerellaceae|g_Parabacteroides|s_Parabacteroides_goldsteinii
p_Firmicutes|c_Bacilli|o_Lactobacillales|f_Enterococcaceae|g_Enterococcus|_
p_Bacteroidota|c_Bacteroidia|o_Bacteroidales|f_Muribaculaceae|g_Muribaculaceae|_
p_Actinobacteriota|c_Coriobacteriia|o_Coriobacteriales|f_Atopobiaceae|g_Coriobacteriaceae_UCG-002|s_uncultured_bacterium
p_Proteobacteria|c_Gammaproteobacteria|o_Burkholderiales|f_Sutterellaceae|g_Parasutterella|s_uncultured_bacterium

Supplementary Fig. 21 Phylogenetic composition of faecal microbiota before and after irradiation in the acute toxicity experiment. Faecal samples were collected (a) pre-IR and (b) 3.75 days post-IR (n=3/non-IR cohort, n=6/IR cohort in each IR doses and dietary group). The samples are presented in the same order in the top and bottom panels.

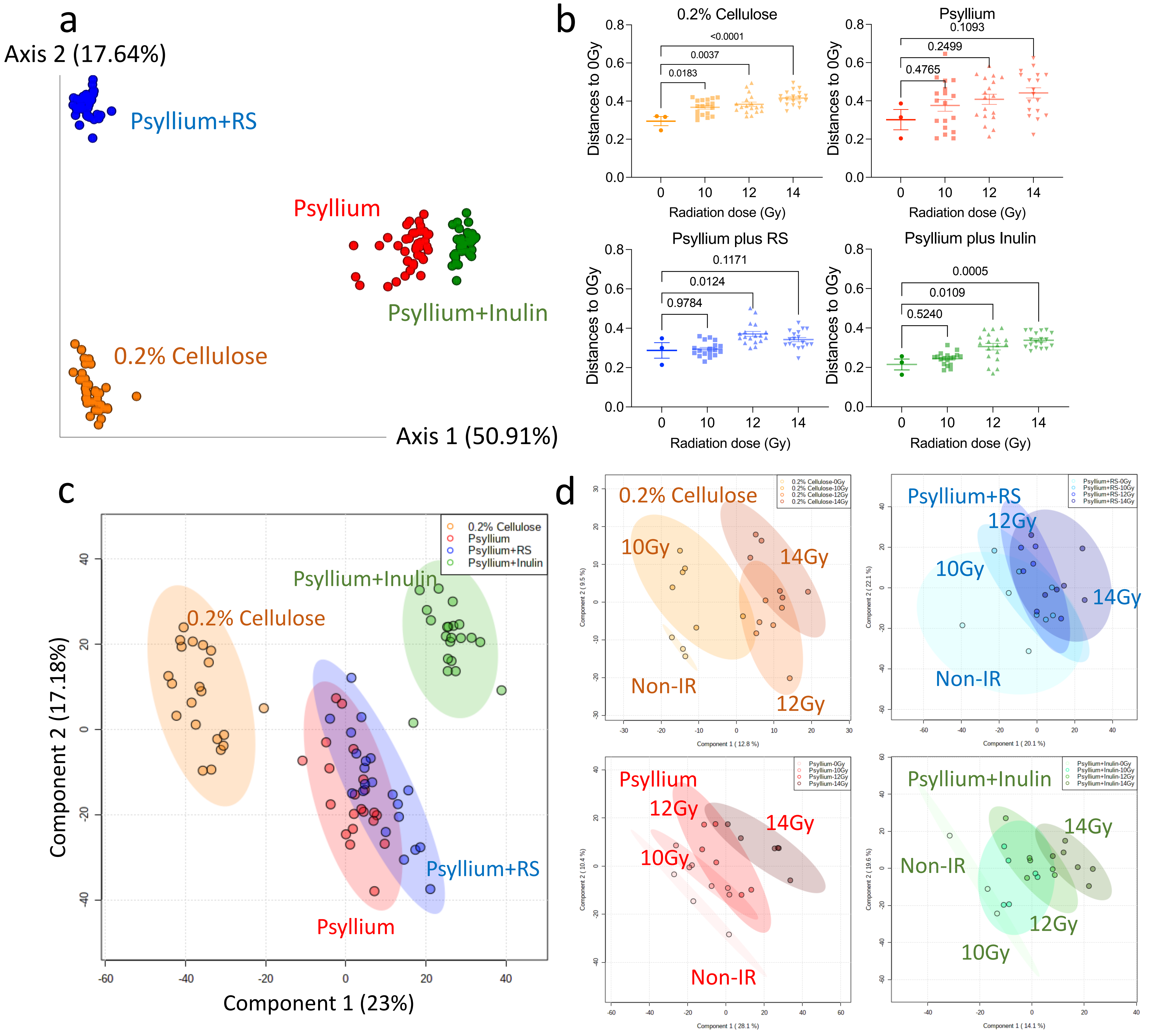

Supplementary Fig. 22 Beta diversity of the gut microbiota and the metabolites profile in non-tumour-bearing mice after 3-week modified diet and 3.75 days after SARRP IR. (a) Principal coordinate analysis of faecal microbiota using Bray-Curtis dissimilarity. ADONIS test was used to confirm the existence of significant group differences in terms of gut microbiota composition. (b) Distances of gut microbiota in irradiated mice compared to non-IR controls in each dietary groups. PERMANOVA with the pseudo-F statistic was used to evaluate the statistical significance of differences in Bray-Curtis dissimilarity of gut microbiota among different IR doses. (c) Principal component analysis of metabolites profile in all and each dietary groups. ADONIS test was used to confirm the existence of significant group differences in terms of metabolite profiles. (d) Distances of metabolites profile in irradiated mice compared to non-IR controls in each dietary groups. The intensity of colour reflects the IR dose. ADONIS test was used to assess the presence of significant group differences in the Euclidean distance matrices of metabolite profiles among various radiation (IR) doses.

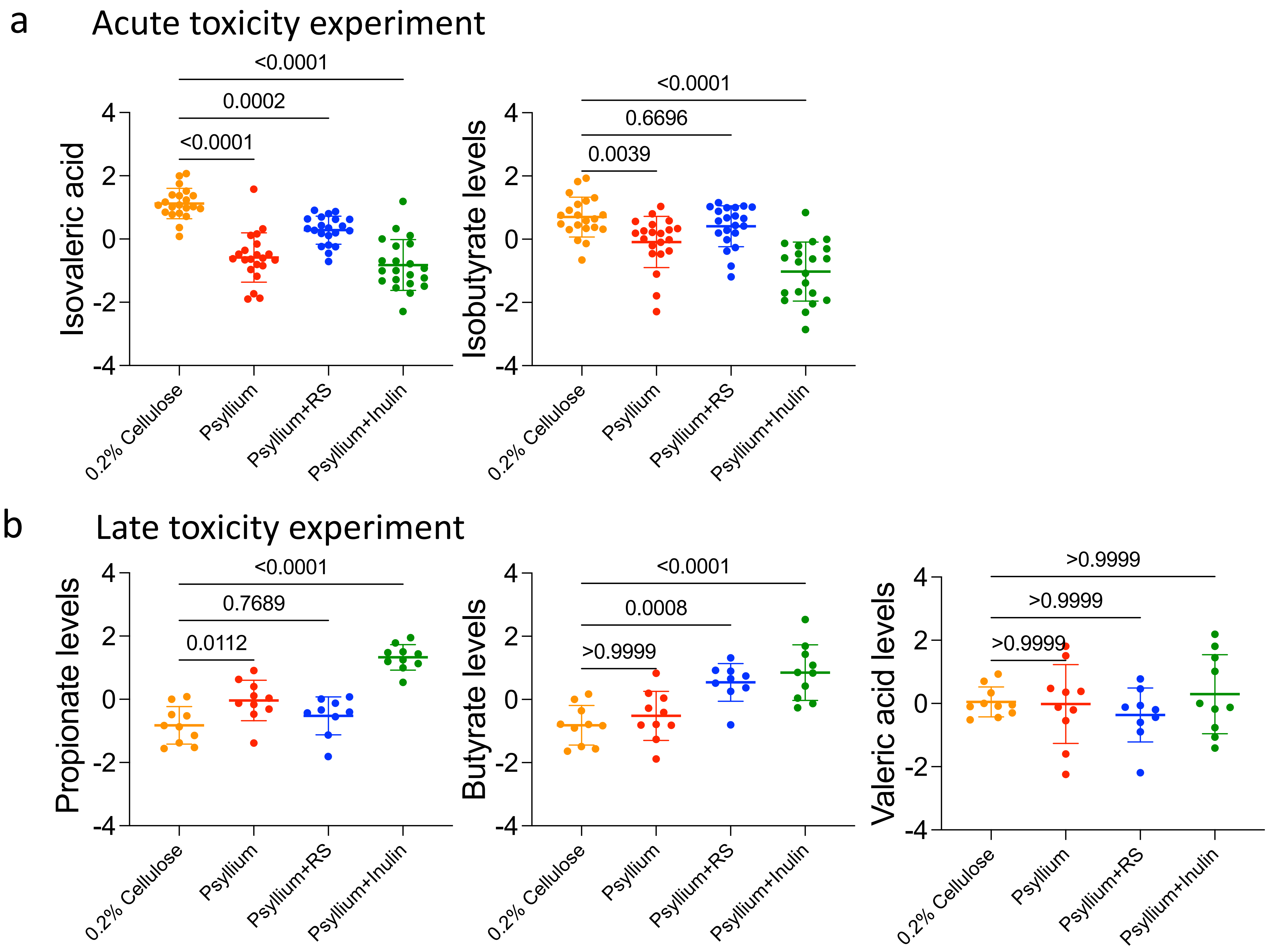

Supplementary Fig. 23 Caecal SCFAs in non-tumour-bearing mice after 3-week modified diet in acute toxicity and late toxicity experiments. Relative levels of (a) isovaleric acid and isobutyrate in the acute toxicity experiment, and (b) propionate, butyrate and valeric acid in the late toxicity experiment were compared among groups using one-way ANOVA with Bonferroni's multiple comparison test.

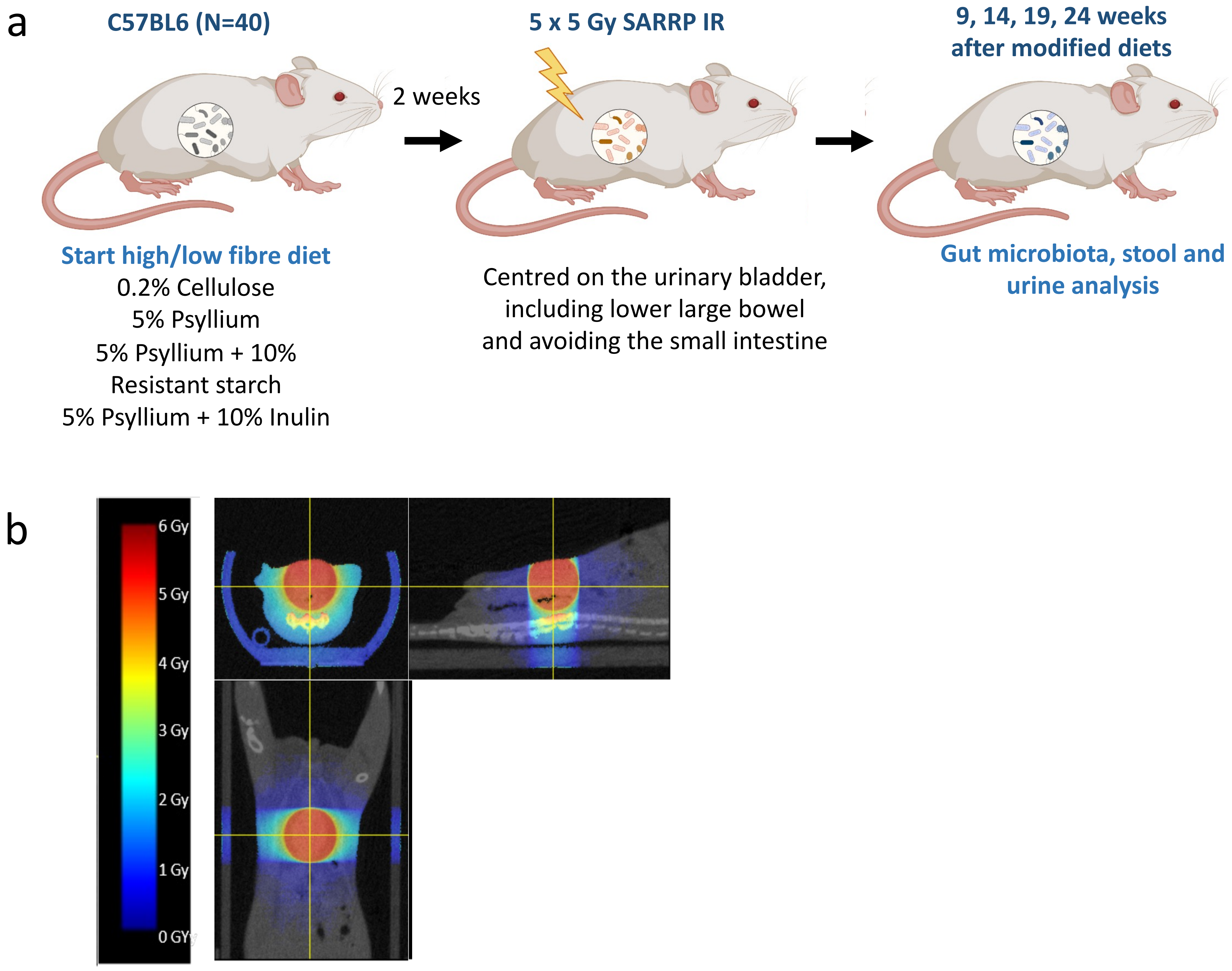

Supplementary Fig. 24 Overview of late normal tissue toxicity experiment. (a) C57BL/6 mice were treated supine with 5 Gy SARRP IR head down for 5 consecutive days to their lower abdomen after 2-weeks of modified diet. Retrieved from https://app.biorender.com/biorender-templates. (b) Treatment plans of CT images centred on the beam with mice positioned head down while mice were positioned upside down to avoid exposing small intestines to IR.

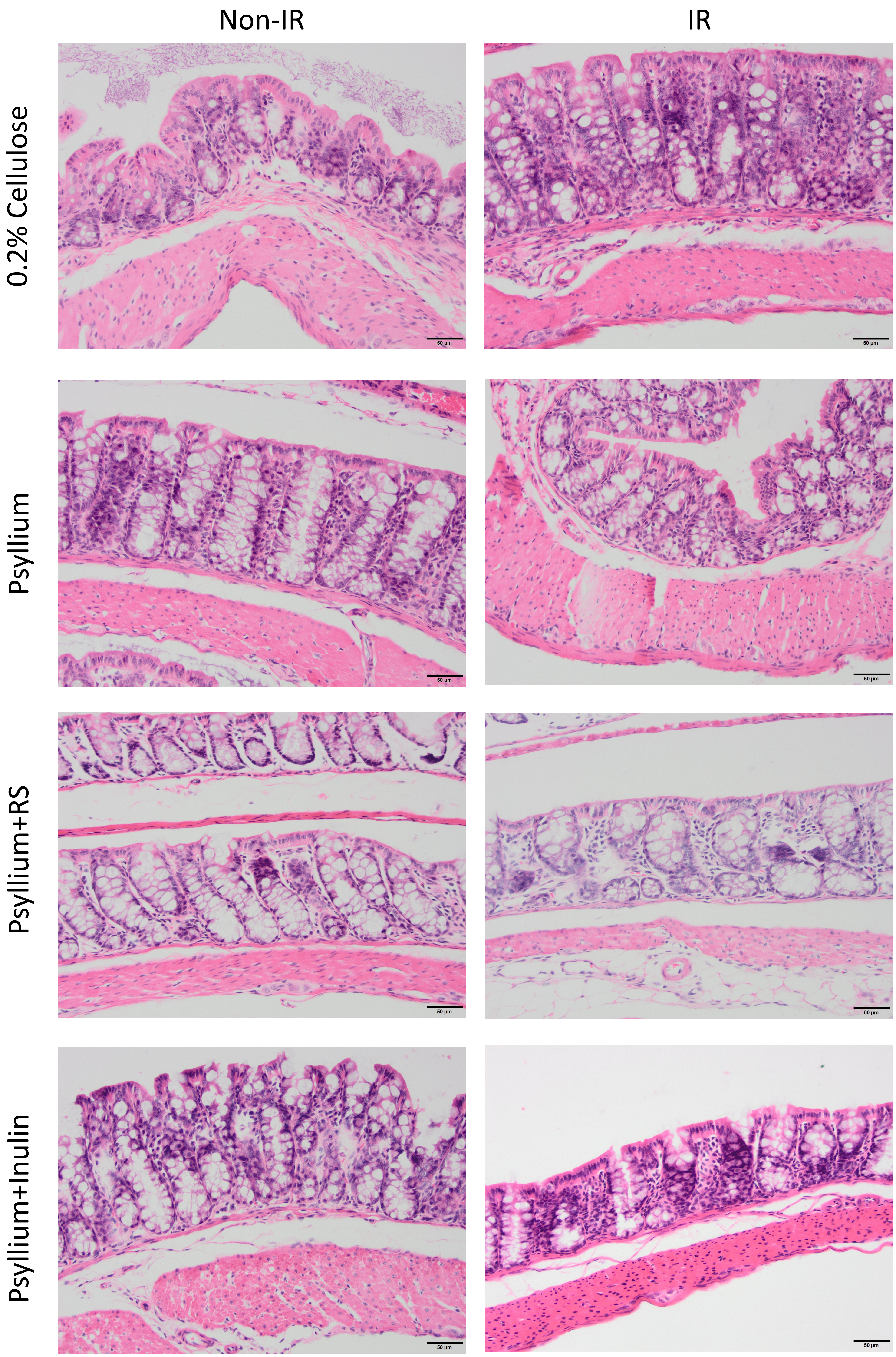

Supplementary Fig. 25 Representative images of mouse large intestine sections in non-tumour-bearing mice after 22-week modified diet with or without SARRP IR. The presence of inflammation and fibrosis was assessed at 20-weeks post-irradiation of all dietary groups on 'Swiss rolls' sections that were stained by Haematoxylin & eosin. The images were taken at x200 magnification and the bar = 50 µm.

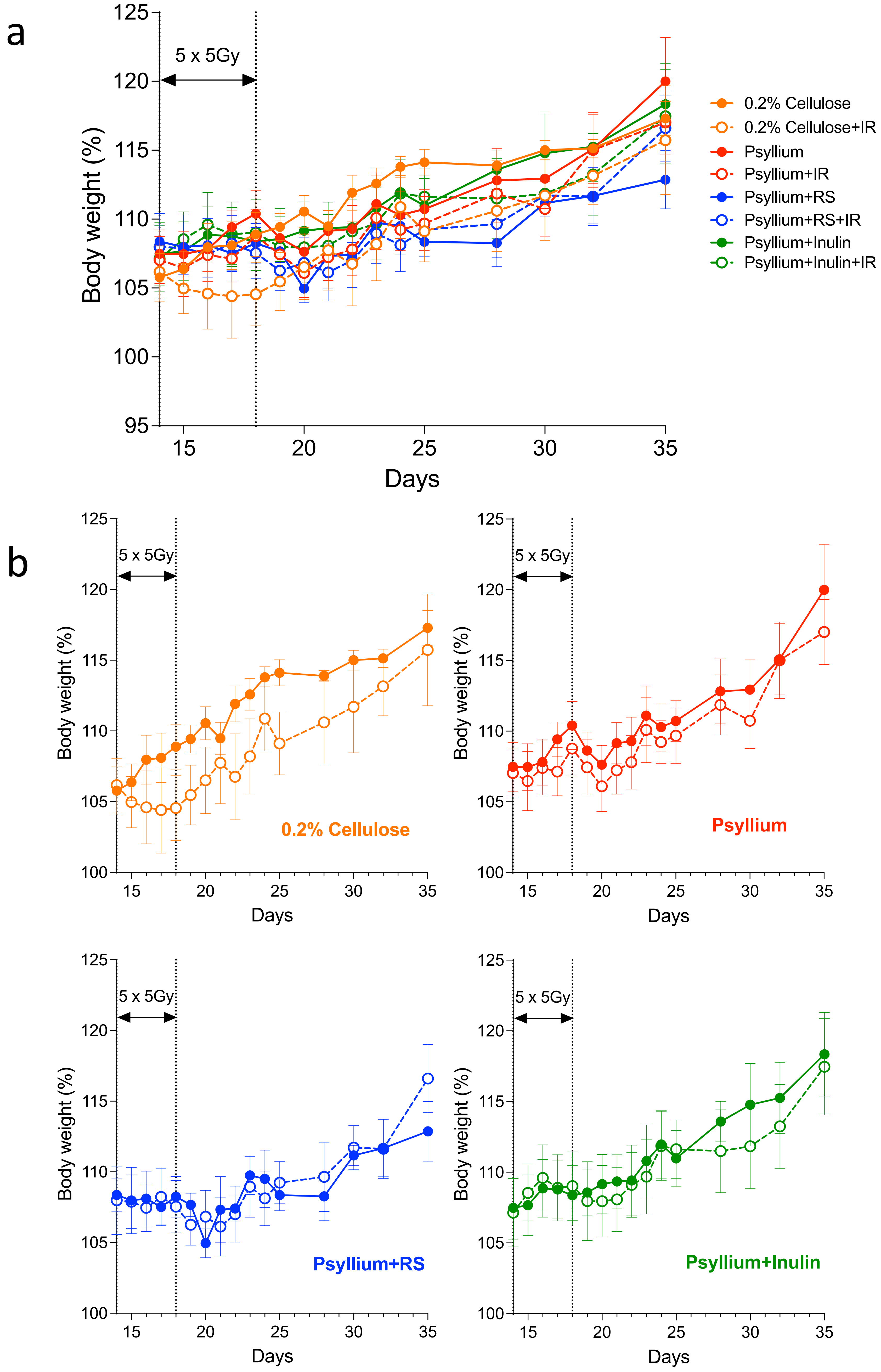

Supplementary Fig. 26 Relative body weight of non-IR and IR cohorts of each dietary groups for the mice that did not receive IR or following IR. Body weight curves of (a) all and (b) single dietary groups were shown. The x-axis was the days after starting the modified diets. Data are presented as mean ± SEM.

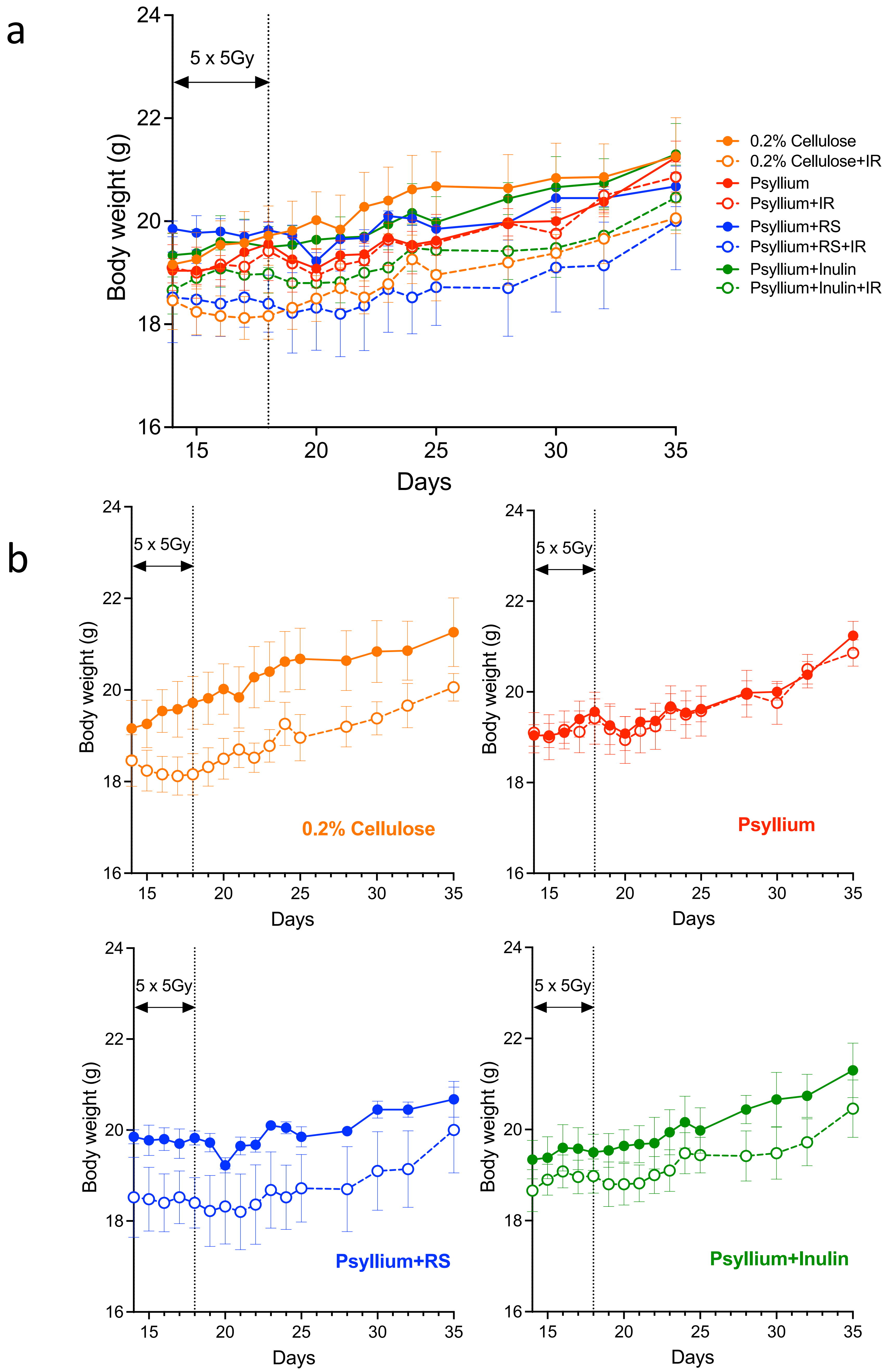

Supplementary Fig. 27 Actual body weight of non-IR and IR cohorts of each dietary groups for the mice that did not receive IR or following IR. Body weight curves of (a) all and (b) single dietary groups were shown. The x-axis was the days after starting the modified diets. Data are presented as mean ± SEM.

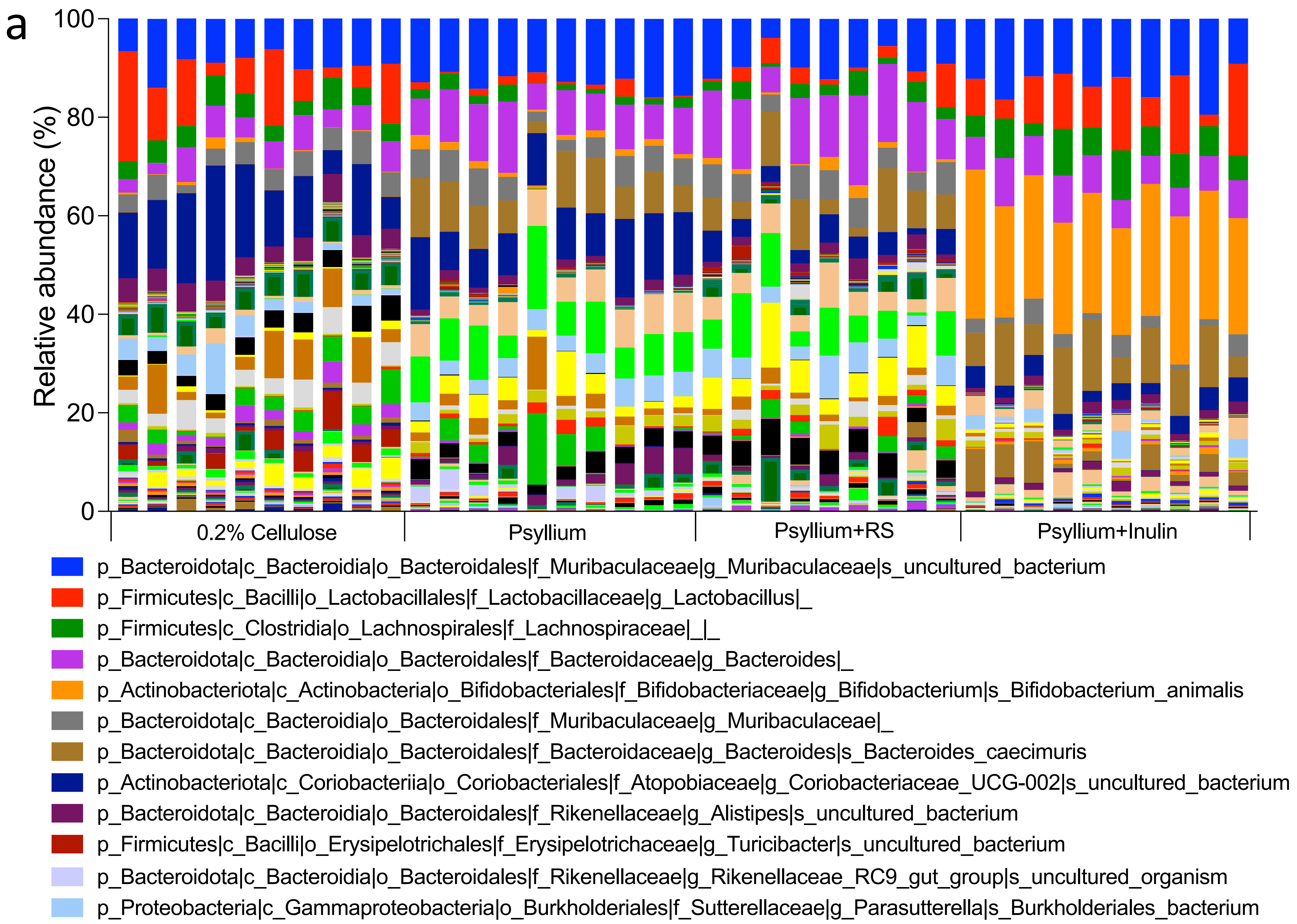
a
Relative abundance (%)
0
20
40
60
80
100
0.2% Cellulose
Psyllium
Psyllium+RS
Psyllium+Inulin
p_Bacteroidota|c_Bacteroidia|o_Bacteroidales|f_Muribaculaceae|g_Muribaculaceae|s_uncultured_bacterium
p_Firmicutes|c_Bacilli|o_Lactobacillales|f_Lactobacillaceae|g_Lactobacillus|_
p_Firmicutes|c_Clostridia|o_Lachnospirales|f_Lachnospiraceae|_|_
p_Bacteroidota|c_Bacteroidia|o_Bacteroidales|f_Bacteroidaceae|g_Bacteroides|_
p_Actinobacteriota|c_Actinobacteria|o_Bifidobacteriales|f_Bifidobacteriaceae|g_Bifidobacterium|s_Bifidobacterium_animalis
p_Bacteroidota|c_Bacteroidia|o_Bacteroidales|f_Muribaculaceae|g_Muribaculaceae|_
p_Bacteroidota|c_Bacteroidia|o_Bacteroidales|f_Bacteroidaceae|g_Bacteroides|s_Bacteroides_caecimuris
p_Actinobacteriota|c_Coriobacteriia|o_Coriobacteriales|f_Atopobiaceae|g_Coriobacteriaceae_UCG-002|s_uncultured_bacterium
p_Bacteroidota|c_Bacteroidia|o_Bacteroidales|f_Rikenellaceae|g_Alistipes|s_uncultured_bacterium
p_Firmicutes|c_Bacilli|o_Erysipelotrichales|f_Erysipelotrichaceae|g_Turicibacter|s_uncultured_bacterium
p_Bacteroidota|c_Bacteroidia|o_Bacteroidales|f_Rikenellaceae|g_Rikenellaceae_RC9_gut_group|s_uncultured_organism
p_Proteobacteria|c_Gammaproteobacteria|o_Burkholderiales|f_Sutterellaceae|g_Parasutterella|s_Burkholderiales_bacterium
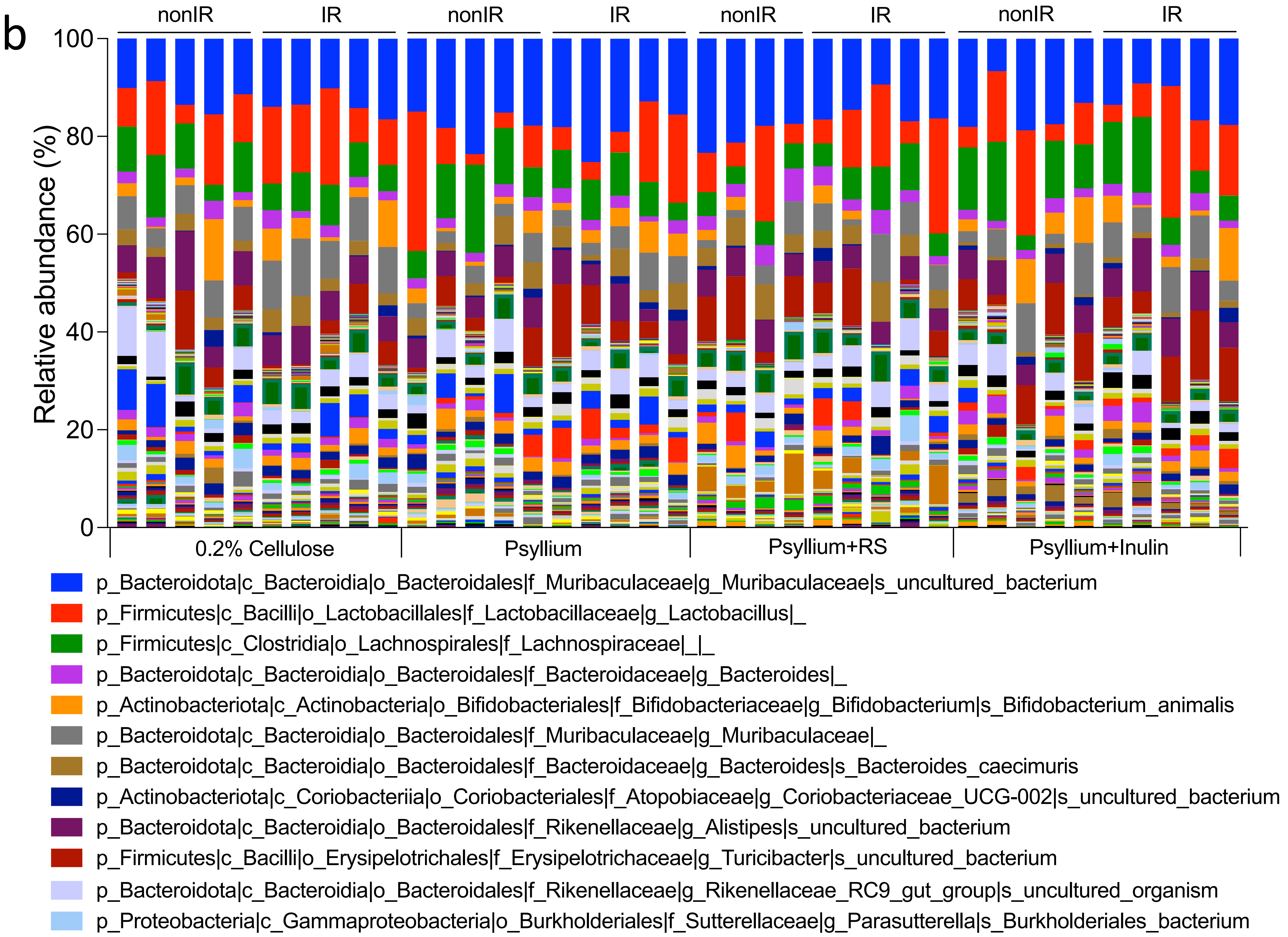
b
nonIR
IR
nonIR
IR
nonIR
IR
nonIR
IR
Relative abundance (%)
0
20
40
60
80
100
0.2% Cellulose
Psyllium
Psyllium+RS
Psyllium+Inulin
p_Bacteroidota|c_Bacteroidia|o_Bacteroidales|f_Muribaculaceae|g_Muribaculaceae|s_uncultured_bacterium
p_Firmicutes|c_Bacilli|o_Lactobacillales|f_Lactobacillaceae|g_Lactobacillus|_
p_Firmicutes|c_Clostridia|o_Lachnospirales|f_Lachnospiraceae|_|_
p_Bacteroidota|c_Bacteroidia|o_Bacteroidales|f_Bacteroidaceae|g_Bacteroides|_
p_Actinobacteriota|c_Actinobacteria|o_Bifidobacteriales|f_Bifidobacteriaceae|g_Bifidobacterium|s_Bifidobacterium_animalis
p_Bacteroidota|c_Bacteroidia|o_Bacteroidales|f_Muribaculaceae|g_Muribaculaceae|_
p_Bacteroidota|c_Bacteroidia|o_Bacteroidales|f_Bacteroidaceae|g_Bacteroides|s_Bacteroides_caecimuris
p_Actinobacteriota|c_Coriobacteriia|o_Coriobacteriales|f_Atopobiaceae|g_Coriobacteriaceae_UCG-002|s_uncultured_bacterium
p_Bacteroidota|c_Bacteroidia|o_Bacteroidales|f_Rikenellaceae|g_Alistipes|s_uncultured_bacterium
p_Firmicutes|c_Bacilli|o_Erysipelotrichales|f_Erysipelotrichaceae|g_Turicibacter|s_uncultured_bacterium
p_Bacteroidota|c_Bacteroidia|o_Bacteroidales|f_Rikenellaceae|g_Rikenellaceae_RC9_gut_group|s_uncultured_organism
p_Proteobacteria|c_Gammaproteobacteria|o_Burkholderiales|f_Sutterellaceae|g_Parasutterella|s_Burkholderiales_bacterium

Supplementary Fig. 28 Phylogenetic composition of faecal microbiota before and after irradiation in the late toxicity experiment. Faecal samples were collected (a) 9 weeks post modified diets (n=5/non-IR cohort, n=5/IR cohort in each dietary group) and (b) 5 weeks after changing back to normal chow. The samples were in the same order in the top and bottom panels.

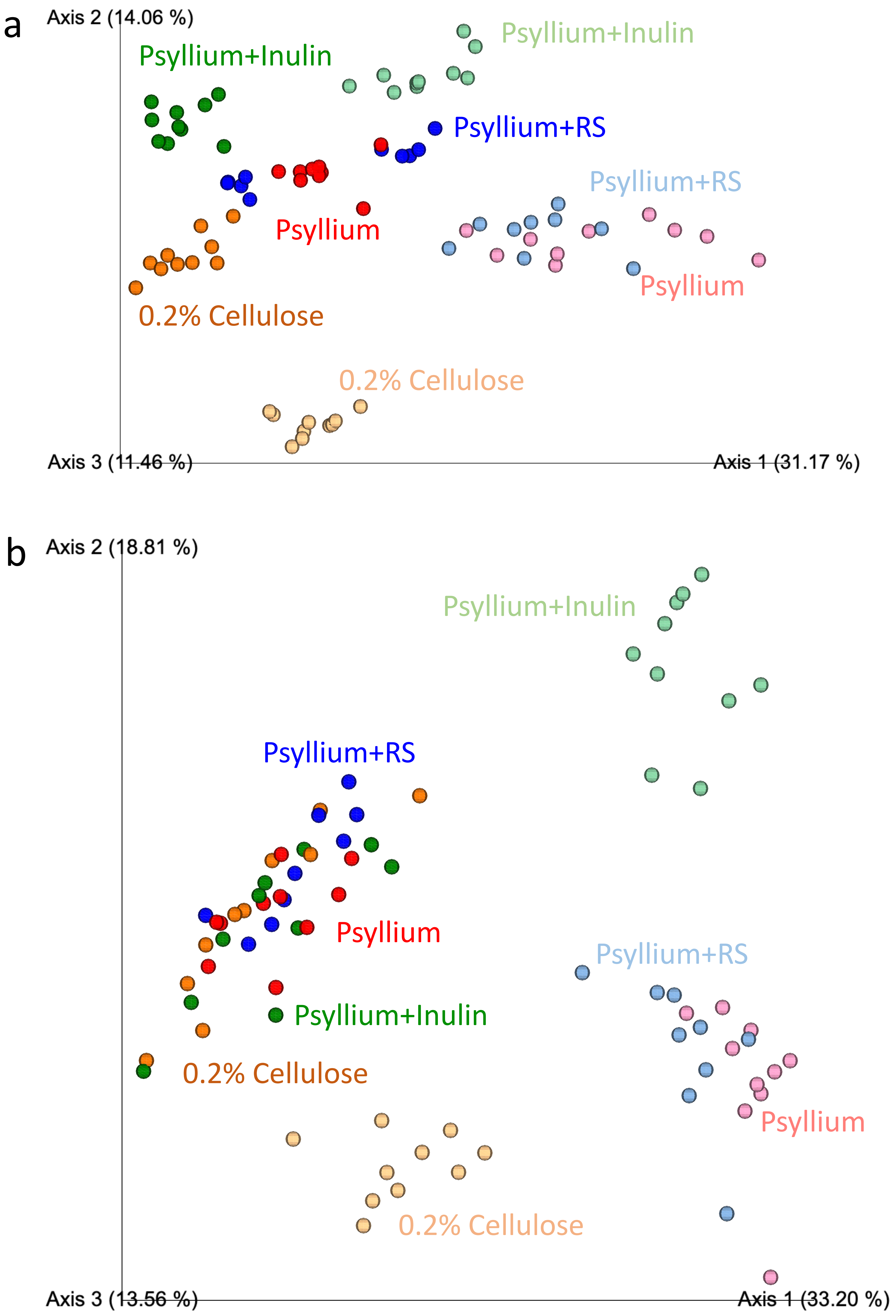

Supplementary Fig. 29 Beta diversity of faecal microbiota in the late toxicity experiment. Principal coordinate analysis using (a) unweighted and (b) weighted UniFrac of faecal microbiotas (n=5/non-IR cohort, n=5/IR cohort in each dietary group) that were collected 9 weeks post modified diets (light colours) and 5 weeks after changing back to normal chow (dark colours). ADONIS test was used to confirm the existence of significant group differences in terms of gut microbiota composition.

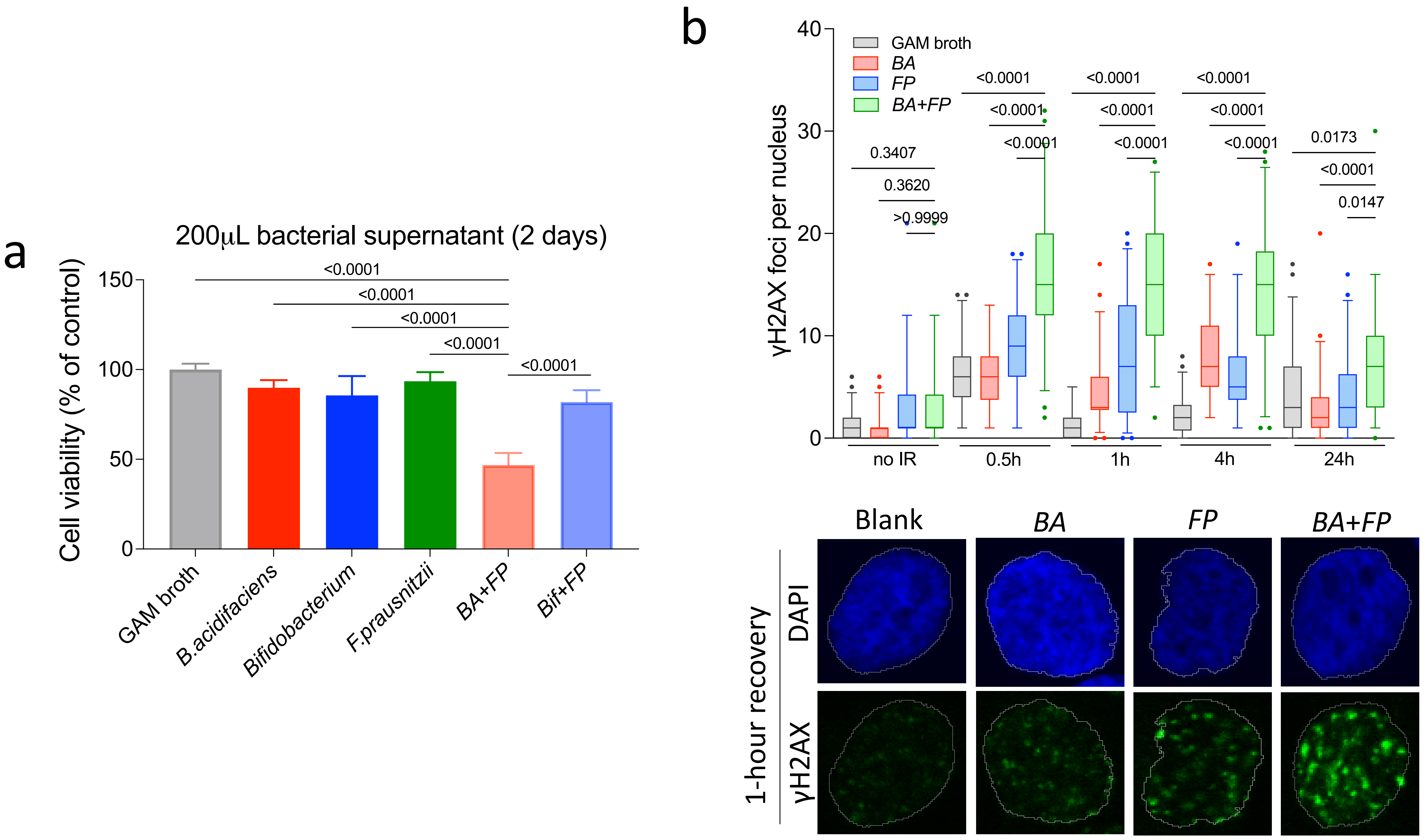

Supplementary Fig. 30 BA+FP increased cytotoxic responses and DNA damage in T24 bladder cancer cells. (a) The cell survival of T24 cells treated with 200 μL of GAM broth or bacterial supernatants, in 500 μL of medium for 2 days(N=3). (b) Immunofluorescence microscopy analysis of γ-H2AX levels (N=3) of T24 cells treated with 100 μL bacterial supernatants in 2 mL of medium for 24 hours. DNA damage was evaluated after treating with 2 Gy ionising radiation. pHs of GAM broth and bacterial supernatants were all neutralised to 7.2. *BA+FP* denotes the co-culture of *B.acidifaciens* and *F.prausnitzii*, while *Bif+FP* denotes the co-culture of *Bifidobacterium* and *F.prausnitzii.* One-way ANOVA with Bonferroni's multiple comparison test was used to compare the means among different dietary groups. Data are presented as mean ± SD.

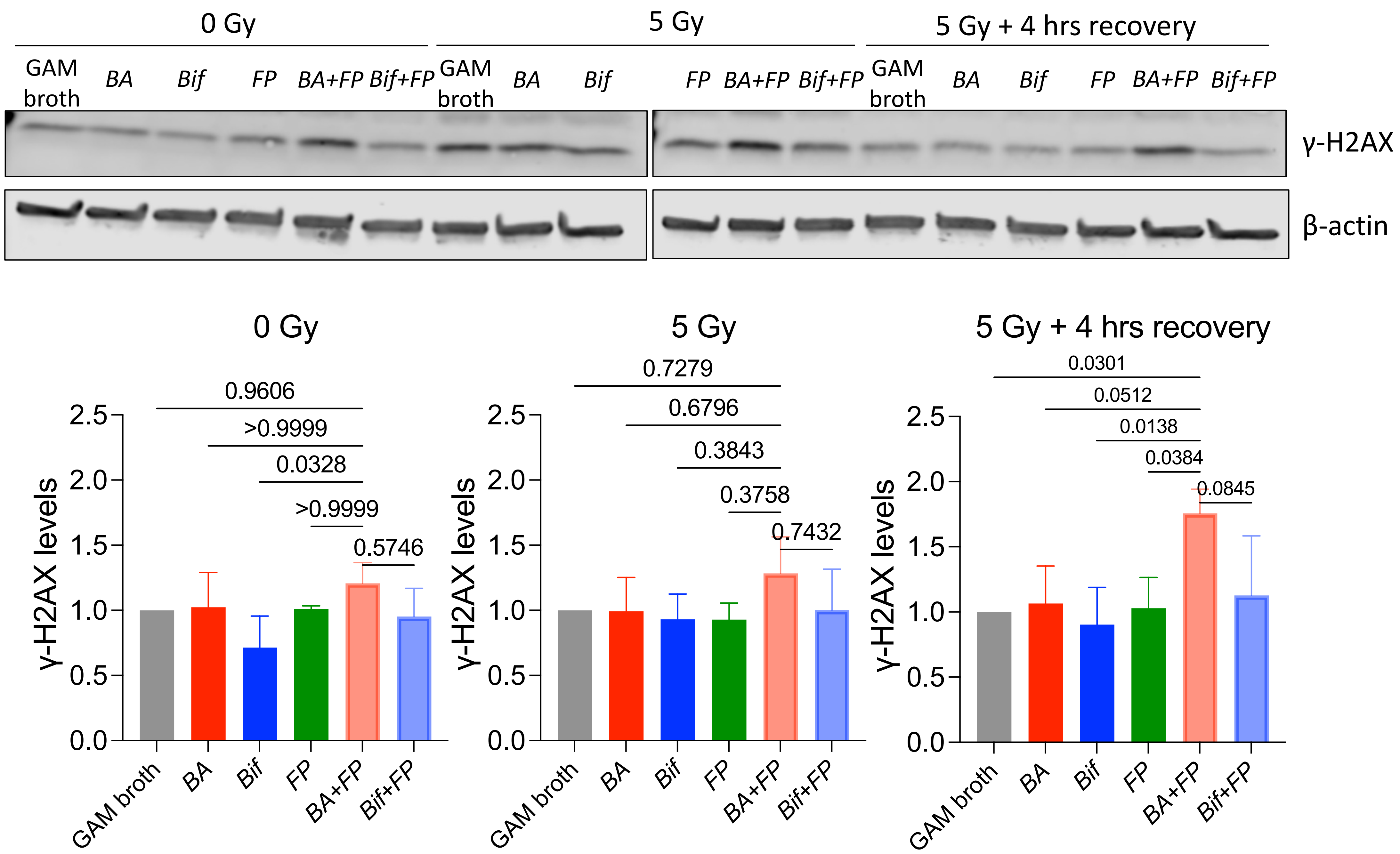

Supplementary Fig. 31 *BA+FP* increased histone acetylation levels and DNA damage in bladder cancer cells. Western blot analysis of γ-H2AX level (N=3) of RT112 cells treated with different bacterial supernatants in 6-well plates. DNA damage was evaluated after treating with 5 Gy ionising radiation. The samples derived from the same experiment and the blots were processed in parallel. pHs of GAM broth and all bacterial supernatants were neutralised to 7.2. *BA+FP* denotes the co-culture of *B. acidifaciens* and *F. prausnitzii*, while *Bif+FP* denotes the co-culture of *B. animalis* and *F. prausnitzii.* One-way ANOVA with Bonferroni's multiple comparison test was used to compare the means among different dietary groups. Data are presented as mean ± SD.

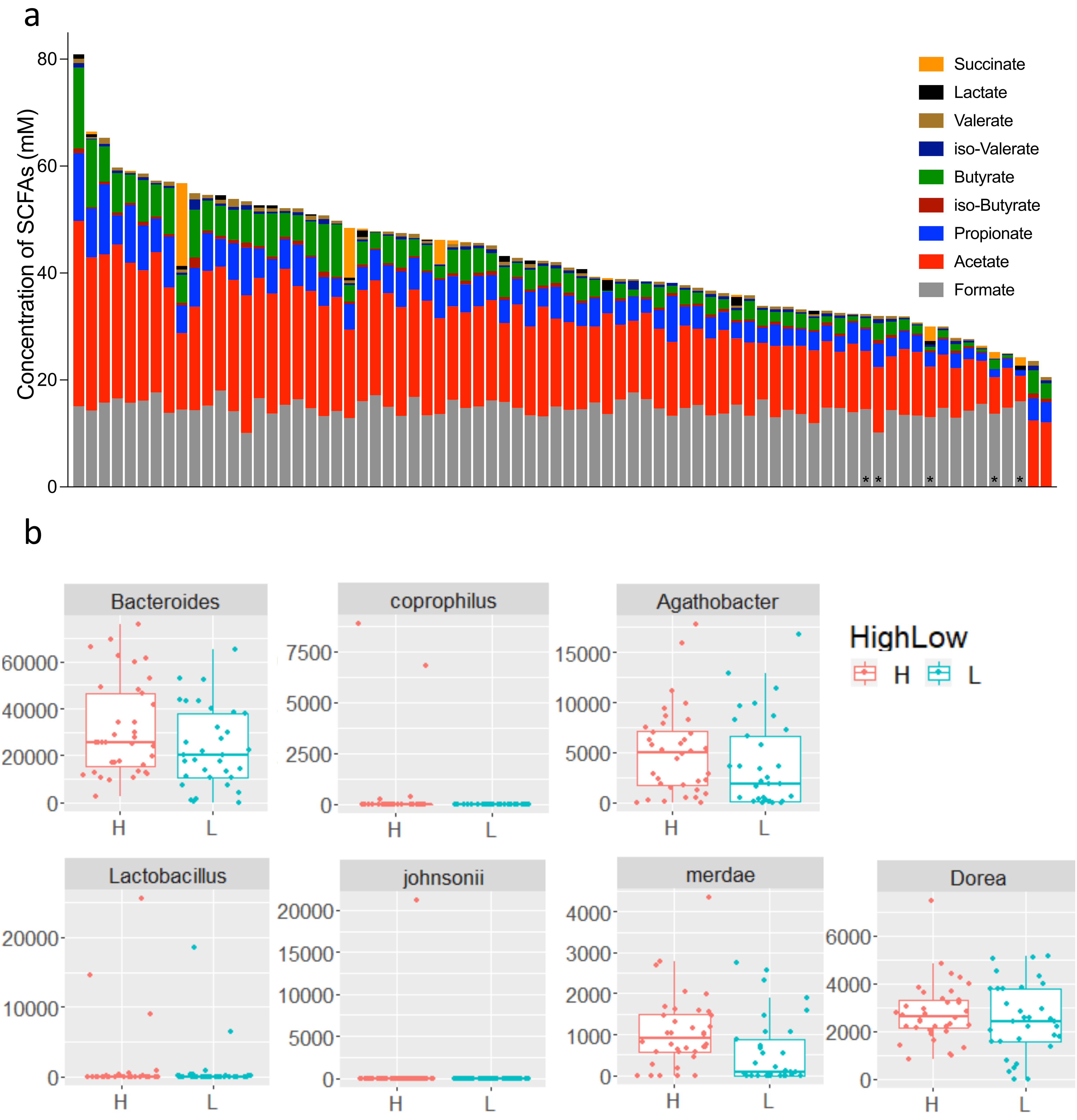

Supplementary Fig. 32 Production of SCFAs in pelvic cancer patients. (a) Concentrations of SCFAs in cancer patients. Asterisk indicates right sided colon samples. (b) Abundance of taxa found to be significantly associated with SCFA. Samples are grouped by high vs low faecal acetate, propionate and butyrate. The median of these three SCFAs combined was the cut off between high and low levels. Each dot is an individual sample.

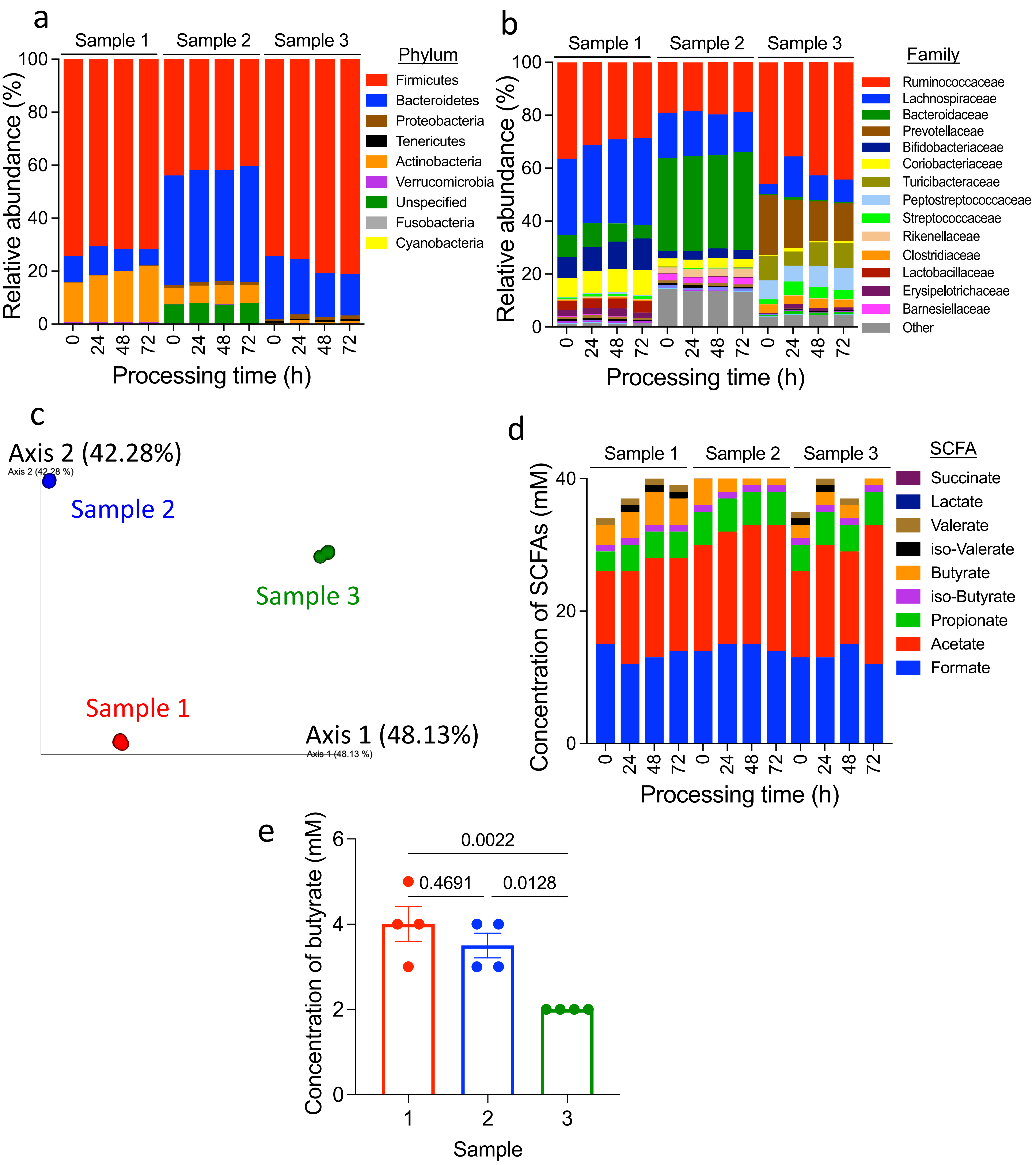

Supplementary Fig. 33 Comparison of bacterial and SCFA relative abundances between human samples processed serially showed similar profiles between the different processing times: 0, 24, 48 and 72 hours. (a) Relative abundances of bacteria at the phylum level for the serial samples. (b) Relative abundances of the 15 most abundant bacteria at the family level for the serial samples. (c) Principal coordinate analysis of the serial samples. ADONIS test was used to confirm the existence of significant group differences in terms of gut microbiota composition among the serial samples. (d) SCFA concentrations for the serial samples. (e) Among all SCFAs, only the concentration of butyrate was lower in sample 3 (p-value=0.0022 vs. sample 1 and =0.0128 vs. sample 2) despite a significant difference seen in gut microbiota profiles between individuals (p=0.001).

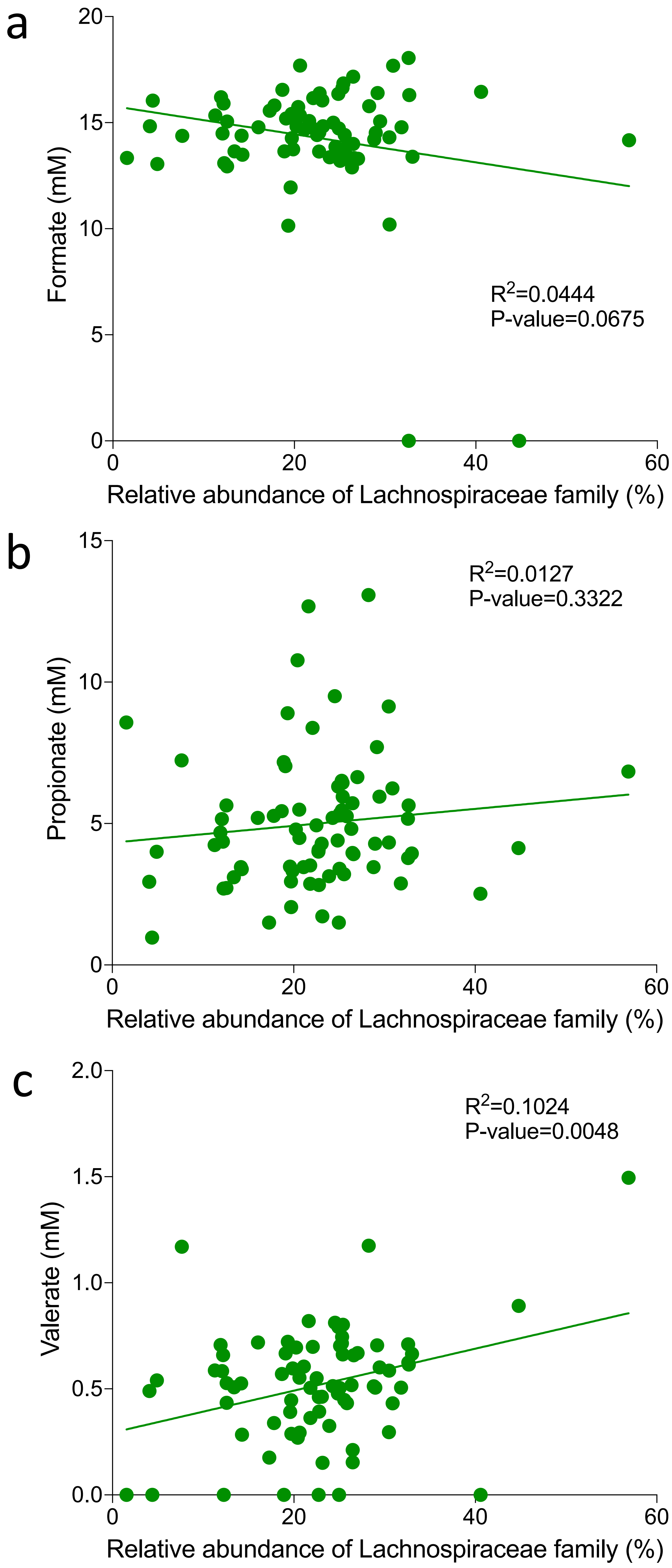

Supplementary Fig. 34 Correlations between Lachnospiraceae family and faecal (a) formate, (b) propionate, (c) valerate levels in cancer patients. The associations were assessed using the Pearson's correlation method.

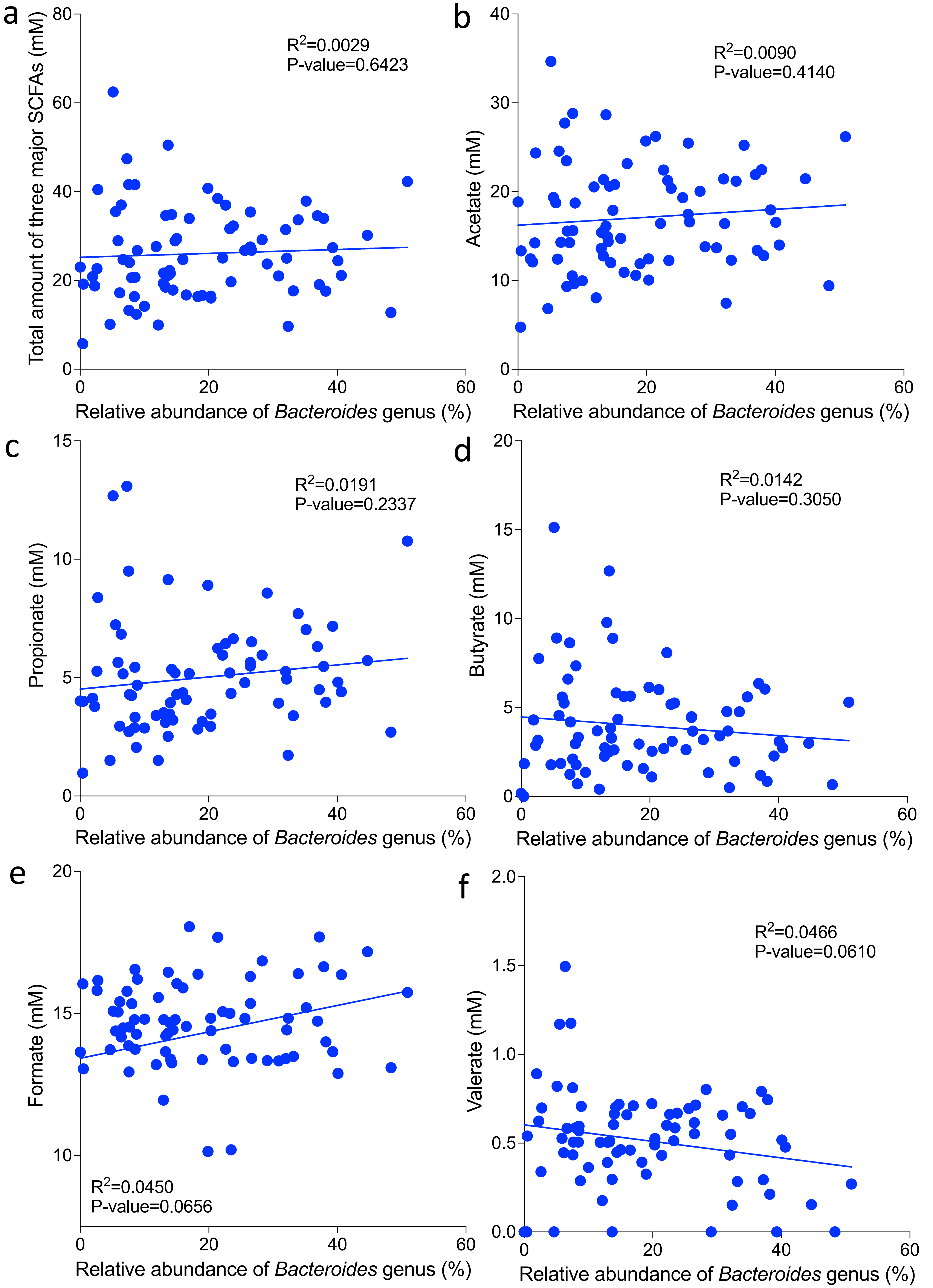

Supplementary Fig. 35 Correlations between *Bacteroides* genus and faecal (a) total amount of three major SCFAs, (b) acetate, (c) propionate, (d) butyrate, (e) formate, (f) valerate levels in cancer patients. The associations were assessed using the Pearson's correlation method.

Supplementary materials

Supplementary Table 1 R^2 and Pr(>F) values from ADONIS test assessing unweighted and weighted UniFrac distances.

		Unweighted UniFrac		Weighted UniFrac	
		R^2	Pr(>F)	R^2	Pr(>F)
Diet		0.6678	0.001	0.8407	0.001
Cages of	0.2% Cellulose	0.1536	0.273	0.1336	0.511
	Psyllium	0.2861	0.001	0.3556	0.006
	Psyllium+ RS	0.1915	0.024	0.1661	0.307
	Psyllium+ Inulin	0.2169	0.002	0.2137	0.071

Supplementary Table 2 Four most abundant phyla for human and mouse samples.

Species	Diet	Firmicutes	Bacteroidetes	Actinobacteria	Proteobacteria
Human	Not modified	60.4 (1.3)	29.7 (1.4)	5.3 (0.6)	3.8 (0.6)
Mouse	0.2% Cellulose	48.0 (1.1) ***	36.5 (1.1) **	11.2 (0.9) *	1.3 (0.1) ^{ns}
Mouse	Psyllium	28.0 (0.8) ***	44.4 (0.8) ***	9.4 (0.6) ^{ns}	16.0 (1.0) ***
Mouse	Psyllium+ RS	32.6 (1.2) ***	46.6 (1.1) ***	6.7 (0.5) ^{ns}	12.6 (1.0) ***
Mouse	Psyllium+ Inulin	22.3 (0.9) ***	38.1 (0.8) ***	31.0 (0.9) ***	7.3 (0.4) ^{ns}

Data are presented as mean and SEM (%), n=15 for the mice samples and n=76 for the human samples.
Analysed by two-way ANOVA with Tukey's *post hoc* test.
Asterisks indicate different from human at the same column: *P<0.05, **P<0.01, ***P<0.001.

Supplementary Table 3 Bacteria taxa enriched in cancer patients with low and high faecal SCFAs.

Concentrations of three major gut microbiota produced SCFAs including acetate, propionate and butyrate (mM)	Low		High	
	Average	SEM	Average	SEM
f_Bacteroidaceae|g_Bacteroides|s_Bacteroides_coprophilus	0.09	0.09	1.26	0.62
f_Lachnospiraceae|g_Agathobacter	1.75	0.34	2.98	0.45
f_Lactobacillaceae|g_Lactobacillus|s_Lactobacillus_johnsonii	0.00	0.00	0.81	0.52
f_Lachnospiraceae|g_Agathobacter|_	1.15	0.24	2.08	0.33
f_Tannerellaceae|g_Parabacteroides|s_Parabacteroides_merdae	0.31	0.08	0.82	0.19
f_Lachnospiraceae|g_Dorea	0.86	0.10	1.23	0.12
f_Prevotellaceae|g_Prevotellaceae_NK3B31_group	0.00	0.00	0.30	0.20
f_Lachnospiraceae|g_Dorea|s_uncultured_bacterium	0.32	0.05	0.58	0.09
f_Lactobacillaceae|g_Lactobacillus|s_Lactobacillus_ruminis	0.00	0.00	0.23	0.16
f_Clostridia_vadinBB60_group|g_Clostridia_vadinBB60_group|s_uncultured_bacterium	0.23	0.13	0.05	0.02
f_Erysipelotrichaceae|g_Turicibacter	0.42	0.26	0.28	0.10
f_Lachnospiraceae|g_Frisingicoccus|s_uncultured_organism	0.00	0.00	0.00	0.00
f_Izemoplasmatales|g_Izemoplasmatales|s_uncultured_organism	0.29	0.15	0.01	0.00
f_Oscillospiraceae|g_UCG_002|_	1.14	0.24	0.63	0.09
f_Oscillospiraceae|g_UCG_002	2.36	0.38	1.28	0.18
f_Rikenellaceae|g_Alistipes|_	1.71	0.55	0.44	0.26
f_Oscillospiraceae	4.73	0.52	3.07	0.39
f_Streptococcaceae	4.09	1.64	1.76	0.72
f_Streptococcaceae|g_Streptococcus	4.09	1.64	1.76	0.72

Median of the three SCFAs combined was the cut off between high and low levels.

Supplementary Table 4 Four most abundant phyla of three serial human faecal samples prepared at 24-hour intervals for 72-hours.

Sample	Processing time (Hours)	Firmicutes (%)	Bacteroidetes (%)	Actinobacteria (%)	Proteobacteria (%)
S1	0	74.9	9.9	15.1	0.2
	24	71.1	10.9	17.8	0.2
	48	72.1	8.5	19.3	0.1
	72	72.1	6.3	21.5	0.1
S2	0	47.5	44.6	6.4	1.5
	24	45.3	46.3	7.0	1.4
	48	45.1	45.6	7.8	1.5
	72	43.7	47.8	7.2	1.3
S3	0	74.9	24.1	0.3	0.7
	24	75.8	21.0	1.2	1.9
	48	81.5	16.7	0.6	1.2
	72	81.9	15.9	0.8	1.5

Supplementary Table 5 Rodent diets without corn starch used in the study with varying levels of cellulose, psyllium, psyllium plus resistant starch, or inulin per 4000 kcal.

	Low fibre		High fibre (Psyllium)		High fibre (Psyllium+RS)		High fibre (Psyllium+Inulin)	
	2 gm Cellulose/4000kcal		50 gm Psyllium/4000kcal		50 gm Psyllium + 100 gm RS/4000kcal		50 gm Psyllium + 100 gm Inulin/4000kcal	
	gm%	kcal%	gm%	kcal%	gm%	kcal%	gm%	kcal%
Protein	21	20	21	20	19	20	19	20
Carbohydrate	67	64	63	64	65	64	56	59
Fat	7	16	7	16	7	16	7	16
Total		100		100		100		95
kcal/gm	4.20		4.05		3.84		3.81	
**Ingredient**	**gm**	**kcal**	**gm**	**kcal**	**gm**	**kcal**	**gm**	**kcal**
Casein	200	800	200	800	200	800	200	800
L-Cystein	3	12	3	12	3	12	3	12
Dextrose, Monohydrate	397.49	1590	384.99	1540	322.69	1291	347.49	1390
Hi-Maize Corn Starch 260 (56% RS)	0	0	0	0	179	501	0	0
Maltodextrin 10	132	528	132	528	132	528	132	528
Sucrose	100	400	100	400	100	400	100	400
Cellulose, BM200	2	0	0	0	0	0	0	0
Inulin (Orafti HP)	0	0	0	0	0	0	100	150
Psyllium (AEP Colloids)	0	0	50	50	50	50	50	50
Soybean oil	70	630	70	630	70	630	70	630
t-Butylhydroquinone	0.014	0	0.014	0	0.014	0	0.014	0
Mineral mix S10022G	35	0	35	0	35	0	35	0
Vitamin mix V10037	10	40	10	40	10	40	10	40
Choline bitartrate	2.5	0	2.5	0	2.5	0	2.5	0
**Total**	**952**	**4000**	**987.5**	**4000**	**1041.21**	**4000**	**1031.25**	**4000**
Cellulose (gm/kg diet)	2.1		0		0		0	
Psyllium (gm/kg diet)	0		50.6		48		47.6	
Resistant starch (gm/kg diet)	0		0		96		0	
Inulin (gm/kg diet)	0		0		0		100	

The content of resistant starch is based on the CoA of Hi-Maize Corn Starch, which has 62.8% TDF-dry basis and 11.7% moisture.

Supplementary Table 6 Definitions of immune cell populations based on expression of cell surface markers.

Immune cell population	Cell surface markers
Total leukocytes	$CD45^+$
T lymphocytes	$CD45^+$ $CD3^+$
CD8 T cells	$CD45^+$ $CD3^+$ $CD8^+$
CD4 T cells	$CD45^+$ $CD3^+$ $CD4^+$
B lymphocytes	$CD45^+$ $CD19^+$
Natural killer (NK) cells	$CD45^+$ $CD49b^+$
Macrophages	$CD45^+$ $F4/80^+$
Dendritic cells (DC)	$CD45^+$ $CD11c^+$
Immature myeloid cells	$CD45^+$ $Gr\text{-}1^+$ $CD11b^+$

Supplementary Table 7 Antibody titrations and catalogue numbers.

Fluorophore	Myeloid panel	Lymphoid panel
416/451	Fixable Violet Dead Cell Stain Kit (1:650; L34963)	
AF700	CD45 (1:40; eBioscience 56-0451-82)	
APC	Ly6G (1:650; eBioscience 17-5931-82)	CD49b (1:80; Cat #17-5971-82)
PE-Cy7	CD11b (1:10000; Cat #25-0112-82)	CD3 (1:40; eBioscience 25-0031-81)
FITC	CD11c (1:800; Cat #11-0114-82)	CD8 (1:160; eBioscience 11-0081-82)
PE	F4/8b (1:650; eBioscience 12-4801-82)	CD19 (1:650; Cat #12-0193-82)
PerCP-Cy5.5	Gr-1 (1:650; Cat #45-5931-80)	CD4 (1:400; Cat #45-0042-82)

Supplementary Table 8 Effect of technical factors on sample composition (Bray-Curtis)

**Factor**	**R^2**	**Pr(>F)**	
Date of processing	0.7328	0.0221	*
Sex	0.0158	0.1658	
Date of DNA extraction	0.1749	0.6244	
Days before DNA extraction	0.0133	0.8753	
Extraction kit	0.0144	0.5514	
The person processing	0.0433	0.5918	
Hours from when sample produced	0.0148	0.5573	
